# Amine-linked diglycosides: Synthesis facilitated by the enhanced reactivity of allylic electrophiles, and glycosidase inhibition assays

**DOI:** 10.3762/bjoc.7.128

**Published:** 2011-08-16

**Authors:** Ian Cumpstey, Jens Frigell, Elias Pershagen, Tashfeen Akhtar, Elena Moreno-Clavijo, Inmaculada Robina, Dominic S Alonzi, Terry D Butters

**Affiliations:** 1 Department of Organic Chemistry, The Arrhenius Laboratory, Stockholm University, 106 91 Stockholm, Sweden; 2 Institut de Chimie des Substances Naturelles, Centre National de la Recherche Scientifique, 91198 Gif-sur-Yvette CEDEX, France; 3 Department of Organic Chemistry, Faculty of Chemistry, University of Seville, Prof. García González, 1, 41012 Seville, Spain; 4 Glycobiology Institute, Department of Biochemistry, Oxford University, South Parks Road, Oxford, OX1 3QU, England

**Keywords:** amination, glycomimetics, glycosidases, Mitsunobu, pseudodisaccharides

## Abstract

Diglycose derivatives, consisting of two monosaccharides linked at non-anomeric positions by a bridging nitrogen atom, have been synthesised. Conversion of one of the precursor monosaccharide coupling components into an unsaturated derivative enhances its electrophilicity at the allylic position, facilitating coupling reactions. Mitsunobu coupling between nosylamides and 2,3-unsaturated-4-alcohols gave the 4-amino-pseudodisaccharides with inversion of configuration as single regio- and diastereoisomers. A palladium-catalysed coupling between an amine and a 2,3-unsaturated 4-trichloroacetimidate gave a 2-amino-pseudodisaccharide as the major product, along with other minor products. Derivatisation of the C=C double bond in pseudodisaccharides allowed the formation of Man(*N*4–6)Glc and Man(*N*4–6)Man diglycosides. The amine-linked diglycosides were found to show weak glycosidase inhibitory activity.

## Introduction

We have been interested in synthesising molecules consisting of two monosaccharides linked by formal condensation without using the anomeric position [1]. Such molecules, termed diglycoses (or neodisaccharides), are linked by ether, amine, thioether, selenoether, etc., bridges, and so are presumably more stable to hydrolysis by acid or glycosidases than (glycosidic) disaccharides. Diglycoses have many features in common with disaccharides, with a similar general appearance, size, and functional group pattern, i.e., polyhydroxylation with potentially hydrophobic areas above and below the ring plane. Synthetic structures of this type have been known since the 1960s [2–3], and have attracted sporadic attention since then, often in the context of proposed carbohydrate or disaccharide mimicry. Last year, we reported that neutral ether- and thioether-linked diglycose derivatives can interact with lectins with affinities similar to those of strongly binding disaccharide ligands [4]. In the related carbasugar series, Ogawa et al. have shown that pseudodisaccharides with a bridging nitrogen atom bind more strongly to glycosidases than do the corresponding ether or thioether derivatives [5]. It follows that amine-linked diglycose derivatives may act as glycosidase inhibitors. We set about the synthesis of some compounds of this type to test this hypothesis.

In our initial investigations into this area [6] we found that the installation of amine linkages between primary–primary carbons of monosaccharides was relatively straightforward; this was achieved by Mitsunobu coupling between carbohydrate C-6 alcohols and carbohydrate C-6 sulfonamides. Primary–secondary linkages were more difficult to synthesise; attempted Mitsunobu coupling failed, but we synthesised such amines by epoxide-opening. We did not achieve the formation of *sec*–*sec* linked secondary amines. The synthesis of a *sec*–*sec* amine-linked diglycoside structure by epoxide-opening was reported by Coxon [7], its formation being achieved in low yield (25%) under rather harsh conditions (autoclave, 140 °C), while a similar epoxide-opening reaction starting from a rather more complex substrate has been used to synthesise a pseudohexasaccharide in very low yield (12%) [8]. Kroutil et al. reported the synthesis of a number of aminated amine-linked *sec–sec* structures by aziridine-opening reactions (ionic liquid, 120 °C) [9]. Primary–*sec* amine-linked structures have been synthesised by Thiem et al., through a reductive amination strategy [10–11].

In contrast, related structures containing an amine linkage between a carbohydrate ring carbon (i.e., a secondary position) and C-1 of either a (C-5a methylene) carbasugar or a C5=C5a unsaturated carbasugar, are relatively common [12]. There are two possible contributing factors to this: First, these classes of carbasugar or valienamine structures have been more widely studied, possibly due to their presence in natural products and well known biological activities as glycosidase inhibitors [13]; second, electrophiles that are either lacking a bulky and electron-withdrawing substituent at one beta position [14] (making them less carbohydrate-like), or that are allylic [15], would tend to be intrinsically more reactive than similar carbohydrate electrophiles, thus facilitating the coupling reaction.

**Scheme 1:**
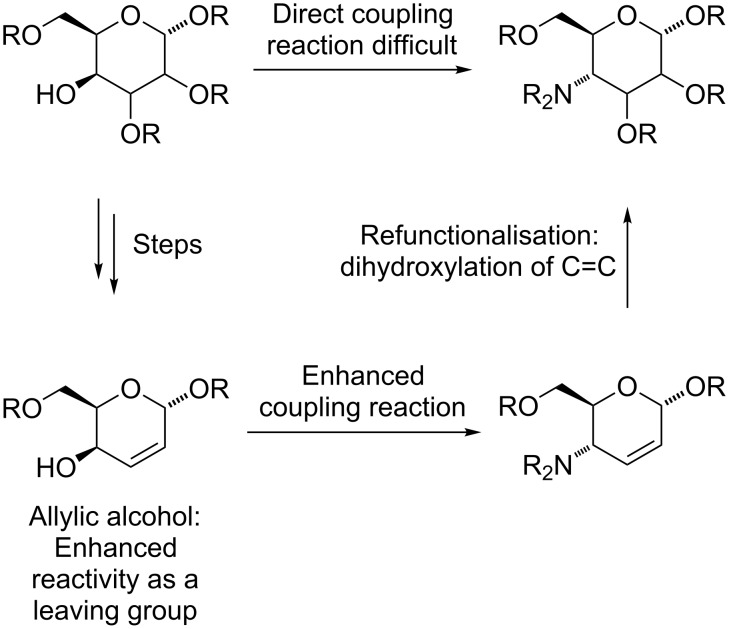
The concept of using allylic reactivity enhancement to facilitate diglycoside synthesis.

Bearing in mind the limitations discovered in our earlier work, we planned to enhance the reactivity of the electrophilic component in our coupling reactions by converting a carbohydrate into an unsaturated derivative with an allylic alcohol as leaving group [16]. After the coupling reaction, dihydroxylation of the C=C double bond would restore the carbohydrate structure (Scheme 1) [17]. As well as Mitsunobu chemistry [18], the allylic nature of the electrophile opens up the way for transition-metal-catalysed allylic amination reactions [19–21] as a possible coupling method. We report our investigations into this area in this paper.

There is extensive coverage in the literature of the derivatisation of primary carbohydrate alcohols by Mitsunobu type reactions [22]. Reports of Mitsunobu reactions of secondary alcohols in fully functionalized carbohydrates are much scarcer. Rather, reports exist of failed attempts at Mitsunobu reactions of secondary carbohydrate alcohols [23] or the selective functionalisation of primary carbohydrate alcohols in the presence of secondary alcohols [24]. Some examples of successful reactions do exist, though, for oxygen, nitrogen and sulfur nucleophiles [25–28]. Unsaturated carbohydrates, similar to those described here, have also been reported to undergo Mitsunobu reaction with simple, non-carbohydrate nucleophiles [29–30]. The palladium-catalysed allylic amination reaction on unsaturated pyranose rings was pioneered thirty years ago by Hanna and Baer [19–20], and has more recently been reinvestigated with rather simple nitrogen nucleophiles [30–31]. Carbohydrate amines have been used as nucleophiles in allylic amination by Shing to form valienamine pseudodisaccharides [21].

## Results and Discussion

**Scheme 2:**
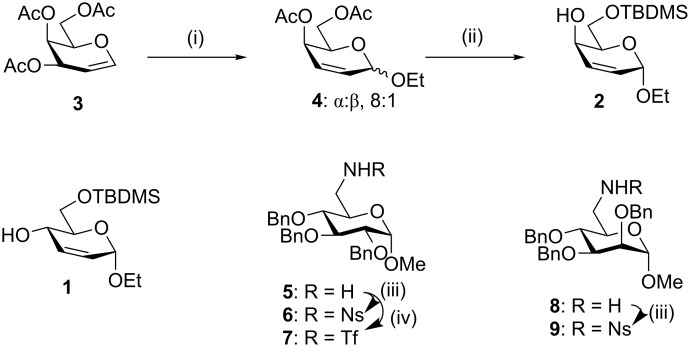
(i) Phosphomolybdic acid, EtOH, MeCN, 0 °C→RT, 63%; (ii) a) NaOMe, MeOH, 87%; b) TBDMSCl, imidazole, DMF, 0 °C→RT, 68%; (iii) NsCl, Et_3_N, DMAP, CH_2_Cl_2_; **6**, 97% [6]; **9**, 88%; (iv) Tf_2_O, Et_3_N, CH_2_Cl_2_, −5 °C, 64% [6].

The synthesis of the 2,3-unsaturated *erythro* carbohydrate derivative **1** was carried out from triacetyl glucal essentially according to the literature procedure [30]. The *threo* alcohol **2** has been previously synthesised by Mitsunobu inversion of the *erythro* alcohol **1** [30]. We synthesised **2** from triacetyl galactal **3** by an analogous route to that used for the *erythro* alcohol **1** (Scheme 2). We found that the choice of Lewis acid used in the Ferrier reaction of **3** with ethanol was critical for a satisfactory yield of the unsaturated glycoside **4** to be achieved; phosphomolybdic acid [32] gave the product (α:β, 8:1) in 63% yield. Deacetylation of **4** and regioselective silylation of the primary alcohol gave the *threo* allylic alcohol **2**. The sulfonamide nucleophiles **6**, **7** and **9** were prepared from the corresponding amines **5** [33] and **8** [34], as described previously [6], with only one equivalent of the sulfonylating agent so as to avoid bis-sulfonamide formation.

**Scheme 3:**
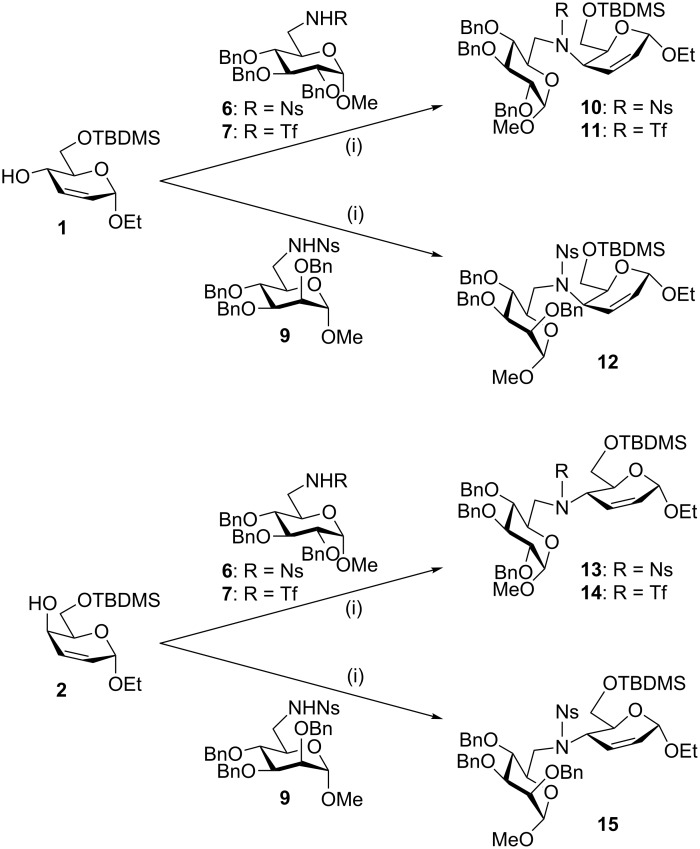
(i) DIAD, PPh_3_, THF, 0 °C→RT; **10**, 91%; **11**, 88%; **12**, 76%; **13**, 91%; **14**, 91%; **15**, 71%.

Mixing equimolar equivalents of the *erythro* allylic alcohol **1** and the glucose-6-nosylamide **6** with DIAD and triphenylphosphine resulted in a smooth coupling reaction to give the pseudodisaccharide **10** in high yield (Scheme 3). The alcohol **1** also underwent similar coupling with the glucose-6-triflamide **7** and the mannose-6-nosylamide **9** under the same Mitsunobu conditions to give the respective diglycoside precursors **11** and **12** in excellent yields. These were the same reaction conditions that had failed to give coupling reactions between a primary alcohol and a sulfonamide at a secondary carbohydrate position, or a secondary alcohol and a sulfonamide at a primary carbohydrate position, in our earlier study [6]. Coupling of this *threo* alcohol **2** with sulfonamide nucleophiles (**6**, **7** and **9**) again gave the protected secondary amines (**13**–**15**) in excellent yield. No difference in the efficiency of the coupling reactions of the nosylamide **6** and the triflamide **7** was seen with either of the alcohols **1** or **2**.

Assignment of the NMR spectra of the coupling products **10–15** by the usual 2D methods (COSY, HSQC) gave an unambiguous indication that, in all cases, the nitrogen nucleophile had attacked C-4, as judged by an upfield shifted ^13^C signal, and that the unsaturated bond was between C-2 and C-3. Hence these reactions proceeded with essentially complete regioselectivity, with no S_N_2’ products arising from attack at C-2 being seen. The coupling reactions were stereospecific: The nucleophile **6** coupled with the *threo* alcohol **2** to give the coupling product as a single diastereomer **13**; but **6** also coupled with the diastereomeric *erythro* alcohol **1** to give a different product **10**, again as a single diastereomer. This is consistent with an S_N_2 pathway with inversion of configuration, which is as expected for Mitsunobu reactions. The same was true of the other sulfonamide nucleophiles, **7** and **9**. Moreover, the *erythro* configuration of coupling product **15** was proved by the large value of the *J*_4,5_ coupling constant (9.8 Hz), which can only be explained by a *trans* relationship of the 4-H and 5-H protons and a ^O^*H*_5_ half-chair conformation. The *J*_4,5_ coupling constants for the products assigned as *threo* were not always well resolved, but in **12**, a smaller coupling constant (ca. 4 Hz) was seen.

**Scheme 4:**
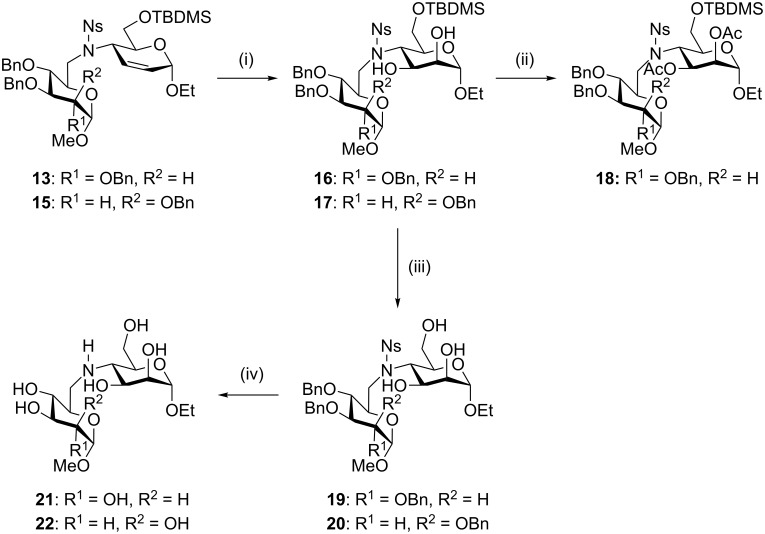
(i) K_2_OsO_4_, NMO, acetone, H_2_O; **16**, 88%; **17**, 73%; (ii) Ac_2_O, py, 83%; (iii) MeOH, HCl (1 M aq.); **19**, 74%; **20**, 86%; (iv) Na, NH_3_ (l), −78 °C; **21**, 78%; **22**, 53%.

Having achieved the coupling, we turned to the refunctionalisation of the C=C bond (Scheme 4). Osmium-catalysed dihydroxylation of the double bond in *erythro* configured pseudodisaccharide **13** proceeded to give a single diastereomer of product **16**. The configuration of the dihydroxylated product **16** was most conveniently assigned after conversion to its diacetate **18**, which resulted in a downfield shift of the 2-H and 3-H signals into a clear region of the ^1^H NMR spectrum, allowing readout of the coupling constants without any signal overlap. The values of *J*_1,2_ (1.8 Hz), *J*_2,3_ (3.3 Hz), and *J*_3,4_ (11.0 Hz) are consistent with the α-*manno* configuration, and inconsistent with the alternative α-*allo* configuration. Hence dihydroxylation proceeded from the opposite side to the two neighbouring substituents (at C-1 and C-4), as expected in a sterically controlled reaction, and consistent with previous results [35]. The protecting groups were then removed. First, the TBDMS ether was removed with acid to give the triol **19**. Alternatively, **19** was formed directly in the dihydroxylation reaction by allowing a longer reaction time (20 h) after quenching with NaHSO_3_, leading to a similar overall yield (80% from **13**). Attempted TBAF deprotection of the silyl ether in the dihydroxylation product **16** gave only a low yield of the triol **19**. Subsequently, the benzyl ethers and nosylamide in **19** were cleaved under Birch reduction conditions to furnish the free Man(*N*4–6)Glc diglycoside **21**. The other *erythro* configured pseudodisaccharide **15** behaved in a similar way, and we obtained the Man(*N*4–6)Man diglycoside **22** following the same reaction sequence (Scheme 4).

However, attempted functionalisation of the C=C double bond in the *threo* configured pseudodisaccharides **10** and **12** under the same conditions, or using stoichiometric K_2_OsO_4_ with NMO, failed to give any reaction. A combination of RuCl_3_ and NaIO_4_ produced, after 4 days, a mixture of starting material and desilylated material but no oxidised product. Treatment with mCPBA failed to give an epoxide, and after a week only some decomposition of the starting material was seen. Presumably, the *trans* relationship between the flanking NNsR and OEt groups effectively crowds both faces of the olefin, blocking its reactivity.

**Scheme 5:**
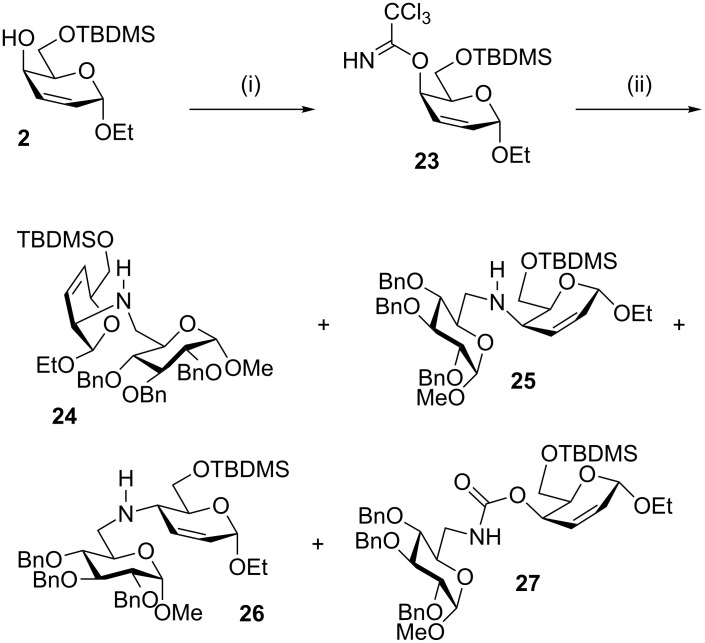
(i) Cl_3_CCN, DBU, CH_2_Cl_2_, 99%; (ii) TMPP, Pd(dba)_2_, **5**, Et_3_N, MeCN, **24**, 44%; **25**, 15%; **26**, 5%; **27**, 6%.

We briefly examined palladium-catalysed allylic amination as a possible coupling procedure. The allylic alcohol in the *threo* derivative **2** was converted into its trichloroacetimidate **23** by treatment with trichloroacetonitrile and base. The trichloroacetimidate is a leaving group that has the advantage of being easily and stereospecifically synthesised from the alcohol under basic conditions. We recently used the trichloroacetimidate as leaving group in palladium catalysed synthesis of carbasugar pseudodisaccharides [36]. Treatment of the imidate **23** with an amine nucleophile **5** [33] and a palladium catalyst [21] resulted in the formation of four products, **24** (44%), **25** (15%), **26** (5%) and **27** (6%), which were assigned the structures given in Scheme 5.

Three of **24–26** were isomeric, with m/z 734, consistent with the expected coupling products, i.e*.*, the allylic amines. The fourth product had m/z 800, consistent with the carbamate structure **27**. The determination of the regiochemistry of the unsaturated ring in **24** was complicated by long-range ^1^H–^1^H couplings and correlations in the COSY spectrum. It was difficult to distinguish between the C/H-2 and C/H-4 pairs of signals due to the presence of a cross-peak between 2-H and 5-H and the very low intensity of the cross-peaks between 1-H and both 2-H and 4-H, and between 4-H and 5-H. The regiochemistry was established by the presence of correlations between 6-H and C-4 in the HMBC spectrum. Hence, **24** was assigned a 2-amino-3,4-unsaturated structure.

The COSY spectrum of **25** was uncomplicated by any such long-range couplings, so the assignment of a 4-amino-2,3-unsaturated structure to **25** based on the COSY spectrum was straightforward. For **26**, the distinction between the C/H-2 and C/H-4 pairs of signals was based on the strong correlation between 5-H and 4-H, and the absence of any correlation between 5-H and 2-H. Correlations were seen between 1-H and each of 2-H, 3-H, and 4-H, although the correlation between 1-H and 2-H was much more intense than the others. Hence, **26** was assigned a 4-amino-2,3-unsaturated structure. The regiochemical assignments for both **25** and **26** were both confirmed by C-4→6-H correlations in the HMBC spectra.

Hanna and Baer observed [20] that 2,3-unsaturated-4-amino-α-glycopyranosides have ^13^C chemical shifts for C-1 in the range 93.8–94.1 ppm, irrespective of the C-4 configuration. In our assessment the C-1 chemical shifts for **25** (94.3) and **26** (94.1) are in broad agreement with these values, whereas **24** (98.9) has a significantly higher shift. However, while a survey of the literature reveals that 2-amino-3,4-unsaturated compounds with the α*-threo* configuration (i.e., consistent with **24**, as shown) have C-1 shifts in the range 96.7–99.3 ppm (12 examples) [19,30,37–41], the corresponding C-2 epimeric α*-erythro* configured structures have C-1 shifts in the range 93.9–95.6 (3 examples) [40,42–43], similar to the 2,3-unsaturated-4-amino compounds. Therefore, use of ^13^C C-1 chemical shift alone as a diagnostic tool for regiochemistry should be avoided.

A further characteristic feature that can be used to distinguish 2-amino-3,4-unsaturated-α*-threo* configured compounds from their α*-erythro* C-2 epimers is the *J*_1,2_ coupling constant. Ferrier pointed out that α*-threo* diastereomers have very small *J*_1,2_ coupling constants, i.e., 1-H usually appears as a singlet, whereas the α*-erythro* diastereomers have *J*_1,2_ between 3.5–4.5 Hz [44]. In **24**, 1-H appeared as a singlet, consistent with our assignment of α*-threo* stereochemistry. The C-4 stereochemistry of the two 4-amino derivatives **25** and **26** was assigned on the basis of the *J*_4,5_ vicinal coupling constants. For **25**, *J*_4,5_ = 3.2 Hz, consistent with a 4,5-*cis* (hence *threo*) configuration; for **26**, *J*_4,5_ = 10.0 Hz, consistent with a 4,5-*trans* (hence *erythro*) configuration in an ^O^*H*_5_ conformation.

Hence, the major products **24** and **25** from the palladium-catalysed reaction are those with overall retention of configuration as expected from the double displacement mechanism. Under these unoptimised reaction conditions though, the palladium-catalysed reaction was much less regioselective than the Mitsunobu couplings. Moreover, the major regioisomer in the palladium-catalysed reaction was the 2-amino sugar, whereas in the Mitsunobu reactions, the 4-amino sugars were formed exclusively. A small amount of stereochemical scrambling of an intermediate π-allyl palladium complex could explain the formation of **26**.

**Figure 1:**
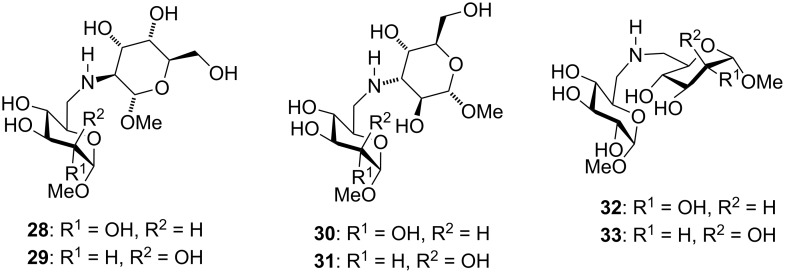
Previously synthesised amine-linked diglycosides.

Finally, we report the results of assays of the unprotected diglycosides Man(*N*4–6)Glc **21** and Man(*N*4–6)Man **22**, along with those we had synthesised earlier [6], viz., Alt(*N*2–6)Glc **28**, Alt(*N*2–6)Man **29**, Alt(*N*3–6)Glc **30**, Alt(*N*3–6)Man **31**, Glc(*N*6–6)Glc **32**, and Glc(*N*6–6)Man **33** (Figure 1), for inhibitory activity against glycosidases. Screening the compounds against twelve commercially available glycosidases (see Supporting Information File 1) [45–46], we found that none of the compounds had a high inhibitory activity against any of the glycosidases when tested at 1 mM inhibitor concentration. The results for those enzymes for which some inhibitory activity was seen arising from one or more of the diglycosides, are given in Table 1. These diglycosides showed weaker glycosidase inhibitory activity than the related *N*-substituted (monosaccharidic) altrosides recently reported by Jenkins et al. [47]. These results are in accordance with those reported for other disaccharide mimetics, such as aza-C-disaccharides, which showed no significant inhibition towards commercially available glycosidases [48].

**Table 1:** Inhibition of activity of commercially available glycosidases.

Glycosidase	Percentage inhibition at 1 mM inhibitor concentration^a^							

	**21**	**22**	**28**	**29**	**30**	**31**	**32**	**33**

α-L-fucosidase (bovine kidney)	16	NI	NI	NI	NI	NI	NI	NI
amyloglucosidase (Aspergillus niger)	NI	NI	19	NI	NI	NI	NI	NI
β-mannosidase (snail)	15	NI	NI	NI	NI	NI	15	16

^a^NI = no inhibition at 1 mM.

We also investigated inhibition of α-glucosidase II using the oligosaccharide Glc_2_Man_7_GlcNAc_2_ as substrate and using the free oligosaccharide (FOS) assay in cultured cells [49]. The Alt(*N*2–6)Man derivative **29** showed some inhibitory activity, with IC_50_ ca. 500 μM. None of the other compounds tested inhibited the enzyme at 100 μM concentration.

Such amine-linked diglycosides have not been shown to have intrinsic biological activity before. However, the inhibitory activity, where it exists, is weak for these compounds, and the nature of the binding and the basis for inhibition is not clear. α-Glucosidase II cleaves two linkages in nature in the biosynthesis of *N*-linked glycoproteins: Glc(α1→3)Glc and Glc(α1→3)Man. The Alt(*N*2–6)Man structure does not bear an immediately obvious resemblance to either of these substructures. Moreover, it has been proposed that a mannose-binding lectin domain of the α-glucosidase II β-subunit is also important for its activity [50], and the possibility that the inhibitor is actually binding not to the enzyme active site, but to this lectin domain instead, is not ruled out at present.

## Conclusion

Unsaturated carbohydrate derived electrophiles react readily with carbohydrate nitrogen nucleophiles to form amine-linked pseudodisaccharides. Triflamides and nosylamides were similarly effective as nucleophiles in Mitsunobu coupling reactions with allylic alcohols. The Mitsunobu coupling reactions proceeded with inversion of configuration, and S_N_2’ type reactions with C=C double bond migration were not observed. A palladium catalysed coupling reaction between an amine and an allylic trichloroacetimidate also gave a high yield of a mixture of amine-linked pseudodisaccharides, but under the conditions we used, much lower regioselectivity and diastereoselectivity were seen than in the Mitsunobu reactions. Completion of the diglycosides by refunctionalisation of the C=C double bond was successful for some diastereomers, but not for others. Glycosidase inhibitory activity of the compounds synthesised here, and also of structurally related compounds synthesised previously by us, was weak.

## Supporting Information

File 1 Experimental section; copies of ^1^H and ^13^C NMR spectra for new compounds.

File 2 ^1^H and ^13^C NMR spectra of compounds **9**–**17**.

File 3 ^1^H and ^13^C NMR spectra of compounds **18**–**27**.
