# Supplementary material for: Amine-linked diglycosides: Synthesis facilitated by the enhanced reactivity of allylic electrophiles, and glycosidase inhibition assays
Source: Beilstein J Org Chem. 2011 Aug 16;7:1115–23. doi: 10.3762/bjoc.7.128 (PMC3169339; doi:10.3762/bjoc.7.128)
Supplement: File 1 — Experimental section; copies of 1H and 13C NMR spectra for new compounds. [file Beilstein_J_Org_Chem-07-1115-s001.pdf]

Supporting Information

for

Amine-linked diglycosides: Synthesis facilitated by the enhanced reactivity of allylic electrophiles, and glycosidase inhibition assays

Ian Cumpstey[1,2,]*, Jens Frigell[1], Elias Pershagen[1], Tashfeen Akhtar[1], Elena Moreno-Clavijo[3], Inmaculada Robina[3], Dominic S. Alonzi[4] and Terry D. Butters[4]

Address: [1]Department of Organic Chemistry, The Arrhenius Laboratory, Stockholm University, 106 91 Stockholm, Sweden, [2]Institut de Chimie des Substances Naturelles, Centre National de la Recherche Scientifique, 91198 Gif-sur-Yvette CEDEX, France, [3]Department of Organic Chemistry, Faculty of Chemistry, University of Seville, Prof. García González, 1, 41012 Seville, Spain and [4]Glycobiology Institute, Department of Biochemistry, Oxford University, South Parks Road, Oxford, OX1 3QU, England

Email: Ian Cumpstey - ian.cumpstey@sjc.oxon.org

* Corresponding author

Experimental section; copies of ^{1}H and ^{13}C NMR spectra for new compounds

1. Experimental section
2. Copies of ^{1}H and ^{13}C NMR spectra for new compounds

1. Experimental section

General procedures

Proton nuclear magnetic resonance (^{1}H) spectra were recorded on Bruker Avance II 500 (500 MHz), Bruker Avance II 400 (400 MHz) or Varian Mercury 400 (400 MHz) spectrometers; multiplicities are quoted as singlet (s), doublet (d), doublet of doublets (dd), doublet of doublet of doublets (ddd), triplet (t), apparent triplet (at), doublet of apparent triplets (dat), quartet (q), and multiplet (m). Carbon nuclear magnetic resonance (^{13}C) spectra were recorded on Bruker Avance II 500 (125 MHz), Bruker Avance II 400 (100 MHz) or Varian Mercury 400 (100 MHz) spectrometers. ^{1}H and ^{13}C spectra and ^{13}C multiplicities were assigned using COSY, HSQC, DEPT experiments. All chemical shifts are quoted on the δ-scale in parts per million (ppm). Residual

solvent signals or TMS were used as an internal reference. Low- and high-resolution (HRMS) electrospray (ESI) mass spectra were recorded using a Bruker Microtof instrument. Infra-red spectra were recorded on a Perkin-Elmer Spectrum One FT-IR spectrometer using the thin film method on NaCl plates. Optical rotations were measured on a Perkin-Elmer 241 polarimeter with a path length of 1 dm; concentrations are given in g/100mL. Thin layer chromatography (TLC) was carried out on Merck Kieselgel sheets, pre-coated with $60F_{254}$ silica. Plates were visualised with UV light and developed using 10% sulfuric acid, or an ammonium molybdate (10% w/v) and cerium(IV) sulfate (2% w/v) solution in 10% sulfuric acid. Flash column chromatography was carried out on silica gel (35–70 µm, Grace). The palladium catalyst was from Strem and used as supplied. Dichloromethane was distilled from calcium hydride. THF was dried using a solvent purifier by VAC. DMF and acetonitrile were bought anhydrous and used as supplied. Reactions performed under an atmosphere of nitrogen or argon were maintained by an inflated balloon.

Ethyl 4,6-di-*O*-acetyl-2,3-dideoxy-*threo*-hex-2-enopyranoside (4)

Galactal **3** (879 mg, 3.23 mmol) was dissolved in acetonitrile (30 mL) under Ar. EtOH (0.37 mL, 6.5 mmol) was added, and the reaction mixture was cooled in an ice-bath. Phosphomolybdic acid (62 mg, 0.032 mmol) was added slowly. The ice-bath was removed and the reaction mixture was stirred at RT for 2 h. After this time, TLC (Pentane–EtOAc, 2:1) indicated the formation of one major (R_f 0.8) and two minor (R_f 0.7, 0.5) products and the consumption of starting material (R_f 0.7). The reaction mixture was diluted with CH_2Cl_2 (50 mL) and washed with $NaHCO_3$ (sat. aq. 2 × 50 mL), H_2O (50 mL) and brine (50 mL). Each aqueous phase was extracted with CH_2Cl_2 (25 mL). The combined organic phases were dried over Na_2SO_4, filtered and concentrated to dryness to give a brown oil (863 mg). The crude product was purified by column chromatography (Pentane–EtOAc, 6:1 → 4:1) to give the Ferrier product **4** (529 mg, 63%) as an oil, (α:β, 8:1); ^{1}H NMR: (selected for α anomer) δ = 1.25 (t, *J* = 7.2 Hz, 3 H, $OCH_2C\underline{H}_3$), 2.07, 2.08 (2 × s, 6 H, 2 × C(O)CH3), 3.59, 3.84 (dq, J_{gem} 9.6 Hz, *J* 7.2 Hz, 2 H, $OC\underline{H}_2CH_3$), 4.22–4.24 (m, 2 H, 6’-H, 6-H), 4.37 (m, 1 H, 5-H), 5.02 (dd, $J_{3,4}$ 5.2 Hz, $J_{4,5}$ 2.4 Hz, 1 H, 4-H), 5.08 (d, $J_{1,2}$ 2.8 Hz, 1 H, 1-H), 6.03 (dd, $J_{2,3}$ 10.0 Hz, $J_{1,2}$ 2.8 Hz, 1 H, 2-H), 6.11 (ddd, $J_{2,3}$ 10.0, $J_{3,4}$ 5.2 Hz, *J* 0.8 Hz, 1 H, 3-H);

as well as ethyl 2-deoxy-3,4,6-tri-*O*-acetyl-α-D-*lyxo*-hexopyranoside (169 mg, 16%); δ = 1.22 (t, *J* 7.2 Hz, 3 H, OCH_2CH_3), 1.86, 2.10 (2 × m, 2 H, 2’-H, 2-H), 1.98, 2.05, 2.13 (3 × s, 9 H, 3 × $COCH_3$), 3.48, 3.69 (2 × dq, J_{gem} 9.6 Hz, *J* 7.2 Hz, 2 H, OCH_2CH_3), 4.08–4.09 (2H, m, 6’-H, 6-H), 4.15 (m, 1 H, 5-H), 5.02 (d, $J_{1,2}$ 3.6 Hz, 1 H, 1-H), 5.28–5.33 (m, 2 H, 3-H, 4-H);

and ethyl 2-deoxy-3,4,6-tri-*O*-acetyl-β-D-*lyxo*-hexopyranoside (42 mg, 4%); δ = 1.25 (t, *J* 7.2 Hz, 3 H, OCH_2CH_3), 1.94–1.98 (m, 2 H, 2'-H, 2-H), 2.00, 2.04, 2.13 (3 × s, 9 H, 3 × $COCH_3$), 3.79 (dt, *J* 6.8 Hz, *J* 1.2 Hz, 1 H, 5-H), 3.48, 3.69 (2 × dq, J_{gem} 9.6 Hz, *J* 7.2 Hz, 2 H, OCH_2CH_3), 4.09–4.20 (2H, m, 6'-H, 6-H), 4.57 (m, 1 H, 1-H), 5.00 (m, 1 H, 3-H), 5.27 (m, 1 H, 4-H).

Ethyl 6-*O*-(*tert*-butyldimethylsilyl)-2,3-dideoxy-α-D-*threo*-hex-2-enopyranoside (2)

Compound **4** (470 mg, 1.82 mmol) was dissolved in MeOH (12 mL). Na (132 mg, 5.46 mmol) was added to MeOH (10 mL), and the resulting solution was added dropwise to the carbohydrate solution. The reaction mixture was stirred for 14 h, after which time TLC (EtOAc) indicated the complete consumption of starting material (R_f 0.9) and the formation of a major product (R_f 0.4). The reaction mixture was neutralized by the addition of NH_4Cl (292 mg, 5.46 mmol) and concentrated to dryness. The crude product was purified by column chromatography (EtOAc) to give the diol (277 mg, 87%) as a white solid; ^{1}H NMR (CD_3OD): δ = 1.22 (t, *J* = 7.2 Hz, 3 H, $OCH_2C\underline{H}_3$), 3.71–3.77 (m, 2 H, 6'-H, 6-H), 3.79 (dd, $J_{3,4}$ = 5.6 Hz, $J_{4,5}$ = 2.4 Hz, 1 H, 4-H), 3.55, 3.89 (2 × dq, J_{gem} = 9.6 Hz, *J* = 7.2 Hz, 2 H, $OC\underline{H}_2CH_3$), 3.98 (ddd, *J* = 7.2 Hz, *J* = 5.2 Hz, $J_{4,5}$ = 2.4 Hz, 1 H, 5-H), 5.03 (d, $J_{1,2}$ = 2.8 Hz, 1 H, 1-H), 5.88 (dd, $J_{2,3}$ = 10.0 Hz, $J_{1,2}$ = 2.8 Hz, 1 H, 2-H), 6.08 (ddd, $J_{2,3}$ = 10.0 Hz, $J_{3,4}$ = 5.6 Hz, *J* = 0.4 Hz, 1 H, 3-H).

The diol (267 mg, 1.53 mmol) was dissolved in DMF (10 mL). Imidazole (209 mg, 3.07 mmol) was added and the reaction mixture was cooled to 0 °C in an ice-bath. TBDMSCl (254 mg, 1.68 mmol) was added and the reaction mixture was slowly warmed to RT. After 25 h, TLC (Pentane–EtOAc, 4:1) showed the almost complete consumption of starting material (R_f ~ 0) and the formation of a major product (R_f 0.4). The reaction was quenched by addition of H_2O (50 mL) and transferred to a separatory funnel. The aqueous phase was extracted with Et_2O (3 × 50 mL), and the combined organic phases were dried (Na_2SO_4), filtered and concentrated to dryness to give a colourless oil (1.17 g). The crude product was purified by column chromatography (Pentane–EtOAc, 4:1) to give the silyl ether **2** (302 mg, 68%) as a colourless oil; ^{1}H NMR: δ = 0.09, 0.09 (2 × s, 6 H, $Si(CH_3)_2$), 0.91 (s, 9 H, $SiC(CH_3)_3$), 1.23 (t, *J* = 6.8 Hz, 3 H, $OCH_2C\underline{H}_3$), 3.54 (dq, J_{gem} = 9.6 Hz, *J* = 6.8 Hz, 1 H, $OC\underline{H}H'CH_3$), 3.82–3.91 (m, 4 H, 5-H, 6-H, 6'-H, $OCH\underline{H}'CH_3$), 4.08 (dt, *J* = 6.4 Hz, *J* = 2.4 Hz, 1 H, 4-H), 5.03 (d, $J_{1,2}$ = 2.8 Hz, 1 H, 1-H), 5.91 (dd, $J_{2,3}$ = 10.0 Hz, $J_{1,2}$ = 2.8 Hz, 1 H, 2-H), 6.16 (ddd, $J_{2,3}$ = 10.0 Hz, $J_{3,4}$ = 4.4 Hz, *J* = 0.8 Hz, 1 H, 3-H); ^{13}C NMR: δ = –5.25, –5.29 (2 × q, 2 × $SiCH_3$), 15.4 (q, $OCH_2\underline{C}H_3$), 18.4 (s, $\underline{C}(CH_3)_3$), 25.9 (q, $C(\underline{C}H_3)_3$), 62.1 (d, C-5), 63.0 (t, C-6), 63.7 (t, $O\underline{C}H_2CH_3$), 70.7 (d, C-4), 94.2 (d, C-1), 128.8 (d, C-2), 129.4 (d, C-3).

Methyl 6-amino-2,3,4-tri-*O*-benzyl-6-deoxy-6-*N*-(2-nitrobenzenesulfonyl)-α-D-mannopyranoside (9)

Amine **8** (985 mg, 2.13 mmol) was dissolved in CH_2Cl_2 (30 mL) at RT under N_2, and Et_3N (443 µL, 3.19 mmol), NsCl (567 mg, 2.55 mmol), and DMAP (26 mg, 0.21 mmol) were added. After 15 min, TLC (toluene–EtOAc, 1:1) showed the complete consumption of starting material (R_f 0.1) and the formation of a single product (R_f 0.9). The mixture was concentrated in vacuo, and the crude product was purified by column chromatography (pentane–EtOAc, 5:1 → 1:1) to give nosylamide **9** (1.22 g, 88%) as an orange oil; IR (film): 3366 (N–H), 1540 (N–O), 1363 cm^{-1}; ^1H NMR (500 MHz, $CDCl_3$): δ = 3.16–3.22 (m, 4 H, 6-H, OCH_3), 3.47 (ddd, $J_{5,6'}$ = 2.7 Hz, $J_{NH,6'}$ = 7.0 Hz, $J_{6,6'}$ = 9.8 Hz, 1 H, 6'-H), 3.63 (m, 1 H, 5-H), 3.74–3.79 (m, 2 H, 2-H, 4-H), 3.84 (dd, $J_{2,3}$ = 2.8 Hz, $J_{3,4}$ = 9.3 Hz, 1 H, 3-H), 4.55 (br s, 1 H, 1-H), 4.59 (s, 2 H, PhC$\underline{H}_2$), 4.62, 4.93 (2 × d, J = 11.1 Hz, 2 H, PhC$\underline{H}_2$), 4.64, 4.72 (2 × d, J = 12.3 Hz, 2 H, PhC$\underline{H}_2$), 5.69 (at, J = 5.9 Hz, 1 H, NH), 7.25–8.05 (m, 19 H, Ar-H); ^{13}C NMR (125 MHz, $CDCl_3$): δ = 44.9 (t, C-6), 55.1 (q, O$\underline{C}H_3$), 70.5 (d, C-5), 72.3, 73.1, 74.6, 75.2, 75.3 (3 × t, 2 × d, 3 × Ph$\underline{C}H_2$, C-2, C-4), 80.1 (d, C-3), 99.4 (d, C-1), 125.4, 127.7, 127.8, 127.9, 128.0, 128.0, 128.4, 128.6, 128.6, 131.2, 132.7, 133.5 (12 × d, Ar-CH), 134.0, 138.2, 138.4, 138.4, 148.2 (5 × s, Ar-C); MS (ESI^+): *m/z* (%) 687 (7) [M + K^+], 671 (100) [M + Na^+]; HRMS–ESI *m/z* [M + Na^+] calcd for $C_{34}H_{36}N_2O_9SNa$: 671.2034; found: 671.2024.

***N*-(methyl 2,3,4-tri-*O*-benzyl-6-deoxy-α-D-glucopyranosid-6-yl)-*N*-(ethyl 6-*O*-(*tert*-butyldimethylsilyl)-2,3,4-trideoxy-α-D-*threo*-hex-2-enopyranosid-4-yl)-2-nitrobenzenesulfonamide (10)**

Allylic alcohol **1** (50 mg, 0.17 mmol) and nosylamide **6** (113 mg, 0.17 mmol) were dissolved in THF (3 mL) and cooled to 0 °C under N_2. PPh_3 (92 mg, 0.35 mmol) was added, and after 10 min, DIAD (67 µl, 0.35 mmol). After 30 min, a white precipitate appeared. The reaction mixture was allowed to warm to RT slowly in the ice-bath. After 12 h, TLC (pentane–EtOAc, 3:1) showed the formation of a product (R_f 0.3) and the complete consumption of the alcohol (R_f 0.5) and sulfonamide (R_f 0.1) starting materials. At this time, the reaction mixture was a colourless solution. CH_2Cl_2 (ca 3 mL) was added, and the solution was concentrated in vacuo. The residue was purified by flash column chromatography (twice: pentane–EtOAc, 3:1, followed by CH_2Cl_2–Et_2O, 9:1), to give the pseudodisaccharide **10** (145 mg, 91%) as a colourless oil; $[\alpha]_D^{22}$ –22.4 (*c* 1.0, $CHCl_3$); IR (film): 1545 (NO_2), 1370 (SO_2) cm^{-1}; ^1H NMR (500 MHz, $CDCl_3$): δ = 0.06 (s, 6 H, $Si(CH_3)_2$), 0.90 (s, 9 H, $C(CH_3)_3$), 1.20 (t, J = 7.0 Hz, 3 H, $OCH_2C\underline{H}_3$), 3.06 (at, J = 9.2 Hz, 1 H, 4-H^{II}), 3.12 (s, 3 H, OCH_3), 3.42–3.47 (m, 2 H, 2-H^{II}, OC$\underline{H}$H'CH_3), 3.58 (dd, $J_{5,6}$ = 9.7 Hz, $J_{6,6'}$ = 15.4 Hz, 1 H, 6-H^{II}), 3.73–3.78 (m, 2 H, 6-H^{I}, 5-H^{II}), 3.83–3.94 (m, 4 H, 3-H^{II}, 6'-H^{II}, 6'-H^{I}, OCH$\underline{H}$'CH_3), 4.15

(m, 1 H, 5-H^{I}), 4.28 (m, 1 H, 4-H^{I}), 4.45 (d, $J_{1,2}$ = 3.5 Hz, 1 H, 1-H^{II}), 4.58, 4.94 (2 × d, *J* = 10.9 Hz, 2 H, PhC$\underline{H}_2$), 4.64, 4.76 (2 × d, *J* = 12.1 Hz, 2 H, PhC$\underline{H}_2$), 4.79–4.81 (m, 2 H, 1-H^{I}, PhC$\underline{H}$H'), 4.98 (d, *J* = 10.8 Hz, 1 H, PhCH$\underline{H}$'), 5.86 (s, 2 H, 2-H^{I}, 3-H^{I}), 7.31–7.38 (m, 15 H, 3 × Ph-H), 7.54–7.67 (m, 3H, Ns-H), 8.08 (m, 1 H, Ns-H); ^{13}C NMR (125 MHz, $CDCl_3$): δ = –5.2, –5.2 (2 × q, $Si(CH_3)_2$), 15.1 (q, $OCH_2\underline{C}H_3$), 18.3 (s, Si$\underline{C}(CH_3)_3$), 25.9 (q, SiC($\underline{C}H_3)_3$), 47.9 (t, C-6^{II}), 50.9 (d, C-4^{I}), 55.7 (q, OCH_3), 63.1, 63.2 (2 × t, C-6^{I}, O$\underline{C}H_2CH_3$), 69.1 (d, C-5^{II}), 71.9 (d, C-5^{I}), 73.2, 75.4, 75.7 (3 × t, 3 × PhC$\underline{H}_2$), 80.0 (d, C-2^{II}), 80.4 (d, C-4^{II}), 81.9 (d, C-3^{II}), 93.5 (d, C-1^{I}), 97.9 (d, C-1^{II}), 124.0 (d, Ns-CH), 127.0, 130.5 (2 × d, C-2^{I}, C-3^{I}), 127.7, 127.9, 127.9, 128.0, 128.0, 128.2, 128.4, 128.5, 128.5 (9 × d, Ar-CH), 131.8, 132.4, 133.6 (3 × d, Ns-CH), 134.6, 148.0 (2 × s, Ns-C), 138.2, 138.7 (2 × s, Ph-C); MS (ESI^+): *m/z* (%) 941 (100) [M + Na^+]; HRMS–ESI *m/z* [M + Na^+] calcd for $C_{48}H_{62}O_{12}N_2SSiNa$: 941.3685; found: 941.3706.

N-(methyl 2,3,4-tri-O-benzyl-6-deoxy-α-D-glucopyranosid-6-yl)-N-(ethyl 6-O-(tert-butyldimethylsilyl)-2,3,4-trideoxy-α-D-*threo*-hex-2-enopyranosid-4-yl)-trifluoromethanesulfonamide (11)

Allylic alcohol **1** (50 mg, 0.17 mmol) and triflamide **7** (103 mg, 0.17 mmol) were dissolved in THF (3 mL) and cooled to 0 °C under N_2. PPh_3 (92 mg, 0.35 mmol) was added, and after 10 min, DIAD (68 μl, 0.35 mmol). Within 2 min, the yellow colour had disappeared. After 5 min, a white precipitate had appeared. The reaction mixture was removed from the ice-bath after 10 min and stirred at RT. After 2 h at RT, TLC (pentane–EtOAc, 5:1) showed the formation of a product (R_f 0.4) and the presence of the remaining alcohol (R_f 0.2) and sulfonamide (R_f 0.1) starting materials. At this time, the mixture still had the appearance of a white suspension. After 24 h, TLC showed the complete conversion of both of the starting materials into product; the reaction mixture was a colourless solution. CH_2Cl_2 (ca 3 mL) was added, and the solution was concentrated in vacuo. The residue was purified by flash column chromatography (pentane–EtOAc, 9:1) to give the pseudodisaccharide **11** (132 mg, 88%) as a colourless oil; $[\alpha]_D^{22}$ –47.8 (*c* 1.0, $CHCl_3$); IR (film): 1395 (SO_2) cm^{-1}; ^{1}H NMR (500 MHz, $CDCl_3$): δ = 0.06 (s, 6 H, $Si(CH_3)_2$), 0.89 (s, 9 H, $C(CH_3)_3$), 1.18 (t, *J* = 7.1 Hz, 3 H, $OCH_2C\underline{H}_3$), 3.04 (at, *J* = 9.3 Hz, 1 H, 4-H^{II}), 3.37–3.45 (m, 2 H, 6-H^{II}, OC$\underline{H}$H'CH_3), 3.51 (s, 3 H, OCH_3), 3.51 (m (obs), 1 H, 2-H^{II}), 3.60 (d, $J_{6,6'}$ = 14.7 Hz, 1 H, 6'-H^{II}), 3.76 (dd, $J_{5,6}$ = 7.7 Hz, $J_{6,6'}$ = 11.2 Hz, 1 H, 6-H^{I}), 3.82 (m, 1 H, OCH$\underline{H}$'CH_3), 3.96 (dd, $J_{5,6'}$ = 3.3 Hz, $J_{6,6'}$ = 11.3 Hz, 1 H, 6'-H^{I}), 4.01 (at, *J* = 9.3 Hz, 1 H, 3-H^{II}), 4.04 (m, 1 H, 5-H^{I}), 4.10 (at, *J* = 9.4 Hz, 1 H, 5-H^{II}), 4.17 (m, 1 H, 4-H^{I}), 4.46, 4.94 (2 × d, *J* = 11.0 Hz, 2 H, PhC$\underline{H}_2$), 4.65–4.69 (m, 1-H^{I}, 1-H^{II}, 3 H, PhC$\underline{H}$H'), 4.78 (d, *J* = 12.2 Hz, 1 H, PhCH$\underline{H}$'), 4.82, 5.02 (2 × d, *J* = 10.8 Hz, 2 H, PhC$\underline{H}_2$), 5.70 (dd, $J_{3,4}$ = 5.5 Hz, $J_{2,3}$ = 10.0 Hz, 1 H, 3-H^{I}), 5.79 (dd, $J_{1,2}$ = 1.9 Hz, 1 H, 2-H^{I}),

7.27–7.38 (m, 15 H, Ar-H); ^{13}C NMR (125 MHz, $CDCl_3$): δ = –5.2, –5.2 (2 × q, $Si(CH_3)_2$), 15.2 (q, $OCH_2\underline{C}H_3$), 18.2 (s, $Si\underline{C}(CH_3)_3$), 25.9 (q, $SiC(\underline{C}H_3)_3$), 48.7 (t, C-6^{II}), 53.0 (d, C-4^{I}), 57.1 (q, OCH_3), 62.7 (t, C-6^{I}), 63.5 (t, $O\underline{C}H_2CH_3$), 69.3 (d, C-5^{II}), 71.1 (d, C-5^{I}), 73.3, 75.3, 75.8 (3 × t, 3 × $PhC\underline{H_2}$), 79.9 (br d, C-4^{II}), 80.1 (d, C-2^{II}), 82.0 (d, C-3^{II}), 93.4 (d, C-1^{I}), 98.6 (d, C-1^{II}), 120.3 (q, $J_{C,F}$ 324 Hz, CF_3), 124.2 (v br d, C-3^{I}), 127.8, 128.0, 128.1, 128.1, 128.4, 128.5, 128.5, 128.6 (8 × d, Ar-CH), 133.7 (v br d, C-2^{I}), 138.1, 138.3, 138.7 (3 × s, 3 × Ar-C); MS (ESI$^+$): *m/z* (%) 888 (100) [M + Na$^+$]; HRMS–ESI *m/z* [M + Na$^+$] calcd for $C_{43}H_{58}O_{10}F_3NSSiNa$: 888.3395; found: 888.3448.

N-(methyl 2,3,4-tri-O-benzyl-6-deoxy-α-D-mannopyranosid-6-yl)-N-(ethyl 6-O-(tert-butyldimethylsilyl)-2,3,4-trideoxy-α-D-threo-hex-2-enopyranosid-4-yl)-2-nitrobenzenesulfonamide (12)

Allylic alcohol **1** (153 mg, 0.527 mmol) and nosylamide **9** (401 mg, 0.619 mmol) were dissolved in THF (11 mL), and the reaction was cooled to 0 °C under nitrogen. PPh_3 (276 mg, 1.05 mmol) was added. After 10 min, DIAD (207 µL, 1.05 mmol) was added. After 30 min, the reaction mixture was allowed to reach RT. After 18 h, TLC (toluene–EtOAc, 5:1) showed only a little starting material remaining (**1** R_f 0.3, **9** R_f 0.7) and the formation of a single product (R_f 0.8). The mixture was concentrated in vacuo, and the crude product was purified by repeated column chromatography (pentane–EtOAc, 4:1) to give the pseudodisaccharide **12** (368 mg, 76%) as an oil; $[\alpha]_D^{25}$ –12.3 (*c* 1.0, $CHCl_3$); IR (film): 1545 (N–O), 1370 cm^{-1}; ^{1}H NMR (500 MHz, $CDCl_3$): δ = 0.01 (s, 6 H, $Si(CH_3)_2$), 0.86 (s, 9 H, $C(CH_3)_3$), 1.19 (t, J = 7.1 Hz, 3 H, $OCH_2C\underline{H_3}$), 3.07 (s, 3 H, OCH_3), 3.42, 3.85 (2 × m, 2 H, $OC\underline{H_2}CH_3$), 3.49 (at, J = 8.5 Hz, 1 H, 4-H^{II} or 5-H^{II}), 3.68 (m, 1 H, 2-H^{II}), 3.71–3.77 (m, 5 H, 4-H^{II} or 5-H^{II}, and 6-H^{I}, 6'-H^{I}, 3-H^{II}, 6-H^{II}), 3.91 (m, 1 H, 6'-H^{II}), 4.15 (m, 1 H, 5-H^{I}), 4.28 (at, J = 4.0 Hz, 1 H, 4-H^{I}), 4.54–4.62 (m, 5 H, 1-H^{II}, $PhC\underline{H_2}$, 2 × $PhC\underline{H}H'$), 4.69 (d, J = 12.4 Hz, 1 H, $PhCH\underline{H'}$), 4.81 (d, $J_{1,2}$ = 2.6 Hz, 1 H, 1-H^{I}), 4.92 (d, J = 10.9 Hz, 1 H, $PhCH\underline{H'}$), 5.85 (dd, $J_{1,2}$ = 1.8 Hz, $J_{2,3}$ = 10.0 Hz, 1 H, 2-H^{I}), 5.93 (dd, $J_{2,3}$ = 10.0 Hz, $J_{3,4}$ = 5.4 Hz, 1 H, 3-H^{I}), 7.26–7.38 (m, 15 H, Ar-H), 7.51 (at, J = 7.8 Hz, 1 H, Ar-H), 7.58 (d, J = 7.8 Hz, 1 H, Ar-H), 7.64 (at, J = 7.8 Hz, 1 H, Ar-H), 8.07 (d, J = 7.8 Hz, 1 H, Ar-H); ^{13}C NMR (125 MHz, $CDCl_3$): δ = –5.2 (q, $Si(CH_3)_2$), 15.2 (q, $OCH_2\underline{C}H_3$), 18.4 (s, $Si\underline{C}(CH_3)_3$), 26.0 (q, $SiC(\underline{C}H_3)_3$), 48.4 (t, C-6^{II}), 50.9 (d, C-4^{I}), 55.1 (q, $O\underline{C}H_3$), 63.1, 63.3 (2 × t, C-6^{I}, $O\underline{C}H_2CH_3$), 70.3, 77.3, 79.9 (3 × d, C-3^{II}, C-4^{II}, C-5^{II}), 71.9 (d, C-5^{I}), 72.0, 72.6, 75.2 (3 × t, 3 × $Ph\underline{C}H_2$), 75.0 (d, C-2^{II}), 93.6 (d, C-1^{I}), 98.6 (d, C-1^{II}), 124.0, 127.6, 127.7, 127.7, 127.7, 127.8, 128.2, 128.4, 128.4, 131.7, 132.5, 133.5 (12 × d, Ar-CH), 127.4 (d, C-3^{I}), 130.2 (d, C-2^{I}), 134.6, 138.5, 138.6, 138.6, 148.1 (5 × s, 5 × Ar-C); MS (ESI$^+$): *m/z* (%) 957 (5) [M + K$^+$], 941 (100) [M + Na$^+$]; HRMS–ESI *m/z* [M + Na$^+$] calcd for $C_{48}H_{62}N_2O_{12}SSiNa$: 941.3685; found: 941.3715.

_N_-(methyl 2,3,4-tri-_O_-benzyl-6-deoxy-α-D-glucopyranosid-6-yl)-_N_-(ethyl 6-_O_-(_tert_-butyldimethylsilyl)-2,3,4-trideoxy-α-D-_erythro_-hex-2-enopyranosid-4-yl)-2-nitrobenzenesulfonamide (13)

Allylic alcohol **2** (56 mg, 0.19 mmol) and nosylamide **6** (125 mg, 0.193 mmol) were dissolved in THF (3.6 mL), and the reaction mixture was cooled to 0 °C under nitrogen. PPh_3 (101 mg, 0.386 mmol) was added. After 10 min, DIAD (76 µL, 0.39 mmol) was added, and within 10 min, the reaction mixture turned from a clear, pale yellow solution to a white slurry. After 30 min, the reaction mixture was allowed to reach RT. After 3 h, TLC (toluene–EtOAc, 5:1) showed the presence of a little remaining starting material (**2** R_f 0.3, **6** R_f 0.5), along with product formation (R_f 0.8). PPh_3 (50 mg, 0.19 mmol) was added to the pale yellow reaction mixture. After 10 min, DIAD (38 µL, 0.19 mmol) was added, and again a white slurry was seen within 10 min. The reaction was allowed to reach RT and after 16 h, TLC showed that all starting material had been consumed and a single product had been formed. The reaction mixture was concentrated in vacuo, and the crude product was purified by column chromatography (toluene–EtOAc, 9:1) to give pseudodisaccharide **13** (147 mg, 91%) as a pale yellow oil; $[\alpha]_D^{25}$ +31.4 (_c_ 1.0, $CHCl_3$); IR (film): 1546 (N–O), 1372 cm^{-1}; ^{1}H NMR (400 MHz, $CDCl_3$): δ = 0.04, 0.07 (2 × s, 6 H, $Si(CH_3)_2$), 0.90 (s, 9 H, $C(CH_3)_3$), 1.48 (t, J = 7.1 Hz, 3 H, $OCH_2C\underline{H}_3$), 3.02 (dd, $J_{5,6}$ = 10.1 Hz, $J_{6,6'}$ = 15.5 Hz, 1 H, 6-H^{II}), 3.12 (at, J = 9.5 Hz, 1 H, 4-H^{II}), 3.44–3.53 (m, 5 H, 2-H^{II}, $OC\underline{H}_3$, $OC\underline{H}H'CH_3$), 3.57 (d, $J_{6,6'}$ = 15.5 Hz, 1 H, 6-$H^{II'}$), 3.66 (dd, $J_{5,6}$ = 7.2 Hz, $J_{6,6'}$ = 11.2 Hz, 1 H, 6-H^{I}), 3.83 (dq, J = 7.1 Hz, J_{gem} = 9.6 Hz, 1 H, $OCH\underline{H}'CH_3$), 3.97 (dd, $J_{5,6'}$ = 1.4 Hz, $J_{6,6'}$ = 11.2 Hz, 1 H, 6-$H^{I'}$), 4.08 (at, J = 9.2 Hz, 1 H, 3-H^{II}), 4.19 (m, 1 H, 5-H^{I}), 4.25 (atd, J = 10.0 Hz, $J_{5,6'}$ = 1.3 Hz, 1 H, 5-H^{II}), 4.42 (m, 1 H, 4-H^{I}), 4.51, 4.97 (2 × d, J = 11.5 Hz, 2 H, $PhC\underline{H}_2$), 4.57 (d, $J_{1,2}$ = 3.4 Hz, 1 H, 1-H^{II}), 4.67, 4.78 (2 × d, J = 12.2 Hz, 2 H, $PhC\underline{H}_2$), 4.80, 5.00 (2 × d, J = 11.0 Hz, 2 H, $PhC\underline{H}_2$), 4.92 (d, $J_{1,2}$ = 2.6 Hz, 1 H, 1-H^{I}), 5.41 (d, $J_{2,3}$ = 10.2 Hz, 1 H, 3-H^{I}), 5.83 (dat, J = 2.7 Hz, $J_{2,3}$ = 10.2 Hz, 1 H, 2-H^{I}), 7.14 (m, 1 H, Ar-H), 7.24–7.37 (m, 15 H, Ar-H), 7.54–7.61 (m, 2 H, Ar-H), 7.85 (m, 1 H, Ar-H); ^{13}C NMR (100 MHz, $CDCl_3$): δ = –5.2, –5.1 (2 × q, $Si(CH_3)_2$) 15.4 (q, $OCH_2\underline{C}H_3$), 18.4 (s, $Si\underline{C}(CH_3)_3$), 26.1 (q, $SiC(\underline{C}H_3)_3$), 47.9 (t, C-6^{II}), 54.1 (d, C-4^{I}), 55.6 (q, $O\underline{C}H_3$), 63.2 (t, C-6^{I}), 63.3 (t, $O\underline{C}H_2CH_3$), 69.1 (d, C-5^{I}), 69.7 (d, C-5^{II}), 73.3, 74.5, 75.7 (3 × t, 3 × $Ph\underline{C}H_2$), 79.8 (d, C-2^{II}), 80.5 (d, C-4^{II}), 81.7 (d, C-3^{II}), 93.0 (d, C-1^{I}), 97.9 (d, C-1^{II}), 124.2, 127.7, 128.0, 128.1, 128.2, 128.3, 128.5, 128.6, 131.9, 132.6, 133.7 (11 × d, Ar-CH), 129.6 (d, C-3^{I}), 130.2 (d, C-2^{I}) 131.4, 138.2, 138.4, 138.8, 148.1 (5 × s, 5 × Ar-C); MS (ESI^+): _m/z_ (%) 957 (5) [M + K^+], 941 (100) [M + Na^+]; HRMS–ESI _m/z_ [M + Na^+] calcd for $C_{48}H_{62}N_2O_{12}SSiNa$: 941.3685; found: 941.3722.

N-(methyl 2,3,4-tri-O-benzyl-6-deoxy-α-D-glucopyranosid-6-yl)-N-(ethyl 6-O-(tert-butyldimethylsilyl)-2,3,4-trideoxy-α-D-erythro-hex-2-enopyranosid-4-yl)-trifluoromethanesulfonamide (14)

Allylic alcohol **2** (70 mg, 0.24 mmol) and triflamide **7** (143 mg, 0.241 mmol) were dissolved in THF (4.3 mL), and the reaction was cooled to 0 °C under nitrogen. PPh_3 (126 mg, 0.482 mmol) was added to the mixture. After 10 min, DIAD (95 µL, 0.48 mmol) was added, and the reaction mixture turned from a clear, pale yellow solution to a white slurry within 10 min. After 30 min, the reaction mixture was allowed to reach RT. After 16 h, TLC (toluene–EtOAc, 5:1) showed the presence of remaining starting material (**2** R_f 0.4, **7** R_f 0.4), and product formation (R_f 0.8). PPh_3 (68 mg, 0.24 mmol) was added, and the pale yellow mixture was cooled to 0 °C. After 10 min, DIAD (47 µL, 0.24 mmol) was added, and again a white slurry was seen within 10 min. The reaction was allowed to reach RT. After a further 4 h, TLC showed that all starting material had been consumed and a single product had been formed. The reaction mixture was concentrated in vacuo, and the crude product was purified by column chromatography (pentane–EtOAc, 6:1) to give pseudodisaccharide **14** (189 mg, 91%) as a pale yellow oil; ^{1}H NMR (400 MHz, $CDCl_3$): δ = 0.04, 0.06 (2 × s, 6 H, $Si(CH_3)_2$), 0.89 (s, 9 H, $C(CH_3)_3$), 1.16 (t, J = 7.1 Hz, 3 H, $OCH_2C\underline{H}_3$), 2.91 (m, 1 H, 6-H^{II}), 3.08 (at, J = 9.3 Hz, 1 H, 4-H^{II}), 3.42–3.53 (m, 5 H, 2-H^{II}, $OC\underline{H}H'CH_3$, $OC\underline{H}_3$), 3.63–3.72 (m, 2 H, 6-H^{I}, 6'-H^{II}), 3.80 (dq, J = 7.1 Hz, J_{gem} = 9.6 Hz, 1 H, $OCH\underline{H}'CH_3$), 3.91 (d, $J_{6,6'}$ = 11.0 Hz, 1 H, 6'-H^{I}), 4.04 (at, J = 9.2 Hz, 1 H, 3-H^{II}) 4.08 (m, 1 H, 5-H^{II}), 4.16 (m, 1 H, 5-H^{I}), 4.35 (m, 1 H, 4-H^{I}), 4.55, 4.89 (2 × d, J = 11.6 Hz, 2 H, $PhC\underline{H}_2$), 4.61 (d, $J_{1,2}$ = 3.4 Hz, 1 H, 1-H^{II}), 4.67, 4.78 (2 × d, J = 12.1 Hz, 2 H, $PhC\underline{H}_2$), 4.80, 4.99 (2 × d, J = 10.9 Hz, 2 H, $PhC\underline{H}_2$), 4.92 (d, $J_{1,2}$ = 2.7 Hz, 1 H, 1-H^{I}), 5.55, 5.93 (2 × m, 2 H, 2-H^{I}, 3-H^{I}), 7.25–7.34 (m, 15 H, Ar-H); ^{13}C NMR (100 MHz, $CDCl_3$): δ = –5.2, –5.1 (2 × q, $Si(CH_3)_2$), 15.4 (q, $OCH_2\underline{C}H_3$), 18.4 (s, $\underline{C}(CH_3)_3$), 26.0 (q, $C(\underline{C}H_3)_3$), 49.2 (t, C-6^{II}), 55.5 (d, C-4^{I}), 55.7 (q, $O\underline{C}H_3$), 62.9 (t, C-6^{I}), 63.6 (t, $O\underline{C}H_2CH_3$), 68.6 (d, C-5^{I}), 69.5 (d, C-5^{II}), 73.3, 74.6, 75.9 (3 × t, 3 × $Ph\underline{C}H_2$), 79.8, 80.0 (2 × d, C-2^{II}, C-4^{II}), 81.7 (d, C-3^{II}), 93.0 (d, C-1^{I}), 98.1 (d, C-1^{II}), 127.8, 127.9, 128.1, 128.1, 128.2, 128.4, 128.4, 128.5, 128.6, 128.6, 131.5 (11 × d, C-2^{I}, C-3^{I}, Ar-C), 138.0, 138.1, 138.7 (3 × s, 3 × Ar-C); MS (ESI^+): *m/z* (%) 904 (5) [M + K^+], 888 (100) [M + Na^+]; HRMS–ESI *m/z* [M + Na^+] calcd for $C_{43}H_{58}NO_{10}F_3SSiNa$: 888.3395; found: 888.3414.

_N_-(methyl 2,3,4-tri-_O_-benzyl-6-deoxy-α-D-mannopyranosid-6-yl)-_N_-(ethyl 6-_O_-(_tert_-butyldimethylsilyl)-2,3,4-trideoxy-α-D-_erythro_-hex-2-enopyranosid-4-yl)-2-nitrobenzenesulfonamide (15)

Allylic alcohol **2** (101 mg, 0.348 mmol) and nosylamide **9** (248 mg, 0.383 mmol) were dissolved in THF (7 mL), and the reaction was cooled to 0 °C under nitrogen. PPh_3 (182 mg, 0.696 mmol) was added. After 10 min, DIAD (136 µL, 0.696 mmol) was added. After 30 min, the reaction mixture was allowed to reach RT. After 18 h, TLC (toluene–EtOAc, 6:1) showed only a little starting material (**2** R_f 0.3, **9** R_f 0.7) remaining and the formation of a single product (R_f 0.75). The reaction mixture was concentrated in vacuo, and the crude product was purified by repeated column chromatography (pentane–EtOAc, 4:1) to give the pseudodisaccharide **15** (227 mg, 71%) as an oil; $[\alpha]_D^{25}$ +46.1 (*c* 1.0, $CHCl_3$); IR (film): 1546 (N–O), 1372 cm^{-1}; ^1H NMR (500 MHz, $CDCl_3$): δ = 0.08, 0.11 (2 × s, 6 H, $Si(CH_3)_2$), 0.94 (s, 9 H, $C(C\underline{H}_3)_3$), 1.19 (t, J = 7.1 Hz, 3 H, $OCH_2C\underline{H}_3$), 3.22 (dd, $J_{5,6}$ = 10.2 Hz, $J_{6,6'}$ = 15.3 Hz, 1 H, 6-H^{II}), 3.39 (s, 3 H, OCH_3), 3.51, 3.88 (2 × dq, J = 7.1 Hz, J_{gem} = 9.4 Hz, 2 H, $OC\underline{H}_2CH_3$), 3.61–3.68 (m, 2 H, 4-H^{II}, 6’-H^{II}), 3.74 (dd, $J_{5,6}$ = 7.0 Hz, $J_{6,6'}$ = 11.2 Hz, 1 H, 6-H^{I}), 3.78 (m, 1 H, 2-H^{II}), 3.97 (dd, $J_{2,3}$ = 3.0 Hz, $J_{3,4}$ = 9.3 Hz, 1 H, 3-H^{II}), 4.06 (d, $J_{6,6'}$ = 11.0 Hz, 1 H, 6’-H^{I}), 4.24 (at, J = 9.8 Hz, 1 H, 5-H^{II}), 4.32 (at, J = 8.2 Hz, 1 H, 5-H^{I}), 4.49 (d, $J_{4,5}$ = 9.8 Hz, 1 H, 4-H^{I}), 4.55, 5.05 (2 × d, J = 11.5 Hz, 2 H, $PhC\underline{H}_2$), 4.59 (m, 2 H, $PhC\underline{H}_2$), 4.69–4.71 (m, 3 H, 1-H^{II}, $PhC\underline{H}_2$), 4.95 (m, 1 H, 1-H^{I}), 5.42 (d, $J_{2,3}$ = 10.2 Hz, 1 H, 2-H^{I} or 3-H^{I}), 5.86 (dat, J = 2.6 Hz, $J_{2,3}$ = 10.2 Hz, 1 H, 2-H^{I} or 3-H^{I}), 7.11 (m, 1 H, Ar-H), 7.26–7.37 (m, 15 H, Ar-H), 7.54–7.60 (m, 2 H, Ar-H), 7.86 (m, 1 H, Ar-H); ^{13}C NMR (125 MHz, $CDCl_3$): δ = –5.1, –5.0 (2 × q, $Si(CH_3)_2$), 15.3 (q, $OCH_2\underline{C}H_3$), 18.4 (s, $Si\underline{C}(CH_3)_3$), 26.1 (q, $SiC(\underline{C}H_3)_3$), 47.9 (t, C-6^{II}), 54.2 (d, C-4^{I}), 55.0 (q, $O\underline{C}H_3$), 63.3, 63.4 (2 × t, C-6^{I}, $O\underline{C}H_2CH_3$), 69.1 (d, C-5^{I}), 70.7 (d, C-5^{II}), 72.0, 72.5, 74.7 (3 × t, 3 × $Ph\underline{C}H_2$), 74.5 (d, C-2^{II}), 77.2 (d, C-4^{II}), 80.1 (d, C-3^{II}), 93.0 (d, C-1^{I}), 98.6 (d, C-1^{II}), 124.1, 127.5, 127.6, 127.7, 127.7, 127.8, 127.9, 128.4, 128.5, 129.5, 130.2, 131.3, 132.4, 133.6 (14 × d, C-2^{I}, C-3^{I}, Ar-$\underline{C}$H), 131.9, 138.4, 138.4, 138.9, 148.1 (5 × s, 5 × Ar-$\underline{C}$); MS (ESI^+): *m/z* (%) 957 (2) $[M + K^+]$, 941 (100) $[M + Na^+]$; HRMS–ESI *m/z* $[M + Na^+]$ calcd for $C_{48}H_{62}N_2O_{12}SSiNa$: 941.3685; found: 941.3708.

_N_-(methyl 2,3,4-tri-_O_-benzyl-6-deoxy-α-D-glucopyranosid-6-yl)-_N_-(ethyl 6-_O_-(_tert_-butyldimethylsilyl)-4-deoxy-α-D-mannopyranosid-4-yl)-2-nitrobenzenesulfonamide (16)

Alkene **13** (154 mg, 0.167 mmol) was dissolved in acetone/H_2O [4:1] (1.2 mL) at RT. AcOH (10 µL, 0.18 mmol), then $K_2OsO_4 \cdot 2H_2O$ (2 mg, 5 µmol) and then NMO (50% aq., 223 µL, 0.951 mmol) were added. The reaction mixture slowly changed colour from yellow to black. TLC (toluene–EtOAc, 4:3) showed a slow but steady conversion of the starting material (R_f 0.9) to a

single product (R_f 0.3). After 72 h, the reaction was quenched by $NaHSO_3$ (satd. aq., 0.5 mL) and the mixture was left to stir for 30 min. Brine (5 mL) was added, and the mixture was extracted with EtOAc (3 × 10 mL). The organic phase was dried ($MgSO_4$), filtered and concentrated in vacuo. The crude product was purified by column chromatography (toluene–EtOAc, 5:1 → 4:3) to give diol **16** (140 mg, 88%) as a slightly yellow oil; ^{1}H NMR (400 MHz, $CDCl_3$): δ = 0.01, 0.02 (2 × s, 6 H, Si(CH_3)$_2$), 0.86 (s, 9 H, C(CH_3)$_3$), 1.11 (t, J = 6.8 Hz, 3 H, OCH$_2$C$\underline{H}_3$), 3.09–3.14 (m, 2 H, 4-H^{II}, 6-H^{II}), 3.41–3.52 (m, 6 H, 6-H^{I}, 2-H^{II}, OC$\underline{H}$H'CH$_3$, OCH$_3$), 3.63–3.73 (m, 2 H, 6'-H^{II}, OCH$\underline{H}$'CH$_3$), 3.80–3.93 (m, 5 H, 2-H^{I}, 3-H^{I}, 4-H^{I}, 5-H^{I}, 6'-H^{I}), 4.10 (at, J = 8.4 Hz, 1 H, 3-H^{II}), 4.26 (m, 1 H, 5-H^{II}), 4.54 (d, $J_{1,2}$ = 3.2 Hz, 1 H, 1-H^{II}), 4.57 (d, J = 11.5 Hz, 1 H, PhC$\underline{H}$H'), 4.66 (d, J = 12.1 Hz, 1 H, PhC$\underline{H}$H'), 4.76–4.81 (m, 3 H, 1-H^{I}, PhC$\underline{H}$H', PhCH$\underline{H}$'), 4.99–5.04 (m, 2 H, 2 × PhCH$\underline{H}$'), 7.13–7.80 (m, 19 H, Ar-H); ^{13}C NMR (100 MHz, $CDCl_3$): δ = –5.2, –5.0 (2 × q, Si(CH_3)$_2$), 15.0 (q, OCH$_2\underline{C}$H$_3$), 18.3 (s, Si$\underline{C}$(CH_3)$_3$), 26.0 (q, SiC($\underline{C}$H$_3$)$_3$), 49.9 (t, C-6^{II}), 56.2 (q, OCH$_3$), 57.3 (d, C-4^{I}), 62.8 (t, O$\underline{C}$H$_2$CH$_3$), 63.5 (t, C-6^{I}), 66.4, 70.9, 71.6 (3 × d, C-2^{I}, C-3^{I}, C-5^{I}), 70.1 (d, C-5^{II}), 73.4, 74.5, 75.8 (3 × t, 3 × Ph$\underline{C}$H$_2$), 80.1 (d, C-2^{II}), 80.5 (d, C-4^{II}), 81.5 (d, C-3^{II}), 98.3 (d, C-1^{II}), 98.5 (d, C-1^{I}), 123.8, 127.6, 127.8, 128.1, 128.2, 128.6, 128.6, 131.4, 133.6 (9 × d, Ar-CH), 131.9, 138.2, 138.4, 138.6, 147.8 (5 × s, Ar-C); MS (ESI$^+$): m/z (%) 1927 (7) [2M + Na$^+$], 975 (100) [M + Na$^+$]; HRMS–ESI m/z [M + Na$^+$] calcd for $C_{48}H_{64}N_2O_{14}SSiNa$: 975.3740; found: 975.3734.

***N*-(methyl 2,3,4-tri-*O*-benzyl-6-deoxy-α-D-mannopyranosid-6-yl)-*N*-(ethyl 6-*O*-(*tert*-butyldimethylsilyl)-4-deoxy-α-D-mannopyranosid-4-yl)-2-nitrobenzenesulfonamide (17)**

Alkene **15** (85 mg, 0.093 mmol) was dissolved in acetone/H_2O [4:1] (1 mL). AcOH (5 µL, 0.09 mmol), then $K_2OsO_4 \cdot 2H_2O$ (7 mg, 19 µmol), and then NMO (43 mg, 0.37 mmol) were added. The reaction mixture slowly changed colour from yellow to black. TLC (toluene–EtOAc, 6:1) showed a slow but steady conversion of the starting material (R_f 0.7) to a single product (R_f 0.1). After 6 days, the reaction was quenched by $NaHSO_3$ (satd. aq., 0.5 mL), and the mixture was left to stir for 20 min. Brine (5 mL) was added, and the mixture was extracted with EtOAc (3 × 10 mL). The combined organic extracts were dried ($MgSO_4$), filtered and concentrated in vacuo. The crude product was purified by column chromatography (toluene–EtOAc, 5:1 → 1:1) to give diol **17** (65 mg, 73%) as an oil; ^{1}H NMR (500 MHz, $CDCl_3$): δ = 0.04, 0.06 (2 × s, 6 H, Si(CH_3)$_2$), 0.89 (s, 9 H, C(C$\underline{H}_3$)$_3$), 1.12 (t, J = 7.0 Hz, 3 H, OCH$_2$C$\underline{H}_3$), 2.23 (br s, 1 H, OH), 2.86 (d, J = 9.5 Hz, 1 H, OH), 3.26 (dd, $J_{5,6}$ = 9.9 Hz, $J_{6,6'}$ = 15.8 Hz, 1 H, 6-H^{II}), 3.35 (s, 3 H, OCH$_3$), 3.45 (dq, J = 7.1 Hz, J_{gem} = 9.7 Hz, 1 H, OC$\underline{H}$H'CH$_3$), 3.59–3.69 (m, 2 H, 6-H^{I}, 4-H^{II}), 3.71–3.78 (m, 3 H, 2-H^{y}, 6'-H^{II}, OCH$\underline{H}$'CH$_3$), 3.86–4.04 (m, 6 H, 2-H^{x}, 3-H^{I}, 4-H^{I}, 5-H^{I}, 6'-H^{I}, 3-H^{II}), 4.26 (at, J = 9.4 Hz, 1 H, 5-H^{II}), 4.58–4.72 (m, 6 H, 1-H^{y}, PhC$\underline{H}$H', 2 × PhC$\underline{H}_2$), 4.78 (s, 1 H, 1-H^{x}), 5.10 (d, J = 11.4 Hz, 1 H,

PhCH$\underline{H}$'), 7.08–7.81 (m, 19 H, Ar-H); ^{13}C NMR (125 MHz, $CDCl_3$): δ = –5.1, –4.9 (2 × q, $Si(CH_3)_2$), 14.9 (q, $OCH_2\underline{C}H_3$), 18.4 (s, Si$\underline{C}$(CH_3)$_3$), 26.0 (q, SiC($\underline{C}H_3$)$_3$), 47.1 (t, C-6^{II}), 55.5 (q, O$\underline{C}H_3$), 57.5 (d, C-4^{I}), 62.7 (t, O$\underline{C}H_2CH_3$), 63.7 (t, C-6^{I}), 66.4, 71.0, 71.6, 79.8 (4 × d, C-2^{x}, C-3^{I}, C-5^{I}, C-3^{II}), 71.1 (d, C-5^{II}), 72.0, 72.7, 74.6 (3 × t, 3 × Ph$\underline{C}H_2$), 74.6 (d, C-2^{y}), 77.2 (d, 4-H^{II}), 98.5 (d, C-1^{x}), 98.8 (d, C-1^{y}), 123.6, 127.5, 127.6, 127.8, 127.8, 128.4, 128.5, 128.5, 129.2, 131.3, 132.1, 133.5 (12 × d, Ar-$\underline{C}$H), 131.8, 138.4, 138.9, 147.8 (4 × s, Ar-C); MS (ESI^{+}): *m/z* (%) 991 (2) [M + K^{+}], 975 (100) [M + Na^{+}]; HRMS–ESI *m/z* [M + Na^{+}] calcd for $C_{48}H_{64}N_2O_{14}SSiNa$: 975.3740; found: 975.3770.

***N*-(methyl 2,3,4-tri-*O*-benzyl-6-deoxy-α-D-glucopyranosid-6-yl)-*N*-(ethyl 2,3-di-*O*-acetyl-6-*O*-(*tert*-butyldimethylsilyl)-4-deoxy-α-D-mannopyranosid-4-yl)-2-nitrobenzenesulfonamide (18)**

Diol **16** (65 mg, 0.068 mmol) was dissolved in Ac_2O/pyridine [1:1] (6 mL) at RT, and left to stir for 24 h. After this time, TLC (toluene–EtOAc, 5:1) showed the presence of a single product (R_f 0.5), and the reaction was concentrated in vacuo. The crude product was purified by column chromatography (toluene–EtOAc, 5:1) to give diacetate 1**8** (59 mg, 83%) as an oil; $[\alpha]_D^{25}$ +43.2 (*c* 1.0, $CHCl_3$); IR (film): 1749 (C=O), 1548 (N–O), 1371 cm^{-1}; ^{1}H NMR (400 MHz, $CDCl_3$): δ = 0.05, 0.07 (2 × s, 6 H, $Si(CH_3)_2$), 0.90 (s, 9 H, $C(CH_3)_3$), 1.14 (t, *J* = 7.0 Hz, 3 H, $OCH_2C\underline{H}_3$), 1.20, 2.16 (2 × s, 6 H, 2 × C(O)CH_3), 2.84 (dd, $J_{5,6}$ = 9.9 Hz, $J_{6,6'}$ = 15.8 Hz, 1 H, 6-H^{II}), 3.14 (dd, *J* = 8.9 Hz, *J* = 9.7 Hz, 1 H, 4-H^{II}), 3.45–3.49 (m, 5 H, 2-H^{II}, OC$\underline{H}$H'CH_3, OC$\underline{H}_3$), 3.67–3.79 (m, 3 H, 6-H^{I}, 6'-H^{II}, OCH$\underline{H}$'CH_3), 4.02 (m, 1 H, 5-H^{I}), 4.05–4.14 (m, 2 H, 6'-H^{I}, 3-H^{II}), 4.20–4.27 (m, 2 H, 4-H^{I}, 5-H^{II}), 4.57, 4.96 (2 × d, *J* = 11.2 Hz, 2 H, PhC$\underline{H}_2$), 4.61 (d, $J_{1,2}$ = 3.3 Hz, 1 H, 1-H^{II}), 4.65–4.68 (m, 2 H, 1-H^{I}, PhC$\underline{H}$H'), 4.79 (d, *J* = 11.4 Hz, 1 H, PhCH$\underline{H}$'), 4.82, 5.02 (2 × d, *J* = 11.0 Hz, 2 H, PhC$\underline{H}_2$), 5.18 (dd, $J_{1,2}$ = 1.8 Hz, $J_{2,3}$ = 3.2 Hz, 1 H, 2-H^{I}), 5.32 (dd, $J_{2,3}$ = 3.4 Hz, $J_{3,4}$ = 11.0 Hz, 1 H, 3-H^{I}), 7.17 (m, 1 H, Ar-H), 7.24–7.38 (m, 15 H, Ar-H), 7.49–7.60 (m, 2 H, Ar-H), 7.72 (m, 1 H, Ar-H); ^{13}C NMR (100 MHz, $CDCl_3$): δ = –5.0, –4.9 (2 × q, $Si(CH_3)_2$), 15.1 (q, $OCH_2\underline{C}H_3$), 18.3 (s, Si$\underline{C}$(CH_3)$_3$), 20.1, 21.1 (2 × s, 2 × C(O)$\underline{C}H_3$), 26.0 (q, SiC($\underline{C}H_3$)$_3$), 47.1 (t, C-6^{II}), 54.6 (d, C-4^{I}), 56.1 (q, O$\underline{C}H_3$), 62.9 (t, O$\underline{C}H_2CH_3$), 63.9 (t, C-6^{I}), 66.3 (d, C-3^{I}), 69.9, 70.1 (2 × d, C-2^{I}, C-5^{II}), 71.7 (d, C-5^{I}), 73.4, 74.7, 75.6 (3 × t, 3 × Ph$\underline{C}H_2$), 80.1 (d, C-2^{II}), 81.2, 81.3 (2 × d, C-3^{II}, C-4^{II}), 96.4 (d, C-1^{I}), 98.3 (d, C-1^{II}), 123.9, 127.7, 127.8, 127.9, 127.9, 128.1, 128.2, 128.4, 128.5, 128.5, 128.6, 129.2, 131.0, 131.9, 133.4 (15 × d, Ar-$\underline{C}$H), 132.4, 138.2, 138.5, 138.8, 148.2 (5 × s, 5 × Ar-C), 169.3, 170.5 (2 × s, 2 × C=O); MS (ESI^{+}): *m/z* (%) 1075 (4) [M + K^{+}], 1059 (100) [M + Na^{+}]; HRMS–ESI *m/z* [M + Na^{+}] calcd for $C_{52}H_{68}N_2O_{16}SSiNa$: 1059.3951; found: 1059.3952.

_N_-(methyl 2,3,4-tri-_O_-benzyl-6-deoxy-α-D-glucopyranosid-6-yl)-_N_-(ethyl 4-deoxy-α-D-mannopyranosid-4-yl)-2-nitrobenzenesulfonamide (19)

From **13**: Alkene **13** (205 mg, 0.223 mmol) was dissolved in acetone/H_2O [4:1] (5 mL) at RT. AcOH (13 µL, 0.22 mmol), then $K_2OsO_4{\cdot}2H_2O$ (8 mg, 22 µmol), and then NMO (50% aq., 208 µL, 0.892 mmol) were added. The reaction mixture slowly changed colour from yellow to black. TLC (toluene–EtOAc, 4:3) showed a slow but steady conversion of the starting material (R_f 0.9) to a single product (R_f 0.3). After 4 days, the reaction was quenched by $NaHSO_3$ (satd. aq., 2 mL), and the mixture was left to stir for 20 h, after which time TLC (toluene–EtOAc, 4:3) showed the complete conversion of the intermediate diol **16** (R_f 0.3) into a new product (R_f 0.1). $NaHCO_3$ (satd. aq., 5 mL) was added, and the mixture was extracted with EtOAc (3 × 10 mL). The combined organic extracts were dried ($MgSO_4$), filtered and concentrated in vacuo. The crude product was purified by column chromatography (toluene–EtOAc, 1:3) to give triol **19** (149 mg, 80%) as an oil.

From **16**: Diol **16** (47 mg, 0.050 mmol) was dissolved in MeOH/HCl (1 M aq.) [10:1] (2 mL), and stirred at RT for 30 min, after which time TLC (toluene–EtOAc, 1:1) showed the complete conversion of the starting material (R_f 0.6) into a single product (R_f 0.2). The mixture was concentrated in vacuo. The crude product was purified by column chromatography (toluene–EtOAc, 2:5) to give triol **19** (31 mg, 74%) as an oil; $[\alpha]_D^{25}$ +23.5 (*c* 1.0, $CHCl_3$); IR (film): 3435 (O–H), 1544 (N–O), 1371 cm^{-1}; ^1H NMR (400 MHz, $CDCl_3$): δ = 1.13 (t, *J* = 7.0 Hz, 3 H, $OCH_2C\underline{H}_3$), 2.72 (br s, 1 H, OH), 2.86 (d, *J* = 7.6 Hz, 1 H, OH), 3.02–3.14 (m, 3 H, 4-H^{II}, 6-H^{II}, OH), 3.41 (s, 3 H, OCH_3), 3.45–3.52 (m, 2 H, 2-H^{II}, $OC\underline{H}H'CH_3$), 3.62–3.73 (m, 3 H, 6-H^{I}, 6'-H^{II}, $OCH\underline{H}'CH_3$), 3.80–3.87 (m, 4 H, 2-H^{I}, 3-H^{I}, 5-H^{I}, 6'-H^{I}), 4.01 (at, *J* = 10.2 Hz, 1 H, 4-H^{I}), 4.10 (at, *J* = 9.2 Hz, 1 H, 3-H^{II}), 4.28 (at, *J* = 9.7 Hz, 1 H, 5-H^{II}), 4.51 (d, $J_{1,2}$ = 3.2 Hz, 1 H, 1-H^{II}), 4.57, 5.02 (2 × d, *J* = 11.5 Hz, 2 H, $PhC\underline{H}_2$), 4.66 (d, *J* = 12.1 Hz, 1 H, $PhC\underline{H}H'$), 4.78–4.82 (m, 2 H, $PhC\underline{H}H'$, $PhCH\underline{H}'$), 4.84 (s, 1 H, 1-H^{I}), 5.00 (d, *J* = 10.8 Hz, 1 H, $PhCH\underline{H}'$), 7.13–7.83 (m, 19 H, Ar-H); ^{13}C NMR (100 MHz, $CDCl_3$): δ = 15.1 (q, $OCH_2\underline{C}H_3$), 47.0 (t, C-6^{II}), 56.4 (q, $O\underline{C}H_3$), 56.4 (d, C-4^{I}), 61.7 (t, C-6^{I}), 63.3 (t, $O\underline{C}H_2CH_3$), 66.2, 70.1, 71.4 (3 × d, C-2^{I}, C-3^{I}, C-5^{I}), 70.2 (d, C-5^{II}), 73.5, 74.6, 75.8 (3 × t, 3 × $Ph\underline{C}H_2$), 80.1 (d, C-2^{II}), 80.5 (d, C-4^{II}), 81.5 (d, C-3^{II}), 98.5 (d, C-1^{II}), 99.0 (d, 1-H^{I}), 123.9, 127.7, 127.8, 127.9, 128.1, 128.1, 128.3, 128.6, 128.6, 129.1, 131.4, 132.2, 133.8 (13 × d, Ar-CH); 131.5, 139.2, 138.3, 138.6, 147.8 (5 × s, Ar-CH); MS (ESI^+): *m/z* (%) 877 (3) [M + K^+], 861 (100) [M + Na^+]; HRMS–ESI *m/z* [M + Na^+] calcd for $C_{42}H_{50}N_2O_{14}SNa$: 861.2875; found: 861.2883.

N-(methyl 2,3,4-tri-O-benzyl-6-deoxy-α-D-mannopyranosid-6-yl)-N-(ethyl 4-deoxy-α-D-mannopyranosid-4-yl)-2-nitrobenzenesulfonamide (20)

Diol **17** (65 mg, 0.068 mmol) was dissolved in MeOH/HCl (1 M aq.) [10:1] (5 mL), and stirred at RT for 20 min, after which time TLC (toluene–EtOAc, 1:1) showed complete conversion of starting material (R_f 0.6) into a single product (R_f 0.2). The reaction was quenched by the addition of Et_3N (0.5 mL) and the mixture concentrated in vacuo. The crude product was purified by column chromatography (toluene–EtOAc, 1:3) to give triol **20** (49 mg, 86%) as an oil; IR (film): 3524 (O–H), 1544 (N–O), 1372 cm^{-1}; ^{1}H NMR (500 MHz, $CDCl_3$): δ = 1.15 (t, J = 7.1 Hz, 3 H, $OCH_2C\underline{H}_3$), 2.78 (br s, 3 H, OH), 3.21 (dd, $J_{5,6}$ = 9.9 Hz, $J_{6,6'}$ = 15.8 Hz, 1 H, 6-H^{II}), 3.35 (s, 3 H, $OC\underline{H}_3$), 3.50 (dq, J = 7.1 Hz, J_{gem} = 9.7 Hz, 1 H, $OC\underline{H}H'CH_3$), 3.61 (at, J = 9.5 Hz, 1 H, 4-H^{II}), 3.69 (dq, J = 7.1 Hz, J_{gem} = 9.7 Hz, 1 H, $OCH\underline{H}'CH_3$), 3.78–3.82 (m, 3 H, 2-H^x, 6-H^I, 6'-H^{II}), 3.88 (br s, 1 H, 2-H^y), 3.94–4.09 (m, 5 H, 3-H^I, 4-H^I, 5-H^I,6'-H^I, 3-H^{II}), 4.29 (at, J = 9.3 Hz, 1 H, 5-H^{II}), 4.59–4.67 (m, 5 H, 1-H^x, 2 × $PhC\underline{H}H'$, $PhC\underline{H}_2$), 4.74 (d, J = 12.3 Hz, 1 H, $PhCH\underline{H}'$), 4.88 (br s, 1 H, 1-H^y), 5.10 (d, J = 11.6 Hz, 1 H, $PhCH\underline{H}'$), 7.11–7.85 (m, 19 H, Ar-H); ^{13}C NMR (125 MHz, $CDCl_3$): δ = 15.1 (q, $OCH_2\underline{C}H_3$), 47.2 (t, C-6^{II}), 55.8 (q, $O\underline{C}H_3$), 56.6 (d, C-4^I), 61.9 (t, C-6^I), 63.2 (t, $O\underline{C}H_2CH_3$), 66.1, 70.3, 79.8 (3 × d, C-3^I, C-5^I, C-3^{II}), 71.2 (d, C-5^{II}), 71.4 (d, C-2^y), 72.1, 72.9, 74.7 (3 × t, $Ph\underline{C}H_2$), 74.7 (d, C-2^x), 77.2 (d, C-4^{II}), 99.0 (d, C-1^y), 99.1 (d, C-1^x), 123.8, 127.6, 127.6, 127.8, 127.8, 127.8, 128.3, 128.5, 128.5, 129.1, 131.4, 132.0, 133.8 (13 × d, Ar-$\underline{C}$H), 131.3, 138.3, 138.8, 147.8 (4 × s, Ar-C); MS (ESI$^+$): m/z (%) 877 (2) [M + K$^+$], 861 (100) [M + Na$^+$]; HRMS–ESI m/z [M + Na$^+$] calcd for $C_{42}H_{50}N_2O_{14}Na$: 861.2875; found: 861.2881.

N-(methyl 6-deoxy-α-D-glucopyranosid-6-yl)-N-(ethyl 4-deoxy-α-D-mannopyranosid-4-yl)-amine (21)

NH_3 (ca. 20 mL) was condensed at –78 °C and Na (100 mg, 4.3 mmol) was added, giving the mixture a dark blue colour. A few drops of MeOH were added and the colouration remained. Triol **19** (145 mg, 0.173 mmol) was dissolved in THF (2 mL) and transferred to the reaction vessel, after which the mixture turned brown. Further Na (100 mg, 4.3 mmol) was added, and the dark blue colour returned. After 30 min, the reaction was quenched by the addition of NH_4Cl (s) (465 mg, 8.68 mmol). After a few min, the blue colour disappeared. The mixture was allowed to warm to RT for the ammonia to evaporate, and then concentrated in vacuo. The residue was dissolved in $CHCl_3$–MeOH, 1:1 and filtered to remove inorganic salts. The procedure was repeated, after which the crude product was concentrated in vacuo and purified by column chromatography (MeOH–$CHCl_3$–NH_4OH (25% aq.), 5:5:1) to give diglycoside **21** (52 mg, 78%) as an oil; ^{1}H NMR (500 MHz, D_2O): δ = 1.25 (t, J = 7.1 Hz, 3 H, $OCH_2C\underline{H}_3$), 2.85 (at J = 10.3 Hz, 1 H, 4-H^I), 2.87

(dd, $J_{5,6}$ = 8.2 Hz, $J_{6,6'}$ = 12.6 Hz, 1 H, 6-H^{II}), 3.14 (dd, $J_{5,6'}$ = 2.7 Hz, $J_{6,6'}$ = 12.5 Hz, 1 H, 6'-H^{II}), 3.35 (at, J = 9.4 Hz, 1 H, 4-H^{II}), 3.47 (s, 3 H, OCH_3), 3.59–3.63 (m, 2 H, 2-H^{II}, $OC\underline{H}H'CH_3$), 3.66–3.73 (m, 3 H, 3-H^{I} or 5-H^{I} and 3-H^{II}, 5-H^{II}), 3.78–3.83 (m, 2 H, 6-H^{I}, $OCH\underline{H}'CH_3$), 3.87 (dd, J = 3.4 Hz, J = 10.3 Hz, 1 H, 3-H^{I} or 5-H^{I}), 3.90–3.94 (m, 2 H, 2-H^{I}, 6'-H^{I}), 4.84 (d, $J_{1,2}$ = 3.8 Hz, 1 H, 1-H^{II}), 4.92 (d, $J_{1,2}$ = 1.6 Hz, 1 H, 1-H^{I}); ^{13}C NMR (125.1 MHz, D_2O): δ = 14.2 (q, $OCH_2\underline{C}H_3$), 49.1 (t, C-6^{II}), 55.5 (q, OCH_3), 56.0 (d, C-4^{I}), 61.9 (t, C-6^{I}), 63.6 (t, $O\underline{C}H_2CH_3$), 69.1, 70.8, 72.2, 73.1 (4 × d, C-3^{I}, C-5^{I}, C-3^{II}, C-5^{II}), 69.7 (d, C-2^{I}), 71.4 (d, C-2^{II}), 71.9 (d, C-4^{II}), 99.5 (d, C-1^{II}), 99.7 (d, C-1^{I}); MS (ESI$^+$): *m/z* (%) 789 (26) [2M + Na^+], 406 (41) [M + Na^+], 384 (100) [M + H^+]; HRMS–ESI *m/z* [M + Na^+] calcd for $C_{15}H_{29}NO_{10}Na$: 406.1684; found: 406.1672.

_N_-(methyl 6-deoxy-α-D-mannopyranosid-6-yl)-_N_-(ethyl 4-deoxy-α-D-mannopyranosid-4-yl)-amine (22)

NH_3 (ca. 20 mL) was condensed at –78 °C, and Na (100 mg, 4.3 mmol) was added, giving the mixture a dark blue colour. Triol **20** (109 mg, 0.130 mmol) was dissolved in THF (2 mL) and transferred to the reaction vessel, after which the mixture turned brown. Further Na (100 mg, 4.3 mmol) was added, and the dark blue colour returned. After 30 min, the reaction was quenched by the addition of NH_4Cl (s) (465 mg, 8.68 mmol). After a few min, the blue colour disappeared. The mixture was allowed to warm to RT for the ammonia to evaporate, and then concentrated in vacuo. The residue was dissolved in $CHCl_3$–MeOH, 1:1 and filtered to remove inorganic salts. The procedure was repeated, after which the crude product was concentrated in vacuo and purified by column chromatography (MeOH–$CHCl_3$–NH_4OH (25%), 20:10:1) to give diglycoside **22** (27 mg, 53%) as an oil; ^{1}H NMR (400 MHz, D_2O): δ = 1.13 (t, J = 7.1 Hz, 3 H, $OCH_2C\underline{H}_3$), 2.92–2.99 (m, 2 H, 4-H^{I}, 6-H^{II}), 3.19 (d, J = 12.7 Hz, 1 H, 6'-H^{II}), 3.37 (s, 3 H, $OC\underline{H}_3$), 3.45–3.53 (m, 2 H, 4-H^{II}, $OC\underline{H}H'CH_3$), 3.61 (m, 1 H, 5-H^{II}), 3.65–3.75 (m, 4 H, 5-H^{I}, 6-H^{I}, 3-H^{II}, $OCH\underline{H}'CH_3$), 3.78 (m, 1 H, 6'-H^{I}), 3.82 (dd, $J_{1,2}$ = 1.7 Hz, $J_{2,3}$ = 3.3 Hz, 1 H, 2-H^{I}), 3.85 (dd, $J_{1,2}$ = 1.7 Hz, $J_{2,3}$ = 3.4 Hz, 1 H, 2-H^{II}), 3.88 (dd, $J_{2,3}$ = 3.1 Hz, $J_{3,4}$ = 10.4 Hz, 1 H, 3-H^{I}), 4.68 (d, $J_{1,2}$ = 1.7 Hz, 1 H, 1-H^{II}), 4.81 (d, $J_{1,2}$ = 1.7 Hz, 1 H, 1-H^{I}); ^{13}C NMR (100 MHz, D_2O): δ = 14.0 (q, $OCH_2\underline{C}H_3$), 48.4 (t, C-6^{II}), 55.1 (q, $O\underline{C}H_3$), 56.3 (d, C-4^{I}), 61.7 (t, C-6^{I}), 63.6 (t, $O\underline{C}H_2CH_3$), 67.8 (d, C-3^{I}), 67.8 (d, C-4^{II}), 69.4 (d, C-2^{I}), 69.8 (d, C-2^{II}), 70.2, 70.3 (2 × d, C-5^{I}, C-3^{II}, C-5^{II}), 99.5 (d, C-1^{I}), 101.0 (d, C-1^{II}); MS (ESI$^+$): *m/z* (%) 789 (6) [2M + Na^+], 406 (100) [M + Na^+], 384 (10) [M + H^+]; HRMS–ESI *m/z* [M + Na^+] calcd for $C_{15}H_{29}NO_{10}Na$: 406.1684; found: 406.1684.

Ethyl 4-*O*-trichloroacetimidoyl-6-*O*-(*tert*-butyldimethylsilyl)-2,3-dideoxy-α-D-threo-hex-2-enopyranoside (23)

Allylic alcohol **2** (149 mg, 0.517 mmol) was dissolved in freshly distilled CH_2Cl_2 (10 mL) under Ar. Trichloroacetonitrile (0.13 mL, 1.29 mmol) and DBU (0.09 mL, 0.62 mmol) were added, and the reaction mixture was stirred for 1 h 35 min. After this time, TLC (pentane–EtOAc, 6:1) indicated the complete consumption of starting material (R_f 0.1) and the formation of a major product (R_f 0.9). The reaction mixture was concentrated to dryness to give a black oil (650 mg). The crude product was purified by column chromatography (toluene, 1% Et_3N) to give trichloroacetimidate **23** (223 mg, 99%) as a yellow oil; $[\alpha]_D^{24}$ –81.0 (*c* 0.7, $CHCl_3$); IR (film): 1662 (C=O) cm^{-1}; ^{1}H NMR (400 MHz, $CDCl_3$): δ = 0.04, 0.06 (2 × s, 6 H, $Si(CH_3)_2$), 0.88 (s, 9 H, $SiC(CH_3)_3$), 1.25 (t, J = 7.2 Hz, 3 H, $OCH_2C\underline{H}_3$), 3.58 (dq, J_{gem} = 10.0 Hz, J = 7.2 Hz, 1 H, $OC\underline{H}H'CH_3$), 3.81–3.93 (m, 3 H, 6-H, 6'-H, $OCH\underline{H}'CH_3$), 4.26 (dat, J = 6.8 Hz, $J_{4,5}$ = 2.4 Hz, 1 H, 5-H), 5.11 (d, $J_{1,2}$ = 2.8 Hz, 1 H, 1-H), 5.17 (dd, $J_{3,4}$ = 5.6 Hz, $J_{4,5}$ = 2.4 Hz, 1 H, 4-H), 6.07 (dd, $J_{2,3}$ = 10.0 Hz, $J_{1,2}$ = 2.8 Hz, 1 H, 2-H), 6.31 (dd, $J_{2,3}$ = 10.0 Hz, $J_{3,4}$ = 5.2 Hz, 1 H, 3-H), 8.29 (s, 1 H, $C(N\underline{H})CCl_3$); ^{13}C NMR (400 MHz, $CDCl_3$): δ = –5.25, –5.30 (2 × q, $Si(CH_3)_2$), 15.4 (q, $OCH_2\underline{C}H_3$), 18.3 (s, $Si\underline{C}(CH_3)_3$), 25.9 (q, $SiC(\underline{C}H_3)_3$), 61.9 (t, C-6), 63.9 (t, $O\underline{C}H_2CH_3$), 67.5 (d, C-5), 70.1 (d, C-4), 91.6 (s, CCl_3), 93.9 (d, C-1), 124.4 (d, C-3), 131.7 (d, C-2), 162.1 (d, C=NH); MS (ESI^+): *m/z* 454 (100) [M + H^+]; HRMS–ESI *m/z* [M + H^+] calcd for $C_{16}H_{28}Cl_3NO_4Si$: 454.0751; found: 454.0763.

***N*-(methyl 2,3,4-tri-*O*-benzyl-6-deoxy-α-D-glucopyranosid-6-yl)-*N*-(ethyl 6-*O*-(*tert*-butyldimethylsilyl)-2,3,4-trideoxy-α-D-*threo*-hex-4-enopyranosid-2-yl)-amine (24), *N*-(methyl 2,3,4-tri-*O*-benzyl-6-deoxy-α-D-glucopyranosid-6-yl)-*N*-(ethyl 6-*O*-(*tert*-butyldimethylsilyl)-2,3,4-trideoxy-α-D-*threo*-hex-2-enopyranosid-4-yl)-amine (25), and *N*-(methyl 2,3,4-tri-*O*-benzyl-6-deoxy-α-D-glucopyranosid-6-yl)-*N*-(ethyl 6-*O*-(*tert*-butyldimethylsilyl)-2,3,4-trideoxy-α-D-*erythro*-hex-2-enopyranosid-4-yl)-amine (26)**

Trichloroacetimidate **23** (95 mg, 0.219 mmol), amine **5** (305 mg, 0.659 mmol), TMPP (trimethylolpropane phosphite , 3.5 mg, 0.022 mmol) and $Pd(dba)_2$ (6.4 mg, 0.011 mmol) were added to a Schlenk tube (10 mL). The tube was put under vacuum and backfilled with argon. This process was repeated three times. Degassed MeCN (1.3 mL) and Et_3N (70 µL, 0.678 mmol) were added, and the reaction mixture was stirred at RT. After 19 h, TLC (pentane–EtOAc, 2:1) indicated the complete consumption of trichloroacetimidate (R_f 0.9) and the formation of what appeared to be three products (R_f 0.7, 0.6, 0.3). The reaction mixture was evaporated to give a brown oil (423 mg). The crude product was purified by column chromatography (Pentane–EtOAc, 3:1) to give **24**

(71 mg, 44%), an inseparable mixture (19 mg), and a mixture of **25**–**27** (57 mg) which was separated by column chromatography (CH_2Cl_2–EtOAc, 7:1) to give **25** (24 mg, 15%), **26** (8 mg, 5%), and **27** (10 mg, 6%).

Data for **24**: $[\alpha]_D^{24}$ +55.9 (*c* 1.0, $CHCl_3$); ^{1}H NMR (400 MHz, $CDCl_3$): δ = 0.04, 0.05 (2 × s, 6 H, $Si(CH_3)_2$), 0.88 (s, 9 H, $C(CH_3)_3$), 1.22 (t, *J* = 7.2 Hz, 3 H, $OCH_2C\underline{H}_3$), 2.74 (dd, $J_{6,6'}$ = 12.4 Hz, $J_{5,6}$ = 6.0 Hz, 1 H, 6-H^{II}), 2.86 (m, 1 H, 2-H^{I}), 3.00 (dd, $J_{6,6'}$ = 12.4 Hz, $J_{5,6'}$ = 2.8 Hz, 1 H, 6'-H^{II}), 3.36 (s, 3 H, OCH_3), 3.46–3.62 (m, 4 H, 6-H^{I}, 2-H^{II}, 4-H^{II}, $OC\underline{H}H'CH_3$), 3.69–3.72 (m, 2 H, 6'-H^{I}, 5-H^{II}), 3.81 (dq, J_{gem} = 9.6 Hz, *J* = 7.2 Hz, 1 H, $OCH\underline{H}'CH_3$), 3.97 (at, *J* = 9.2 Hz, 1 H, 3-H^{II}), 4.13 (m, 1 H, 5-H^{I}), 4.53 (d, $J_{1,2}$ = 3.6 Hz, 1 H, 1-H^{II}), 4.66, 4.79 (2 × d, *J* = 12.0 Hz, 2 H, $PhC\underline{H}_2$), 4.62, 4.88 (2 × d, *J* = 11.2 Hz, 2 H, $PhC\underline{H}_2$), 4.81, 4.97 (2 × d, *J* = 10.8 Hz, 2 H, $PhC\underline{H}_2$), 4.86 (s, 1 H, 1-H^{I}), 5.81–5.89 (m, 2 H, 3-H^{I}, 4-H^{I}), 7.27–7.37 (m, 15 H, Ar-H); ^{13}C NMR (400 MHz, $CDCl_3$): δ = –5.2, –5.2 (2 × q, $Si(CH_3)_2$), 15.3 (q, $OCH_2\underline{C}H_3$), 18.5 (s, $Si\underline{C}(CH_3)_3$), 26.0 (q, $SiC(\underline{C}H_3)_3$), 47.1 (t, C-6^{II}), 54.4 (d, C-2^{I}), 55.3 (q, OCH_3), 63.7 (t, $O\underline{C}H_2CH_3$), 65.6 (d, C-6^{I}), 69.1 (d, C-5^{I}), 70.6 (d, C-5^{II}), 73.5, 75.1, 75.8 (3 × t, 3 × $Ph\underline{C}H_2$), 79.1 (d, C-4^{II}), 79.1 (d, C-2^{II}), 82.2 (d, C-3^{II}), 98.0 (d, C-1^{II}), 98.9 (d, C-1^{I}), 125.7 (d, C-3^{I}), 128.3 (d, C-4^{I}), 127.7, 127.8, 128.0, 128.0, 128.1, 128.2, 128.5, 128.5, 128.6 (9 × d, Ar-CH), 138.4, 138.6, 139.0 (3 × s, 3 × Ar-C); MS (ESI$^+$): *m/z* (%) 734 (100) [M + H$^+$]; HRMS–ESI *m/z* [M + H$^+$] calcd for $C_{42}H_{60}NO_8Si$: 734.4083; found: 734.4032.

Data for **25**: $[\alpha]_D^{24}$ –35.0 (*c* 1.0, $CHCl_3$); ^{1}H NMR (400 MHz, $CDCl_3$): δ = 0.02, 0.04 (2 × s, 6 H, $Si(CH_3)_2$), 0.86 (s, 9 H, $SiC(CH_3)_3$), 1.23 (t, *J* = 7.2 Hz, 3 H, $OCH_2C\underline{H}_3$), 2.77 (dd, $J_{6,6'}$ = 12.4 Hz, $J_{5,6}$ = 4.4 Hz, 1 H, 6-H^{II}), 2.89 (dd, $J_{3,4}$ = 5.2 Hz, $J_{4,5}$ = 3.2 Hz, 1 H, 4-H^{I}), 3.06 (dd, $J_{6,6'}$ = 12.4 Hz, $J_{5,6'}$ = 2.8 Hz, 1 H, 6'-H^{II}), 3.34 (s, 3 H, OCH_3), 3.46 (m, 1 H, 2-H^{II}), 3.52, 3.90 (2 × m, 2 H, $OC\underline{H}_2CH_3$), 3.64 (m, 1 H, 4-H^{II}), 3.69 (m, 1 H, 5-H^{II}), 3.81 (d, $J_{5,6}$ = 6.4 Hz, 2 H, 6-H^{I}, 6'-H^{I}), 3.96 (at, *J* = 9.2 Hz, 1 H, 3-H^{II}), 4.05 (dat, $J_{5,6}$ = 6.4 Hz, *J* = 2.8 Hz, 1 H, 5-H^{I}), 4.49 (d, $J_{1,2}$ = 3.6 Hz, 1 H, 1-H^{II}), 4.64, 4.87 (2 × d, *J* = 10.8 Hz, 2 H, $PhC\underline{H}_2$), 4.66, 4.80 (2 × d, *J* = 12.0 Hz, 2 H, $PhC\underline{H}_2$), 4.81, 4.96 (2 × d, *J* = 10.8 Hz, 2 H, $PhC\underline{H}_2$), 4.96 (m, 1 H, 1-H^{I}), 5.82 (dd, $J_{2,3}$ = 10.0 Hz, $J_{1,2}$ = 2.8 Hz, 1 H, 2-H^{I}), 6.19 (dd, $J_{2,3}$ = 10.0 Hz, $J_{3,4}$ = 5.2 Hz, 1 H, 3-H^{I}), 7.26–7.32 (m, 15 H, Ar-H); ^{13}C NMR (400 MHz, $CDCl_3$): δ = –5.2, –5.3 (2 × q, $Si(CH_3)_2$), 15.4 (q, $OCH_2\underline{C}H_3$), 18.4 (s, $Si\underline{C}(CH_3)_3$), 25.9 (q, $SiC(\underline{C}H_3)_3$), 46.8 (t, C-6^{II}), 50.4 (d, C-4^{I}), 55.2 (q, OCH_3), 63.3 (t, $O\underline{C}H_2CH_3$), 63.5 (d, C-6^{I}), 70.7 (d, C-5^{II}), 71.9 (d, C-5^{I}), 73.6, 75.2, 75.9 (3 × t, 3 × $\underline{C}H_2Ph$), 78.6 (d, C-4^{II}), 80.2 (d, C-2^{II}), 82.2 (d, C-3^{II}), 94.3 (d, C-1^{I}), 98.2 (d, C-1^{II}), 127.4 (d, C-2^{I}), 127.7, 127.8, 127.9, 128.0, 128.1, 128.3, 128.5, 128.5, 128.6, (9 × d, Ar-CH), 131.0 (d, C-3^{I}), 138.4, 138.7, 139.0, (3 × s, 3 × Ar-C); MS (ESI$^+$): *m/z* (%) 734 (100) [M + H$^+$]; HRMS–ESI *m/z* [M + H$^+$] calcd for $C_{42}H_{60}NO_8Si$: 734.4083; found: 734.4108.

Data for **26**: $[\alpha]_D^{24}$ +32.1 (*c* 0.7, $CHCl_3$); ^{1}H NMR (400 MHz, $CDCl_3$): δ = 0.05 (s, 6 H, $Si(CH_3)_2$), 0.88 (s, 9 H, $SiC(C\underline{H}_3)_3$), 1.22 (t, *J* = 7.2 Hz, 3 H, $OCH_2C\underline{H}_3$), 2.74–2.81 (m, 2 H, 6-H^{II}, 6'-H^{II}), 3.03 (dat, $J_{4,5}$ = 10.0 Hz, *J* = 1.6 Hz, 1 H, 4-H^{I}), 3.34 (s, 3 H, OCH_3), 3.36 (at, *J* = 9.2 Hz, 1 H, 4-H^{II}), 3.47 (dd, $J_{2,3}$ = 9.6 Hz, $J_{1,2}$ = 3.6 Hz, 1 H, 2-H^{II}), 3.49–3.56 (m, 1 H, $OC\underline{H}H'CH_3$), 3.57–3.62 (m, 1 H, 5-H^{I}), 3.67–3.72 (m, 1 H, 5-H^{II}), 3.76 (dd, $J_{6,6'}$ = 11.2 Hz, $J_{5,6}$ = 6.0 Hz, 1 H, 6-H), 3.82–3.94 (m, 2 H, 6'-H, $OCH\underline{H}'CH_3$), 3.98 (t, *J* = 9.6 Hz, 1 H, 3-H^{II}), 4.51 (d, $J_{1,2}$ = 3.6 Hz, 1 H, 1-H^{II}), 4.59, 4.88 (2 × d, *J* = 11.2 Hz, 2 H, $PhC\underline{H}_2$), 4.66, 4.78 (2 × d, *J* = 12.0 Hz, 2 H, $PhC\underline{H}_2$), 4.81 (d, *J* = 11.2 Hz, 1 H, $PhC\underline{H}H'$), 4.96–4.99 (m, 2 H, 1-H, $PhCH\underline{H}'$), 5.72 (dat, $J_{2,3}$ = 10.0 Hz, *J* 2.4 Hz, 1 H, 2-H^{I}), 5.96 (br d, $J_{2,3}$ = 10.0 Hz, 1 H, 3-H^{I}), 7.26–7.37 (m, 15 H, Ar-H); ^{13}C NMR (400 MHz, $CDCl_3$): δ = –5.1, –5.1 (2 × q, $Si(CH_3)_2$), 15.5 (q, $OCH_2\underline{C}H_3$), 18.6 (s, $Si\underline{C}(CH_3)_3$), 26.1 (q, $SiC(\underline{C}H_3)_3$), 47.2 (t, C-6^{II}), 52.7 (d, C-4^{I}), 55.2 (q, OCH_3), 63.6 (t, $O\underline{C}H_2CH_3$), 64.4 (d, C-6^{I}), 70.7 (d, C-5^{II}), 71.7 (d, C-5^{I}), 73.5, 75.2, 75.9 (3 × t, 3 × $Ph\underline{C}H_2$), 79.6 (d, C-4^{II}), 80.3 (d, C-2^{II}), 82.2 (d, C-3^{II}), 94.1 (d, C-1^{I}), 97.9 (d, C-1^{II}), 125.8 (d, C-2^{I}), 127.7, 127.9, 128.0, 128.1, 128.1, 128.2, 128.5, 128.6, 128.6 (9 × d, Ar-CH), 132.1 (d, C-3^{I}), 138.3, 138.4, 138.9, (3 × s, 3 × Ar-C); MS (ESI$^+$): *m/z* (%) 734 (100) [M + H$^+$]; HRMS–ESI *m/z* [M + H$^+$] calcd for $C_{42}H_{60}NO_8Si$: 734.4083; found: 734.4108.

Data for **27**: $[\alpha]_D^{24}$ –24.1 (*c* 0.8, $CHCl_3$); IR (film): 1694 (C=O) cm^{-1}; ^{1}H NMR (400 MHz, $CDCl_3$): δ = 0.02, 0.04 (2 × s, 6 H, $Si(CH_3)_2$), 0.86 (s, 9 H, $SiC(CH_3)_3$), 1.23 (t, *J* = 6.8 Hz, 3 H, $OCH_2C\underline{H}_3$), 3.28 (at, *J* = 9.2 Hz, 1 H, 4-H^{II}), 3.33 (s, 3 H, OCH_3), 3.38–3.46 (m, 3 H, 2-H^{II}, 6-H^{II}, 6'-H^{II}), 3.64 (m, 1 H, 5-H^{II}), 3.73–3.79 (m, 2 H, 6-H^{I}, 6'-H^{I}), 3.54, 3.88 (2 × m, 2 H, $OC\underline{H}_2CH_3$), 3.98 (t, *J* = 9.2 Hz, 1 H, 3-H^{II}), 4.18 (m, 1 H, 5-H^{I}), 4.50 (d, $J_{1,2}$ = 3.6 Hz, 1 H, 1-H^{II}), 4.59–4.66 (m, 2 H, $PhC\underline{H}_2$), 4.78–4.86 (m, 3 H, $PhC\underline{H}_2$, $PhC\underline{H}H'$), 4.95 (dd, $J_{3,4}$ = 5.2 Hz, $J_{4,5}$ = 2.4 Hz, 1 H, 4-H^{I}), 4.98 (d, *J* = 10.8 Hz, 1 H, $PhCH\underline{H}'$), 5.05 (m, 1 H, 1-H^{I}), 5.98 (dd, $J_{2,3}$ = 10.0 Hz, $J_{1,2}$ = 3.2 Hz, 1 H, 2-H^{I}), 6.15 (dd, $J_{2,3}$ = 10.0 Hz, $J_{3,4}$ = 5.2 Hz, 1 H, 3-H^{I}), 7.27–7.36 (m, 15 H, Ar-H); ^{13}C NMR (400 MHz, $CDCl_3$): δ = –5.2, –5.3 (2 × q, $Si(CH_3)_2$), 15.3 (q, $OCH_2\underline{C}H_3$), 18.3 (s, $Si\underline{C}(CH_3)_3$), 25.9 (q, $SiC(\underline{C}H_3)_3$), 41.7, 55.4 (q, OCH_3), 62.2, 63.5, 63.8, 69.3, 70.2, 73.6, 75.3 75.9, 78.7, 80.0, 81.9, 91.9 (12 × d, t), 93.9 (d, C-1^{I}), 98.1 (d, C-1^{II}), 126.4 (d, C-2^{I}), 127.7, 128.0, 128.1, 128.1, 128.3, 128.4, 128.5, 128.6, 128.7 (9 × d, Ar-CH), 130.4 (d, C-3^{I}), 138.0, 138.2, 138.8, (3 × s, 3 × Ar-C), 155.9, 163.4 (2 × s); MS (ESI$^+$): *m/z* (%) 800 (100) [M + H$^+$]; HRMS–ESI *m/z* [M + H$^+$] calcd for $C_{42}H_{60}NO_8Si$: 800.3806; found: 800.3790.

Assays against glycosidases

The commercially available glycosidases tested were: α-L-Fucosidase (bovine kidney: EC 3.2.1.51), α-galactosidase (coffee beans: EC 3.2.1.22), β-galactosidase (*Escherichia coli* and

Aspergillus orizae: EC 3.2.1.23), α-glucosidase (yeast and rice: EC 3.2.1.20), amyloglucosidase (*Aspergillus niger*: EC 3.2.1.3), β-glucosidase (almonds: EC 3.2.1.21), α-mannosidase (jack beans: EC 3.2.1.24), β-mannosidase (snail: EC 3.2.1.25), β-xylosidase (*Aspergillus niger*: EC 3.2.1.37), β-*N*-acetylglucosaminidase (jack beans: EC 3.2.1.30). The experiments were performed essentially as previously described [1,2]. Briefly, 0.01–0.5 units/mL of enzyme and inhibitor were pre-incubated for 5 min at RT, and the reaction was started by addition of the substrate, buffered to the optimal pH of the enzyme. After 20 min of incubation at 37 °C, the reaction was stopped by addition of sodium borate buffer pH 9.8. The *p*-nitrophenolate formed was measured by visible absorption spectroscopy at 405 nm.

The assays against α-Glucosidase II were carried out as described previously [3].

References

[1] Saul, R.; Chambers, J.P.; Molyneux, R.J.; Elbein, A.D. *Arch. Biochem. Biophys.* **1983**, *221*, 593–597.

[2] Brandi, A.; Cicchi, S.; Cordero, F.M.; Frignoli, R.; Goti, A.; Picasso, S.; Vogel, P. *J. Org. Chem.* **1995**, *60*, 6806–6812.

[3] Cumpstey, I.; Alonzi, D.S.; Butters, T.D. *Carbohydr. Res*. **2009**, *344*, 454–459.
