# Supplementary material for: Amine-linked diglycosides: Synthesis facilitated by the enhanced reactivity of allylic electrophiles, and glycosidase inhibition assays
Source: Beilstein J Org Chem. 2011 Aug 16;7:1115–23. doi: 10.3762/bjoc.7.128 (PMC3169339; doi:10.3762/bjoc.7.128)
Supplement: File 2 — 1H and 13C NMR spectra of compounds 9–17. [file Beilstein_J_Org_Chem-07-1115-s002.pdf]

Supporting Information
for

Amine-linked diglycosides: Synthesis facilitated by the enhanced reactivity of allylic electrophiles, and glycosidase inhibition assays

Ian Cumpstey[1,2,*], Jens Frigell[1], Elias Pershagen[1], Tashfeen Akhtar[1], Elena Moreno-Clavijo[3], Inmaculada Robina[3], Dominic S. Alonzi[4] and Terry D. Butters[4]

Address: [1]Department of Organic Chemistry, The Arrhenius Laboratory, Stockholm University, 106 91 Stockholm, Sweden, [2]Institut de Chimie des Substances Naturelles, Centre National de la Recherche Scientifique, 91198 Gif-sur-Yvette CEDEX, France, [3]Department of Organic Chemistry, Faculty of Chemistry, University of Seville, Prof. García González, 1, 41012 Seville, Spain and [4]Glycobiology Institute, Department of Biochemistry, Oxford University, South Parks Road, Oxford, OX1 3QU, England

Email: Ian Cumpstey - ian.cumpstey@sjc.oxon.org
* Corresponding author

^{1}H and ^{13}C NMR spectra of compounds 9–17

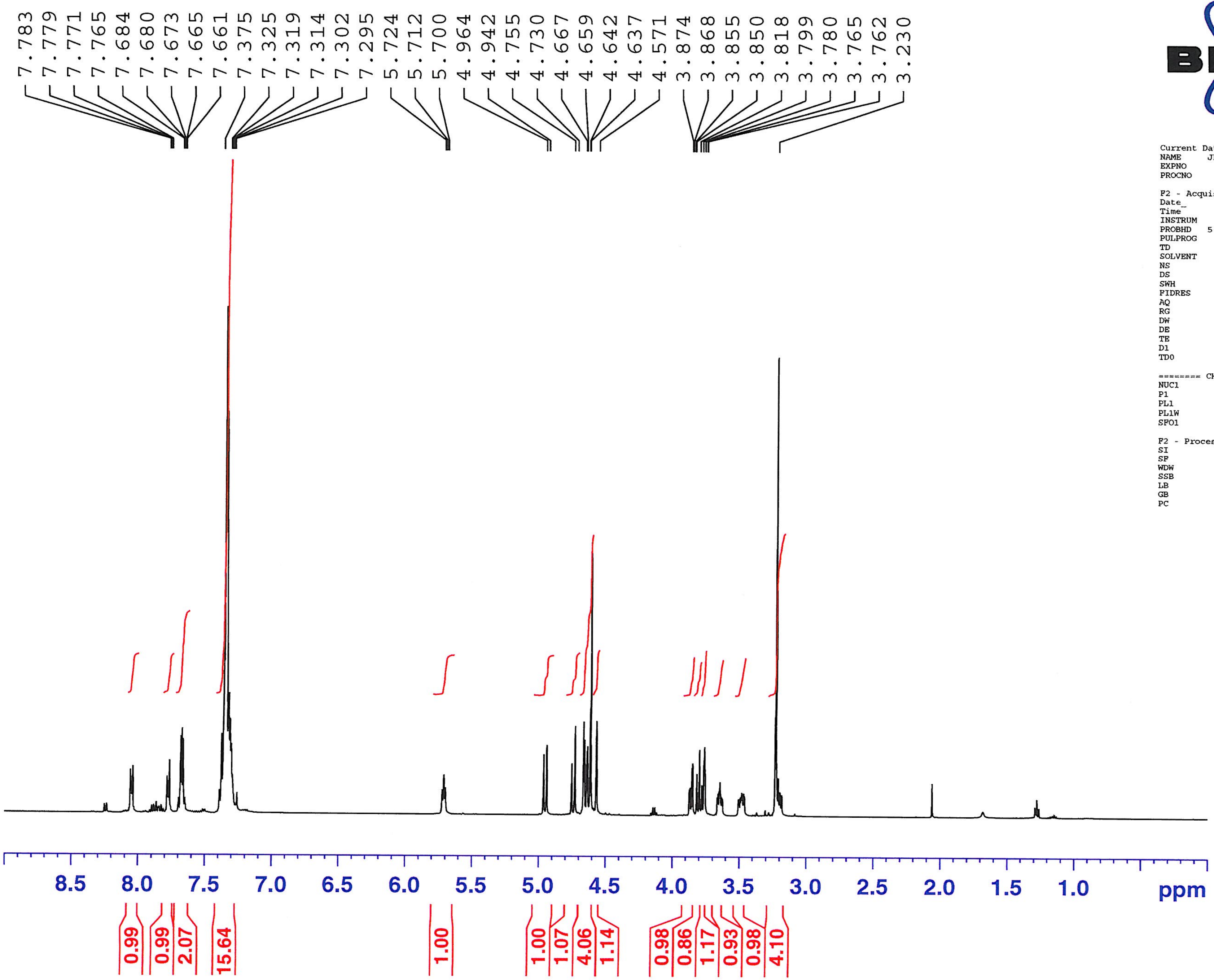


```
Current Data Parameters
NAME       JP-38-03-fr-4-28-nosylation
EXPNO                    10
PROCNO                   10

F2 - Acquisition Parameters
Date_              20100506
Time                   8.42
INSTRUM               spect
PROBHD     5 mm PABBO BB-
PULPROG                zg30
TD                    65536
SOLVENT               CDCl3
NS                       16
DS                        0
SWH               8012.820 Hz
FIDRES            0.122266 Hz
AQ               4.0894966 sec
RG                     90.5
DW                   62.400 usec
DE                     6.00 usec
TE                    298.2 K
D1               2.00000000 sec
TD0                       1

======== CHANNEL f1 ========
NUC1                     1H
P1                    11.80 usec
PL1                    2.00 dB
PL1W            15.76968765 W
SFO1            500.1325007 MHz

F2 - Processing parameters
SI                    32768
SF              500.1300133 MHz
WDW                      EM
SSB                       0
LB                     0.30 Hz
GB                        0
PC                     1.00
```

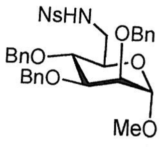

9

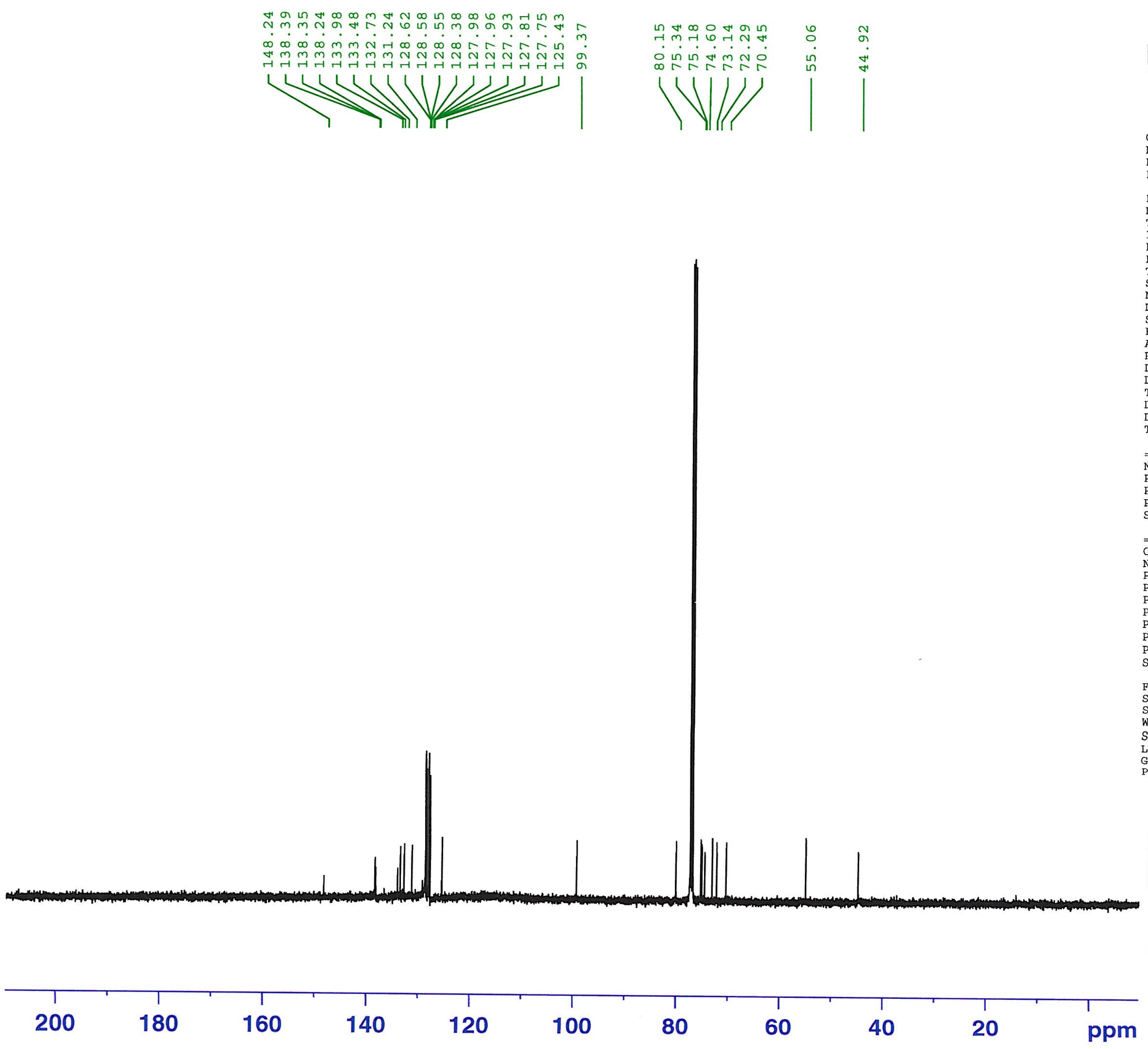

BRUKER

```
Current Data Parameters
NAME        JF-38-03-oct2010
EXPNO                     30
PROCNO                    10

F2 - Acquisition Parameters
Date_               20101012
Time                    4.27
INSTRUM                spect
PROBHD    5 mm PABBI 1H/
PULPROG               zgpg30
TD                     32768
SOLVENT                CDCl3
NS                       852
DS                         0
SWH             27573.529 Hz
FIDRES           0.841477 Hz
AQ              0.5942430 sec
RG                      2050
DW                18.133 usec
DE                 12.00 usec
TE                 298.2 K
D1            2.00000000 sec
D11           0.03000000 sec
TD0                        1

======== CHANNEL f1 ========
NUC1                     13C
P1                 14.75 usec
PL1                -0.30 dB
PL1W          89.89726257 W
SFO1         125.7703648 MHz

======== CHANNEL f2 ========
CPDPRG2              waltz16
NUC2                      1H
PCPD2              80.00 usec
PL2                 0.00 dB
PL12               22.50 dB
PL13               22.50 dB
PL2W          24.99327087 W
PL12W          0.14054748 W
PL13W          0.14054748 W
SFO2         500.1321306 MHz

F2 - Processing parameters
SI                     32768
SF           125.7577748 MHz
WDW                       EM
SSB                        0
LB                   1.00 Hz
GB                         0
PC                      1.40
```

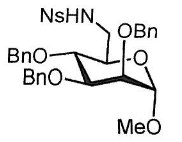

9

STANDARD PROTON

Pulse Sequence: s2pul

Solvent: CDCl3
Temp. 25.0 C / 298.1 K
Mercury-400BB "nmr4"

Relax. delay 1.000 sec
Pulse 39.2 degrees
Acq. time 2.733 sec
Width 5995.2 Hz
16 repetitions
OBSERVE H1, 399.9356487 MHz
DATA PROCESSING
Line broadening 0.1 Hz
FT size 65536
Total time 1 min, 2 sec

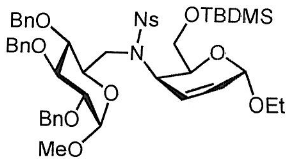

10

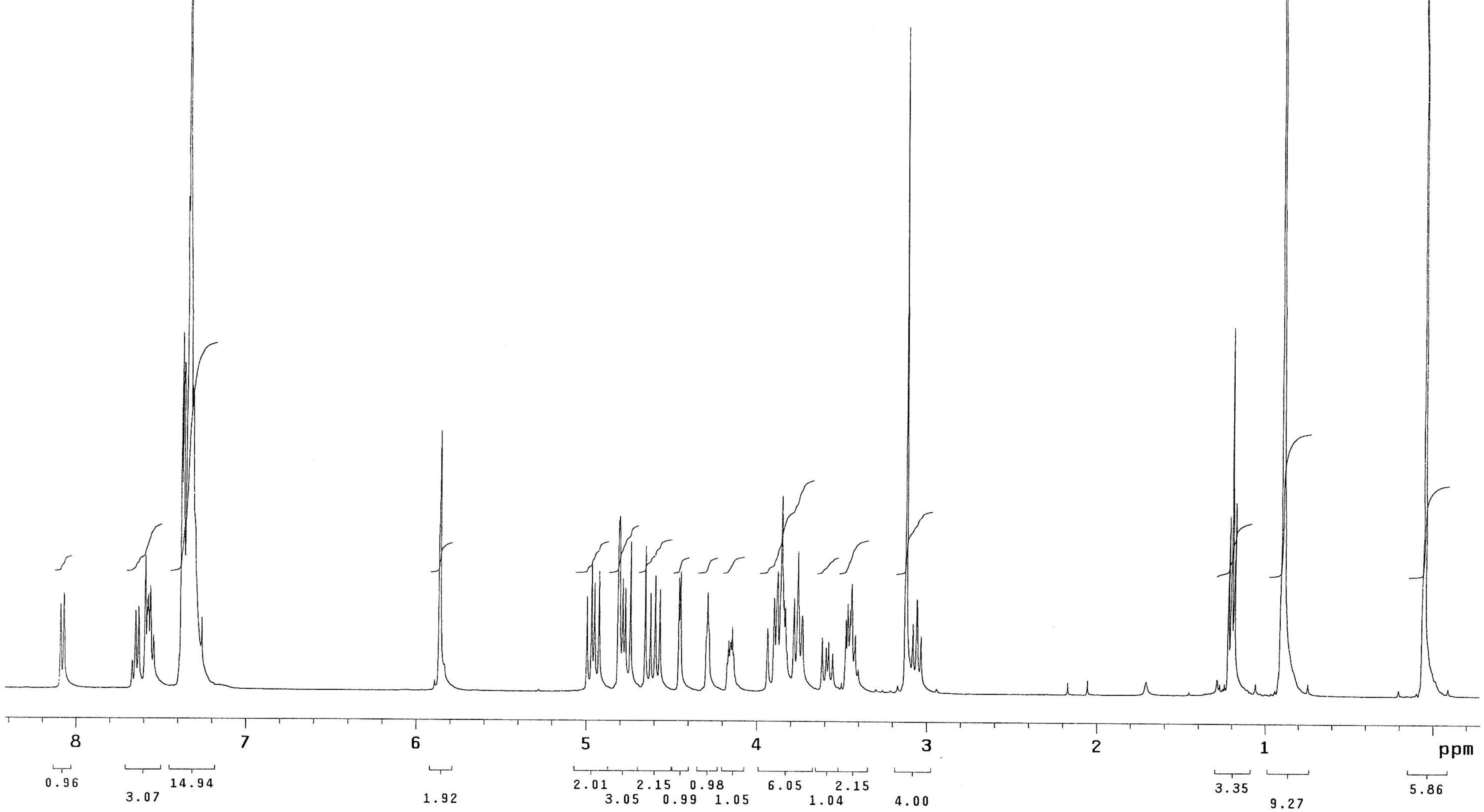

13C OBSERVE

Pulse Sequence: s2pul
Solvent: CDCl3
Temp. 25.0 C / 298.1 K
Mercury-400BB "nmr4"

Relax. delay 1.000 sec
Pulse 48.9 degrees
Acq. time 1.199 sec
Width 25000.0 Hz
148 repetitions
OBSERVE C13, 100.5638954 MHz
DECOUPLE H1, 399.9376154 MHz
Power 42 dB
continuously on
WALTZ-16 modulated
DATA PROCESSING
Line broadening 1.0 Hz
FT size 65536
Total time 42 min, 5 sec

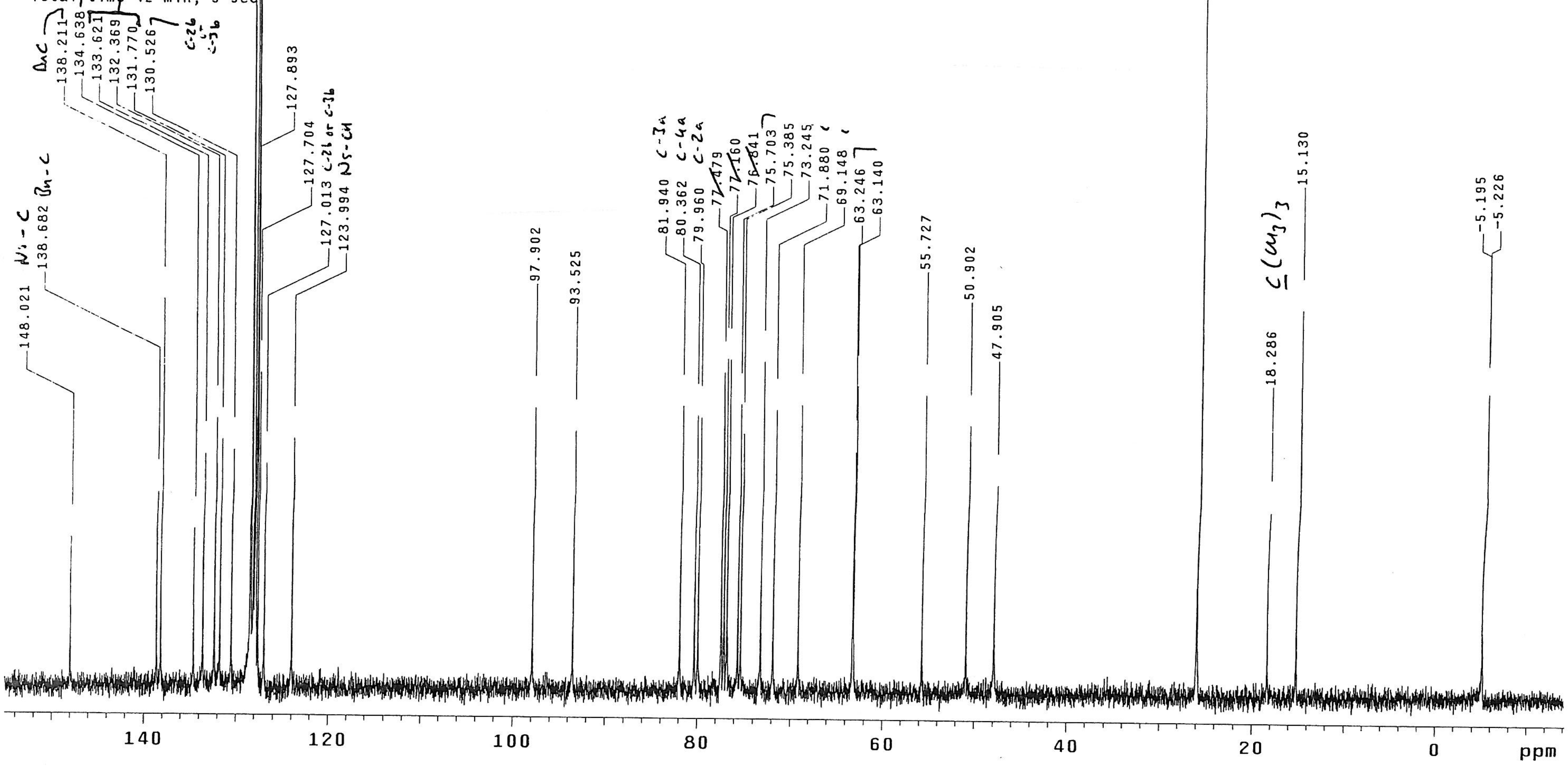

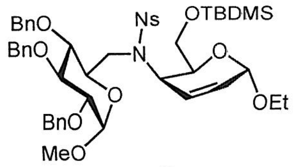

10

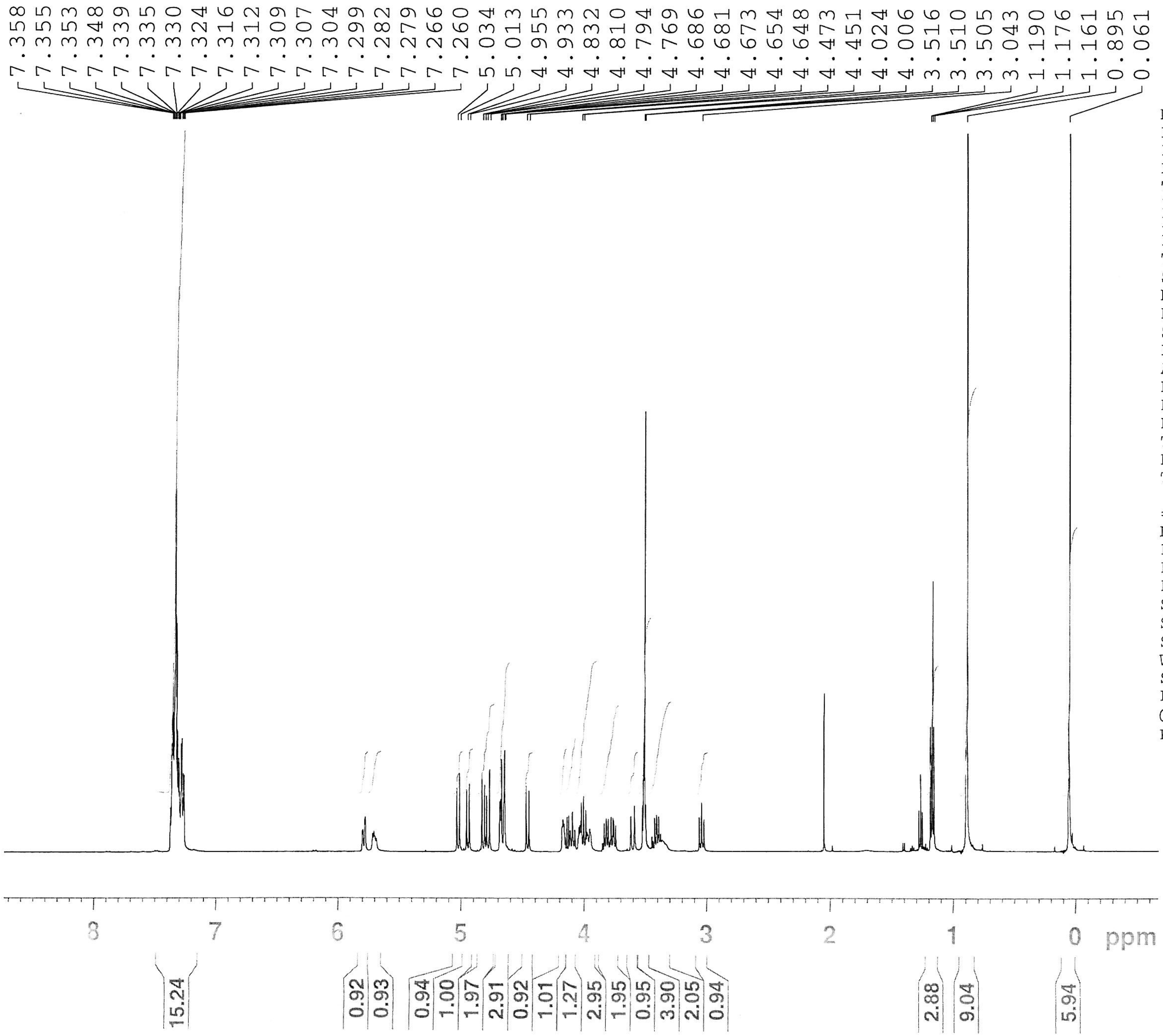

BRUKER

NAME	ic-29-16-12
EXPNO	1
PROCNO	1
Date_	20090315
Time	16.57
INSTRUM	spect
PROBHD	5 mm PABBO BB-
PULPROG	zg30
TD	65536
SOLVENT	CDCl3
NS	16
DS	0
SWH	8012.820 Hz
FIDRES	0.122266 Hz
AQ	4.0894966 sec
RG	36
DW	62.400 usec
DE	6.00 usec
TE	298.2 K
D1	2.00000000 sec
TD0	1

======== CHANNEL f1 ========

NUC1	1H
P1	11.80 usec
PL1	2.00 dB
PL1W	15.76968765 W
SFO1	500.1325006 MHz
SI	32768
SF	500.1300132 MHz
WDW	no
SSB	0
LB	0.00 Hz
GB	0
PC	1.00

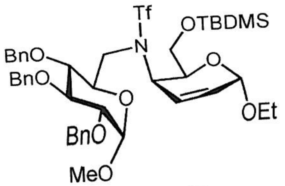

11

ic-29-16-12 2 1 /opt/home/cumpstey ic29

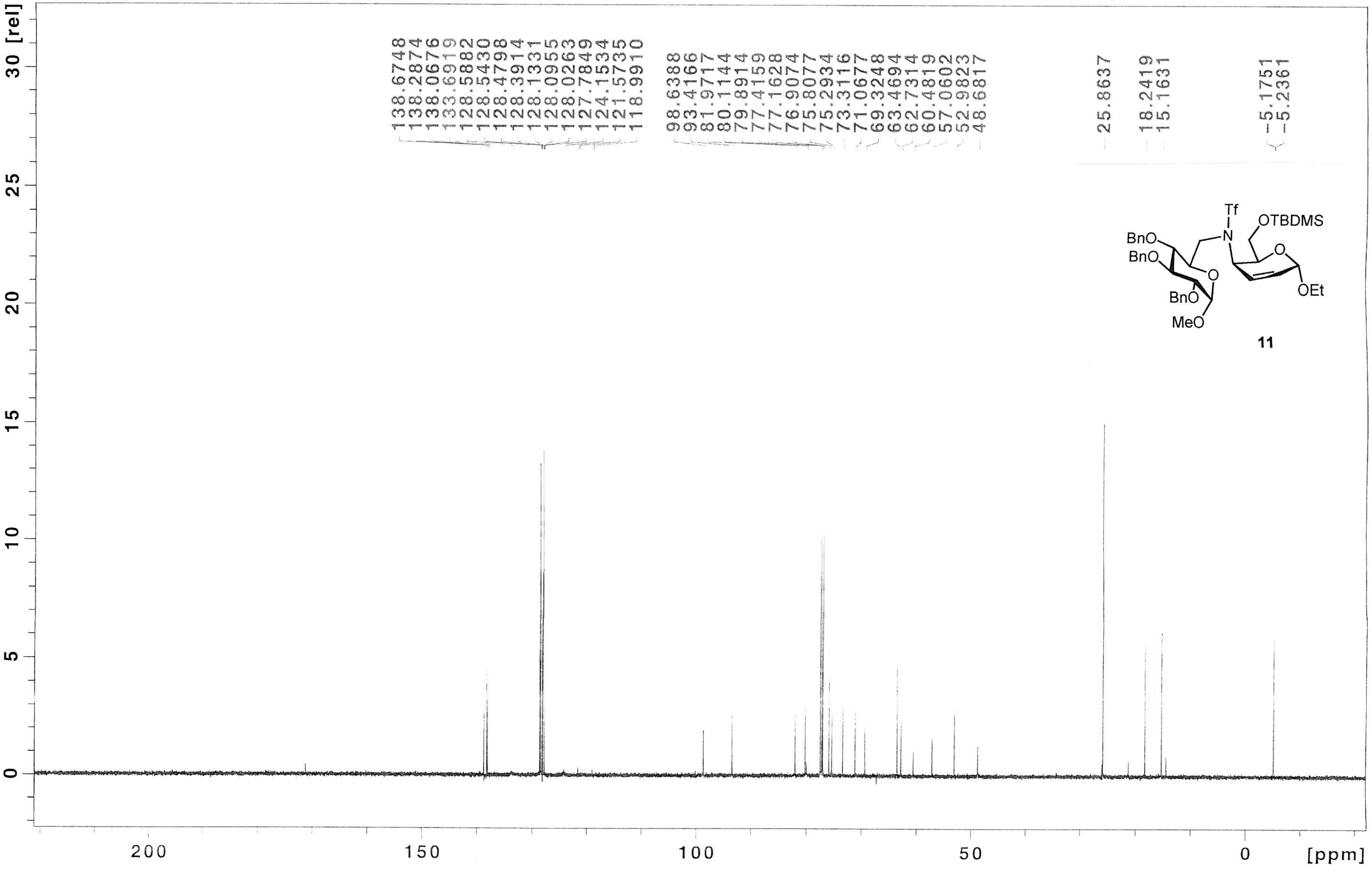

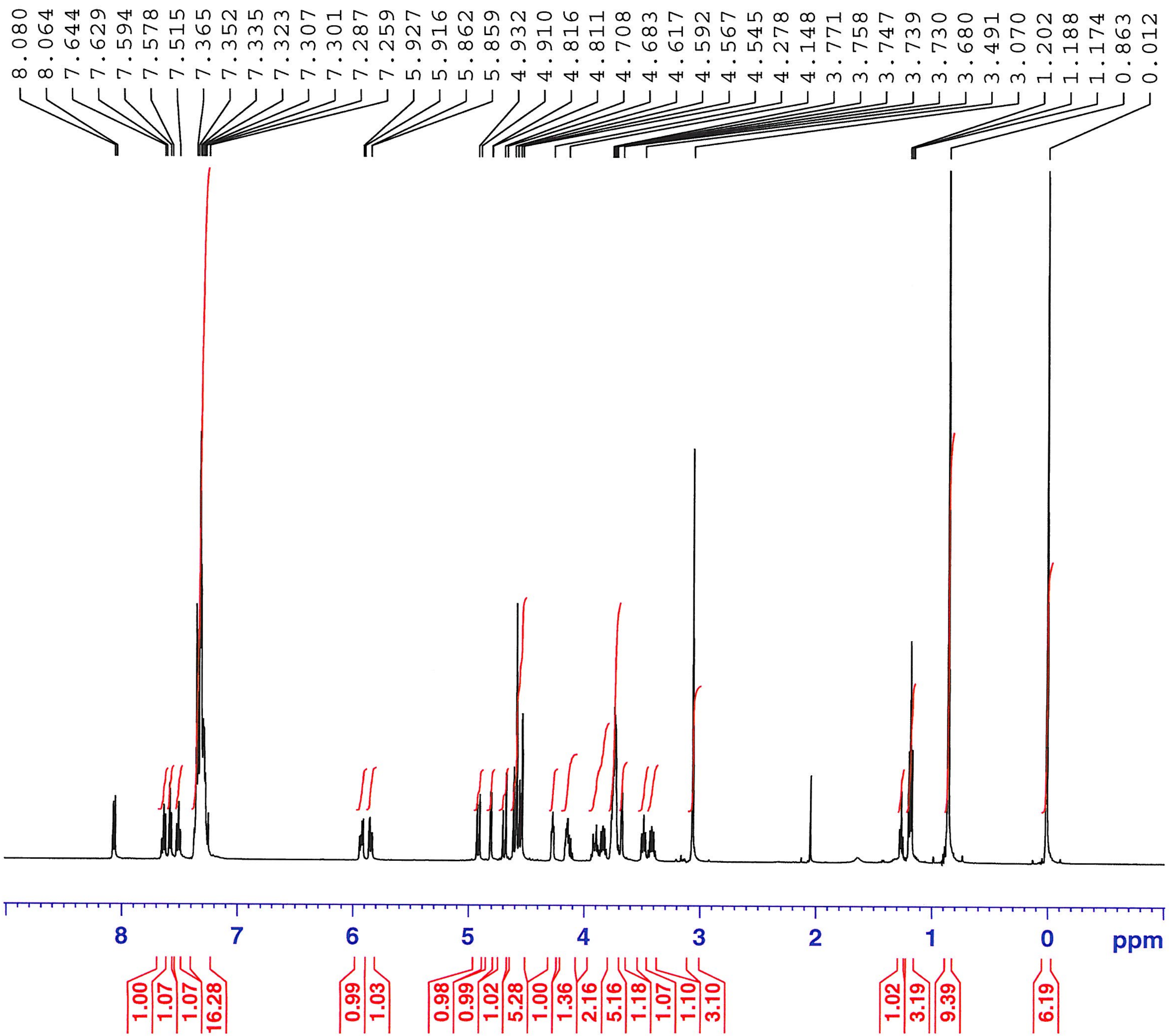

BRUKER

```
Current Data Parameters
NAME        JF-38-04-FL2-fr-24-37
EXPNO                  10
PROCNO                 10

F2 - Acquisition Parameters
Date_            20100511
Time                 5.33
INSTRUM             spect
PROBHD   5 mm PABBO BB-
PULPROG              zg30
TD                  65536
SOLVENT             CDCl3
NS                     16
DS                      0
SWH              8012.820 Hz
FIDRES           0.122266 Hz
AQ              4.0894966 sec
RG                   90.5
DW                 62.400 usec
DE                   6.00 usec
TE                  298.2 K
D1             2.00000000 sec
TD0                     1

======== CHANNEL f1 ========
NUC1                   1H
P1                  11.80 usec
PL1                  2.00 dB
PL1W          15.76968765 W
SFO1          500.1325007 MHz

F2 - Processing parameters
SI                  32768
SF            500.1300133 MHz
WDW                    EM
SSB                     0
LB                   0.30 Hz
GB                      0
PC                   1.00
```

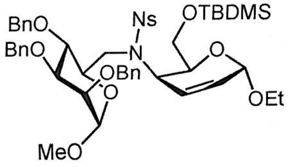

12

mitsunobu purified 13C

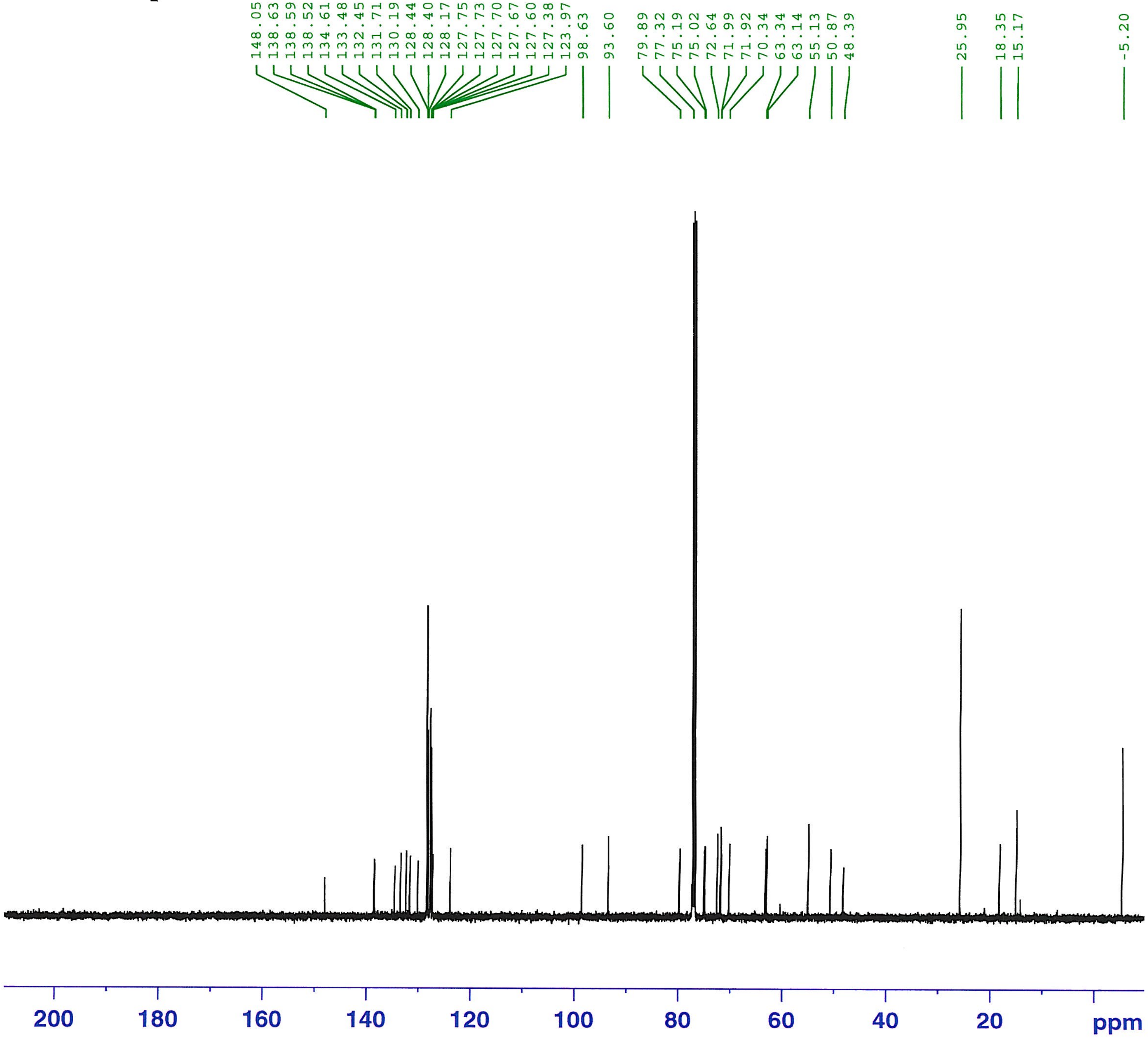


```
Current Data Parameters
NAME     JF-38-04-FL2-fr-24-37
EXPNO               30
PROCNO              10

F2 - Acquisition Parameters
Date_         20100511
Time              5.52
INSTRUM          spect
PROBHD   5 mm PABBO BB-
PULPROG        zgpg30
TD               32768
SOLVENT          CDCl3
NS                 279
DS                   0
SWH          27573.529 Hz
FIDRES        0.841477 Hz
AQ           0.5942430 sec
RG                2050
DW              18.133 usec
DE               12.00 usec
TE               298.2 K
D1          2.00000000 sec
D11         0.03000000 sec
TD0                  8

======== CHANNEL f1 ========
NUC1               13C
P1                7.50 usec
PL1               0.00 dB
PL1W       83.89700317 W
SFO1       125.7703648 MHz

======== CHANNEL f2 ========
CPDPRG2        waltz16
NUC2                1H
PCPD2            80.00 usec
PL2               2.00 dB
PL12             18.62 dB
PL13             21.00 dB
PL2W       15.76968765 W
PL12W       0.34341794 W
PL13W       0.19852860 W
SFO2       500.1321306 MHz

F2 - Processing parameters
SI               32768
SF         125.7577801 MHz
WDW                 EM
SSB                  0
LB                1.00 Hz
GB                   0
PC                1.40
```

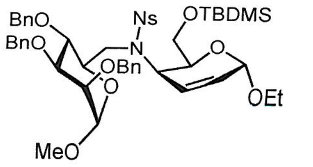

12

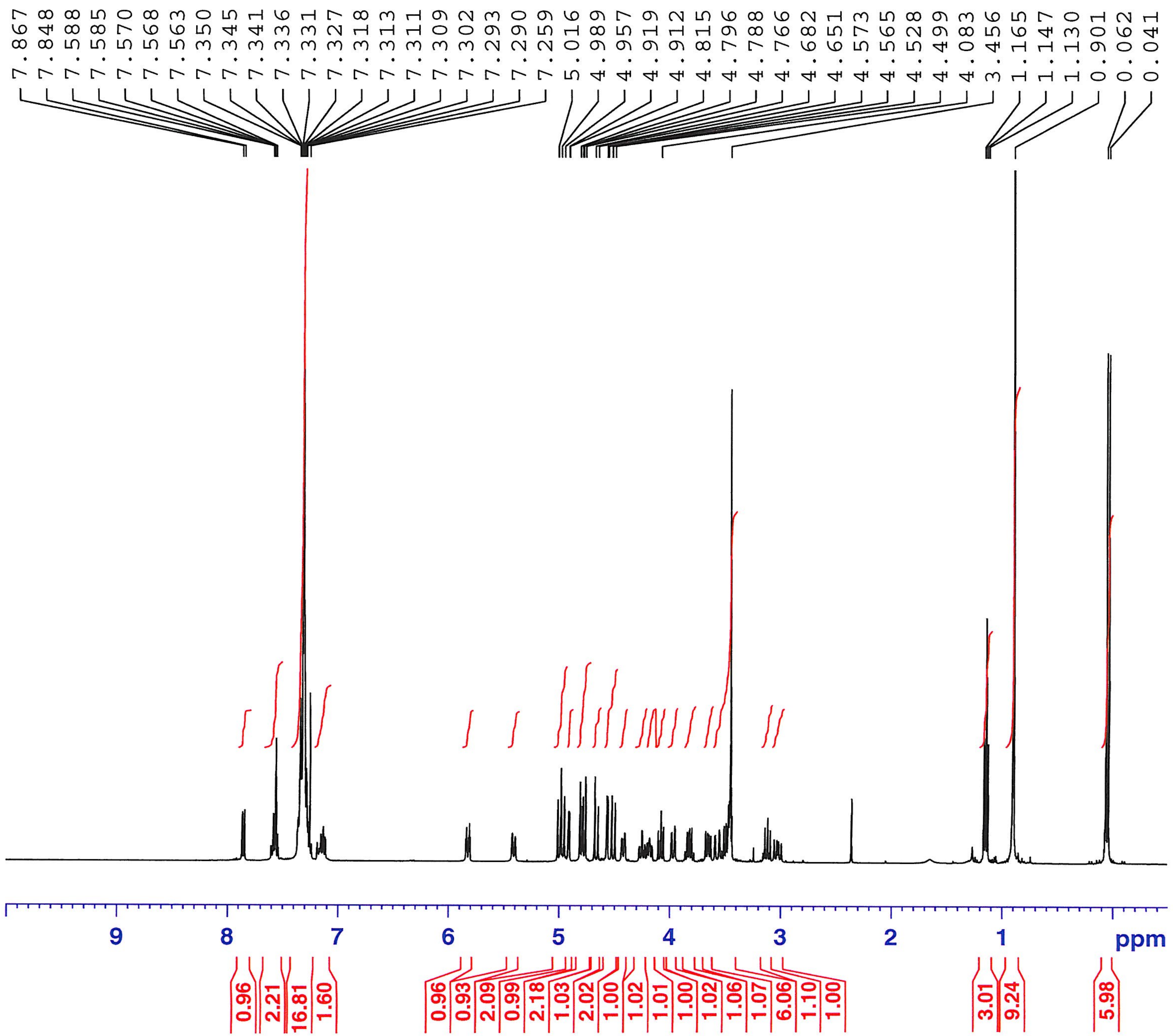


```
Current Data Parameters
NAME        JF-32-13-FL2-fr-4-7
EXPNO                    10
PROCNO                    1

F2 - Acquisition Parameters
Date_              20090821
Time                   4.07
INSTRUM               spect
PROBHD     5 mm PABBO BB-
PULPROG                zg30
TD                    65536
SOLVENT               CDCl3
NS                        8
DS                        0
SWH               8012.820 Hz
FIDRES            0.122266 Hz
AQ               4.0894966 sec
RG                     80.6
DW                   62.400 usec
DE                     6.00 usec
TE                    298.2 K
D1               1.00000000 sec
TD0                       1

======== CHANNEL f1 ========
NUC1                     1H
P1                    11.80 usec
PL1                    2.00 dB
PL1W            15.65874481 W
SFO1            500.1327507 MHz

F2 - Processing parameters
SI                    32768
SF              400.1300095 MHz
WDW                      EM
SSB                       0
LB                     0.30 Hz
GB                        0
PC                     1.00
```

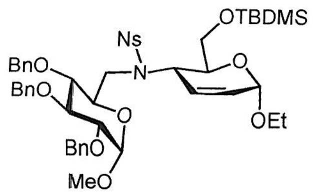

13

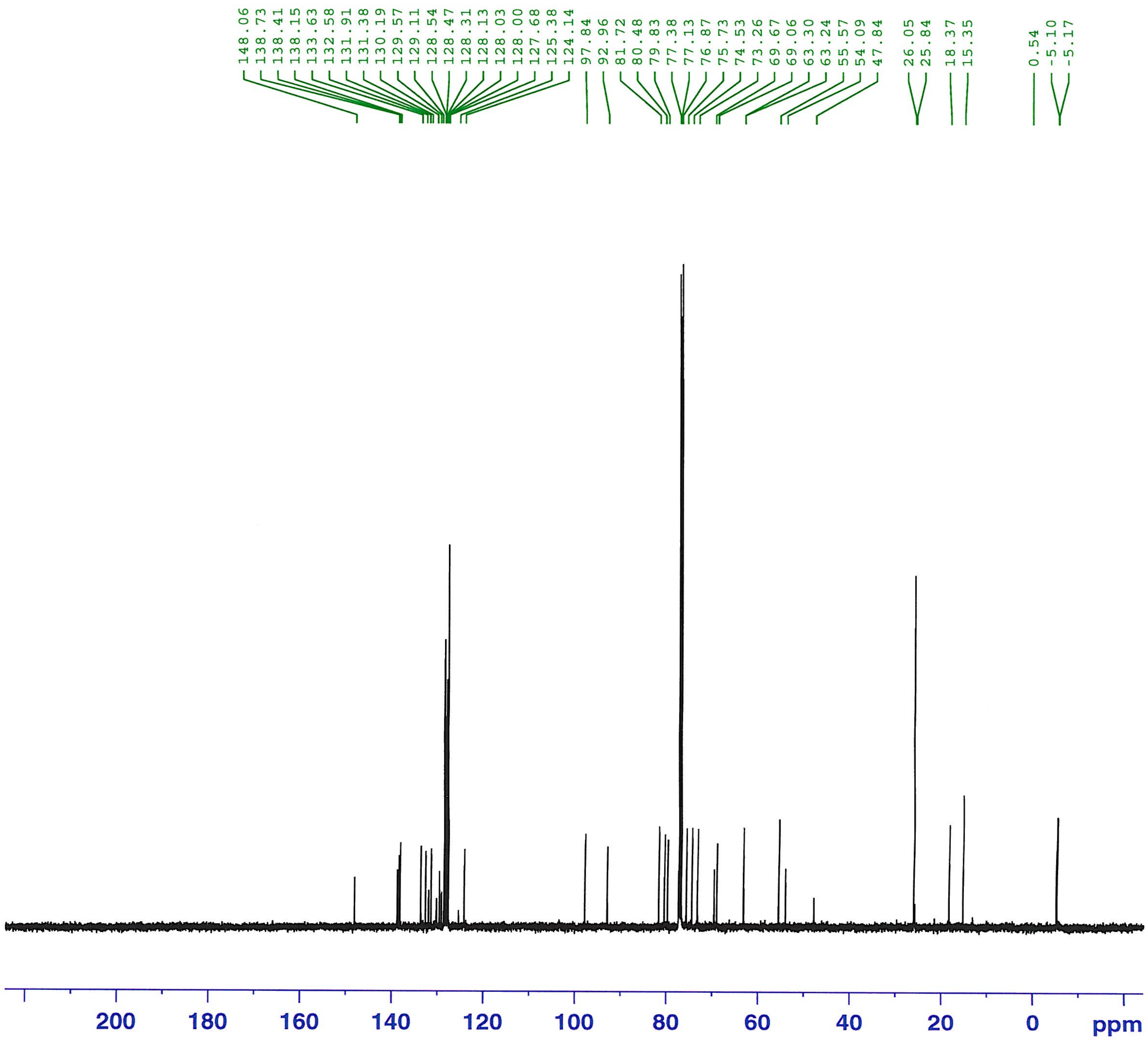


```
Current Data Parameters
NAME     JF-32-13-FL2-fr-4-7
EXPNO                     20
PROCNO                    20

F2 - Acquisition Parameters
Date_               20090821
Time                    4.16
INSTRUM                spect
PROBHD   5 mm PABBO BB-
PULPROG              zgpg30
TD                    65536
SOLVENT               CDCl3
NS                      477
DS                        2
SWH            31250.000 Hz
FIDRES          0.476837 Hz
AQ             1.0486259 sec
RG                    32800
DW               16.000 usec
DE                 6.00 usec
TE                298.2 K
D1           2.00000000 sec
D11          0.03000000 sec
TD0                       1

======== CHANNEL f1 ========
NUC1                    13C
P1                 7.50 usec
PL1                0.00 dB
PL1W        83.89700317 W
SFO1       125.7703648 MHz

======== CHANNEL f2 ========
CPDPRG2             waltz16
NUC2                     1H
PCPD2             80.00 usec
PL2                2.00 dB
PL12              18.62 dB
PL13              21.00 dB
PL2W        15.76968765 W
PL12W        0.34341794 W
PL13W        0.19852860 W
SFO2       500.1320000 MHz

F2 - Processing parameters
SI                    32768
SF         125.7577816 MHz
WDW                      EM
SSB                       0
LB                 1.00 Hz
GB                        0
PC                     1.40
```

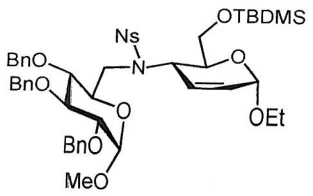

13

BRUKER

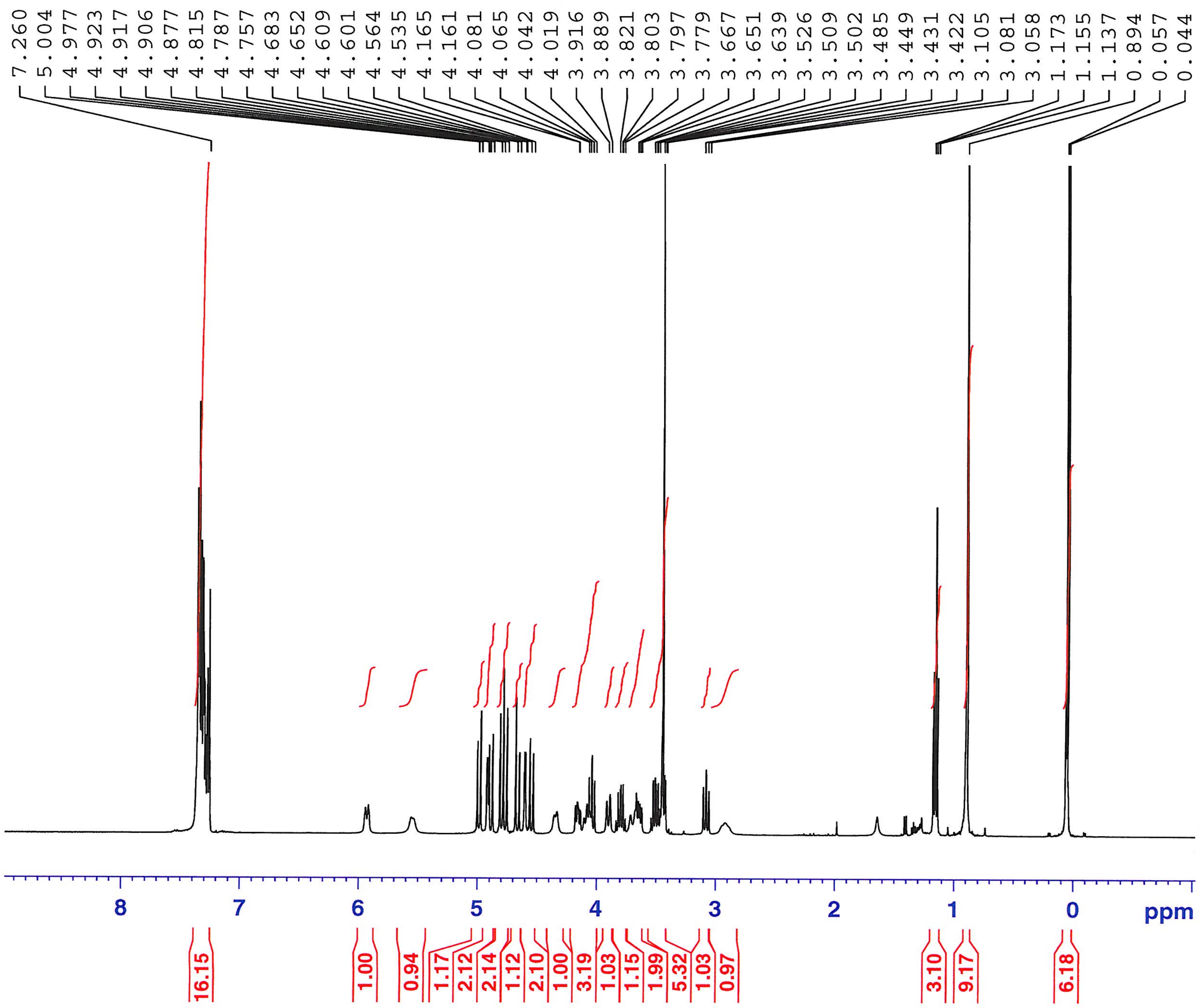

Current Data Parameters
NAME JF-32-12-fr-8-18-disacch
EXPNO 10
PROCNO 1

F2 - Acquisition Parameters
Date_ 20090818
Time 8.09
INSTRUM spect
PROBHD 5 mm PABBO BB-
PULPROG zg30
TD 65536
SOLVENT CDCl3
NS 16
DS 2
SWH 8223.685 Hz
FIDRES 0.125483 Hz
AQ 3.9846387 sec
RG 128
DW 60.800 usec
DE 6.50 usec
TE 297.1 K
D1 1.00000000 sec
TD0 1

======== CHANNEL f1 ========
NUC1 1H
P1 8.90 usec
PL1 -4.00 dB
PL1W 24.73352814 W
SFO1 400.1324710 MHz

F2 - Processing parameters
SI 32768
SF 400.1300093 MHz
WDW EM
SSB 0
LB 0.30 Hz
GB 0
PC 1.00

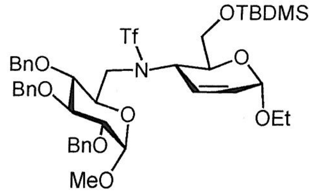

14

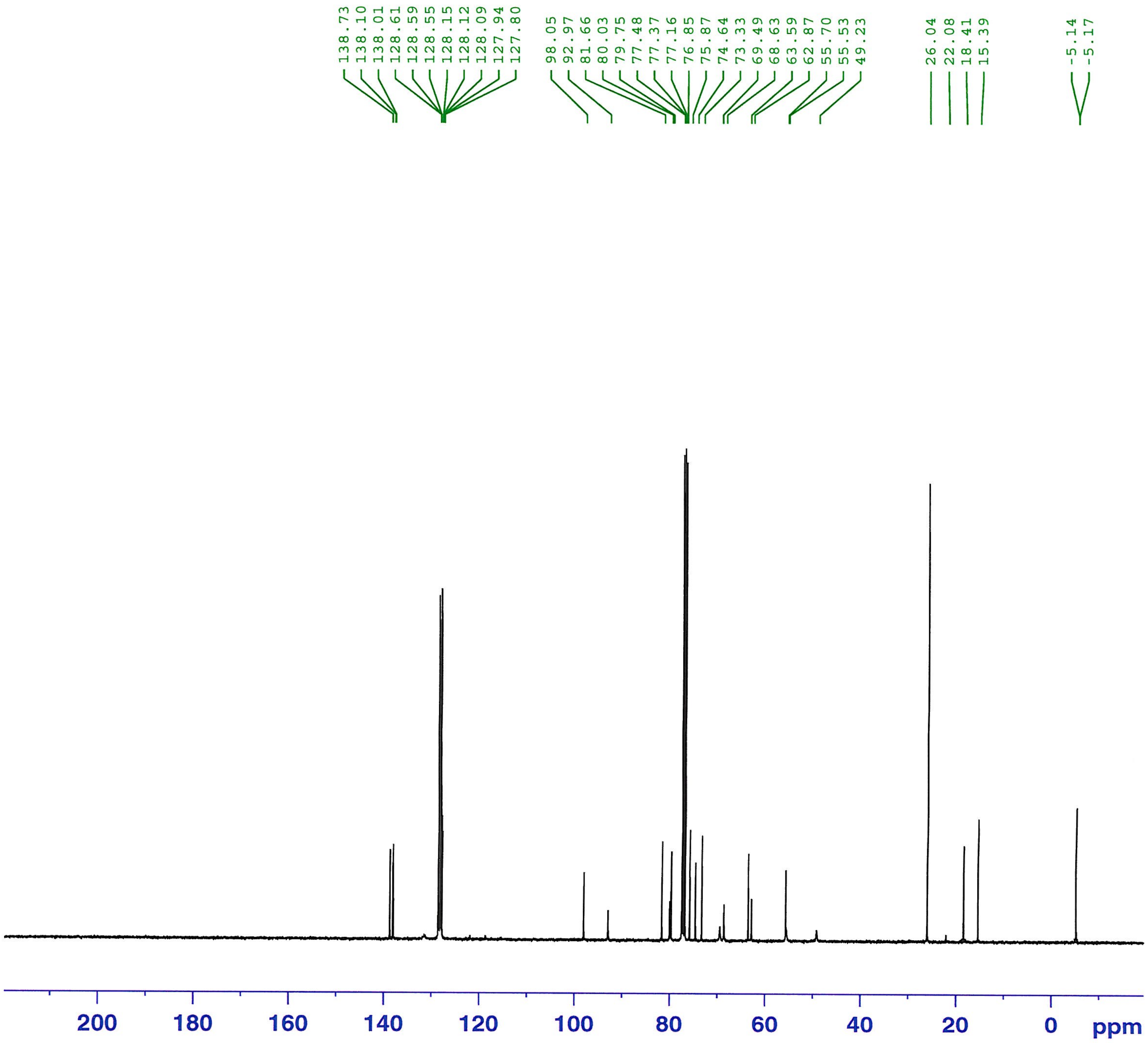

BRUKER

```
Current Data Parameters
NAME     JF-32-12-fr-8-18-disacch
EXPNO                   30
PROCNO                   1

F2 - Acquisition Parameters
Date_             20090818
Time                 21.46
INSTRUM              spect
PROBHD   5 mm PABBO BB-
PULPROG            zgpg30
TD                  65536
SOLVENT             CDCl3
NS                   3072
DS                      4
SWH             24038.461 Hz
FIDRES           0.366798 Hz
AQ              1.3631988 sec
RG                   2050
DW                 20.800 usec
DE                  51.93 usec
TE                  298.2 K
D1             2.00000000 sec
D11            0.03000000 sec
TD0                     1

======== CHANNEL f1 ========
NUC1                  13C
P1                   6.43 usec
PL1                 -3.00 dB
PL1W          69.66502380 W
SFO1          100.6228298 MHz

======== CHANNEL f2 ========
CPDPRG2           waltz16
NUC2                   1H
PCPD2               80.00 usec
PL2                 16.00 dB
PL12                15.00 dB
PL13                15.00 dB
PL2W           0.24733528 W
PL12W          0.31137666 W
PL13W          0.31137666 W
SFO2          400.1316005 MHz

F2 - Processing parameters
SI                  32768
SF            100.6127575 MHz
WDW                    EM
SSB                     0
LB                   1.00 Hz
GB                      0
PC                   1.40
```

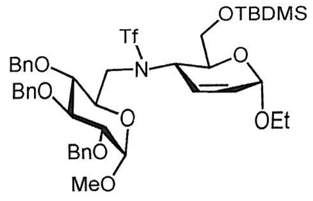

14

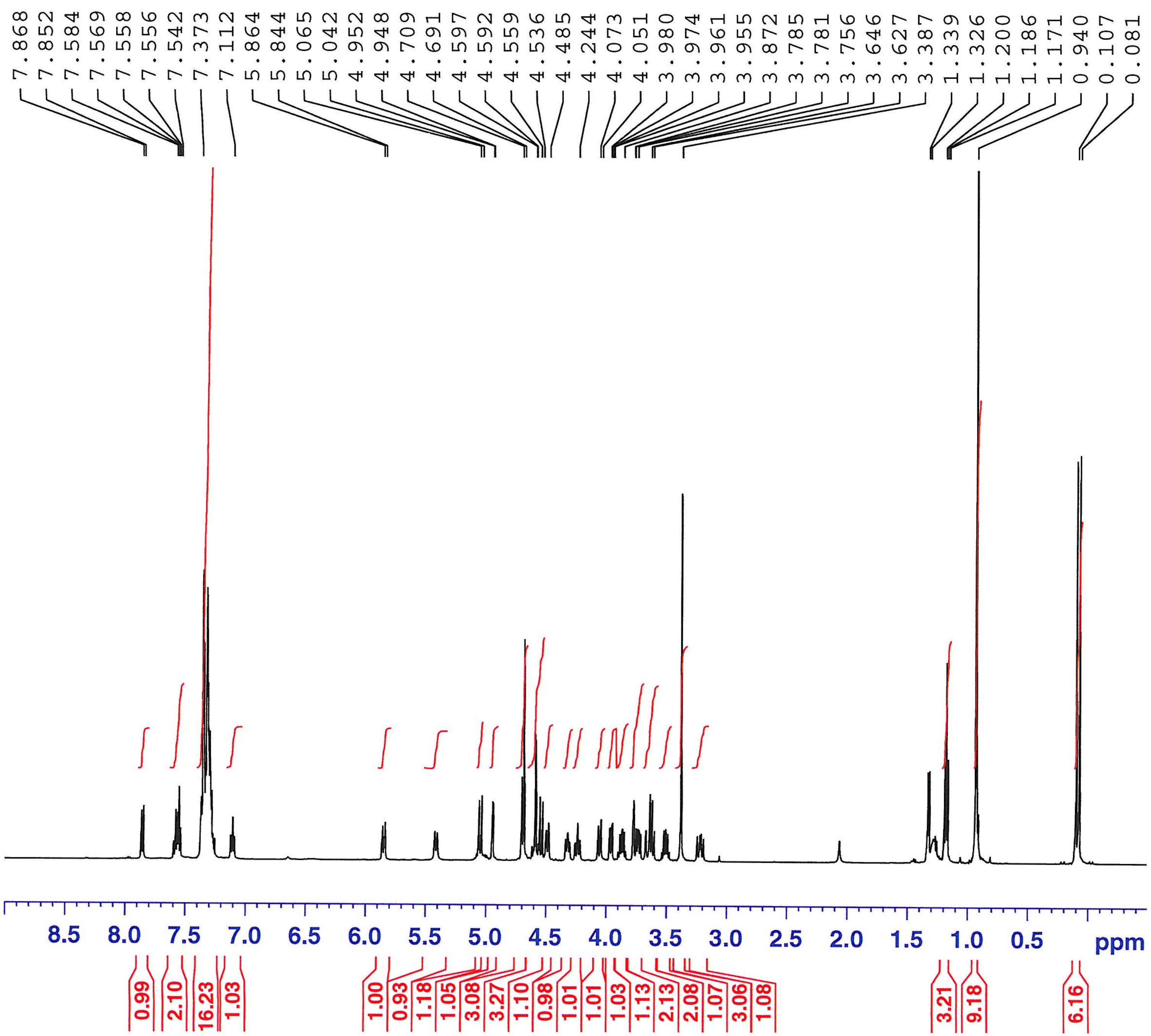

Current Data Parameters

NAME	JF-38-05-fr-48-61
EXPNO	10
PROCNO	10

F2 - Acquisition Parameters

Date_	20100510
Time	1.22
INSTRUM	spect
PROBHD	5 mm PABBO BB-
PULPROG	zg30
TD	65536
SOLVENT	CDCl3
NS	16
DS	0
SWH	8012.820 Hz
FIDRES	0.122266 Hz
AQ	4.0894966 sec
RG	36
DW	62.400 usec
DE	6.00 usec
TE	298.2 K
D1	2.00000000 sec
TD0	1

======== CHANNEL f1 ========

NUC1	1H
P1	11.80 usec
PL1	2.00 dB
PL1W	15.76968765 W
SFO1	500.1325007 MHz

F2 - Processing parameters

SI	32768
SF	500.1300130 MHz
WDW	EM
SSB	0
LB	0.30 Hz
GB	0
PC	1.00

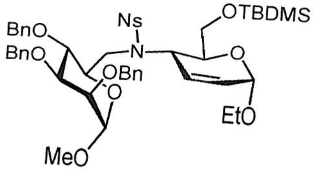

15

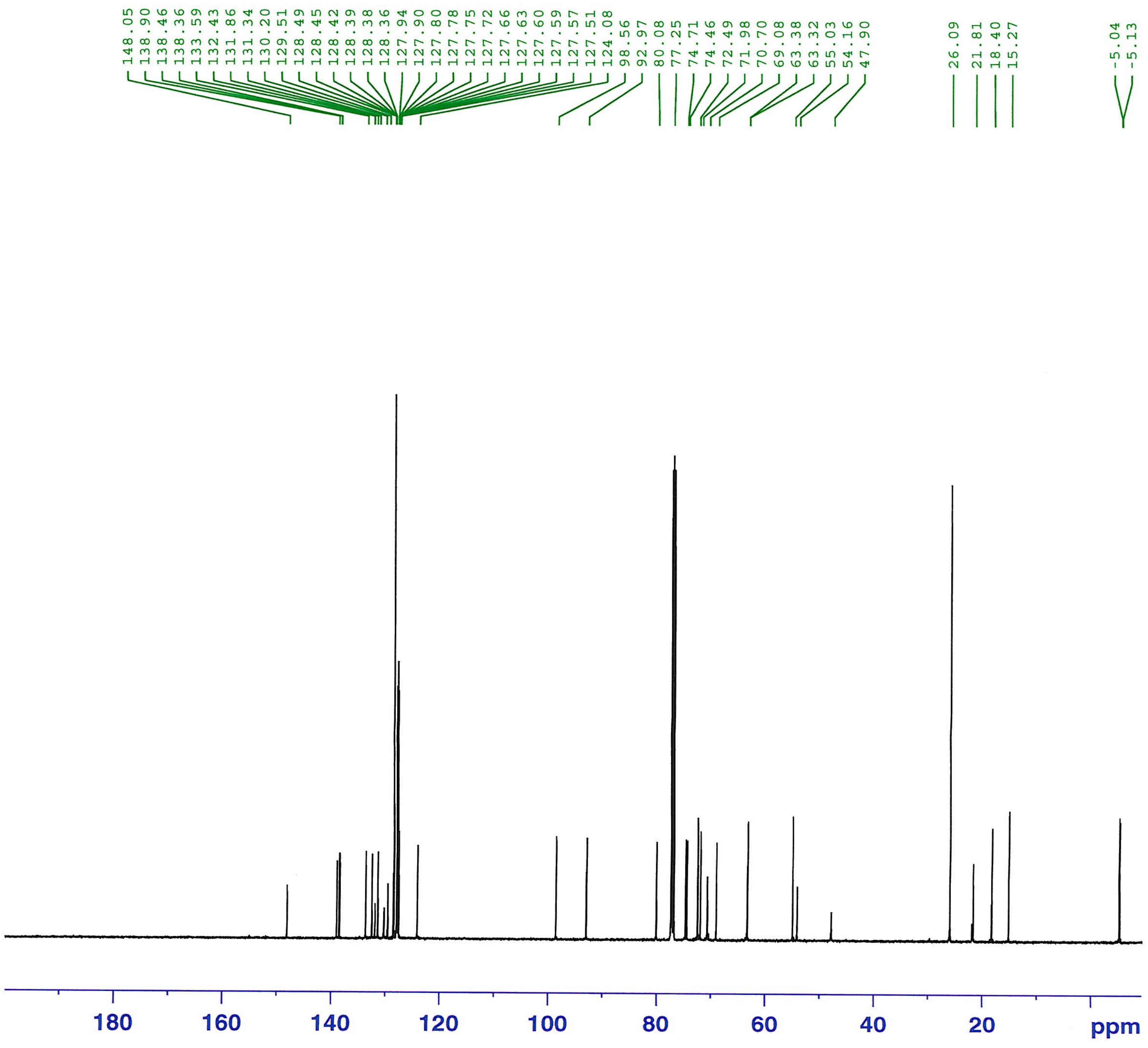

BRUKER

```
Current Data Parameters
NAME     JF-38-05-fr-48-61
EXPNO                   30
PROCNO                  30

F2 - Acquisition Parameters
Date_           20100510
Time                1.55
INSTRUM            spect
PROBHD   5 mm PABBO BB-
PULPROG          zgpg30
TD                32768
SOLVENT           CDCl3
NS                 2048
DS                    0
SWH           27573.529 Hz
FIDRES         0.841477 Hz
AQ            0.5942430 sec
RG                 2050
DW               18.133 usec
DE                12.00 usec
TE                298.2 K
D1           2.00000000 sec
D11          0.03000000 sec
TD0                   1

======== CHANNEL f1 ========
NUC1                13C
P1                 7.50 usec
PL1                0.00 dB
PL1W        83.89700317 W
SFO1        125.7703648 MHz

======== CHANNEL f2 ========
CPDPRG2          waltz16
NUC2                  1H
PCPD2              80.00 usec
PL2                 2.00 dB
PL12               18.62 dB
PL13               21.00 dB
PL2W         15.76968765 W
PL12W         0.34341794 W
PL13W         0.19852860 W
SFO2         500.1321306 MHz

F2 - Processing parameters
SI                 32768
SF           125.7577816 MHz
WDW                   EM
SSB                    0
LB                  1.00 Hz
GB                     0
PC                  1.40
```

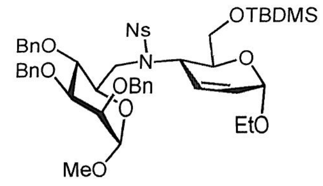

15

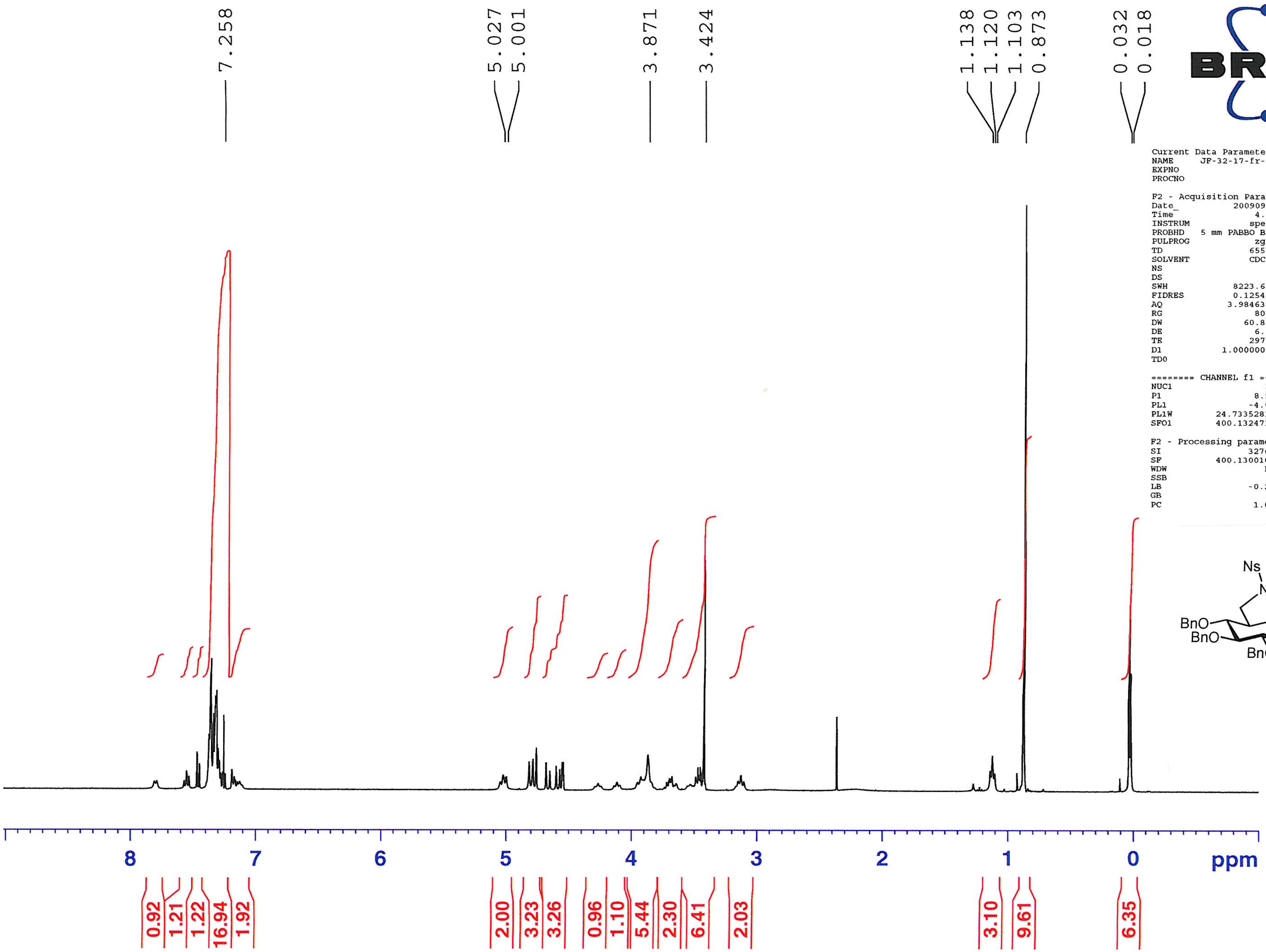


```
Current Data Parameters
NAME      JF-32-17-fr-21-32-dihydrox
EXPNO                 10
PROCNO                 1

F2 - Acquisition Parameters
Date_           20090918
Time                4.12
INSTRUM            spect
PROBHD   5 mm PABBO BB-
PULPROG             zg30
TD                 65536
SOLVENT            CDCl3
NS                    16
DS                     2
SWH             8223.685 Hz
FIDRES          0.125483 Hz
AQ             3.9846387 sec
RG                  80.6
DW                60.800 usec
DE                  6.50 usec
TE                 297.2 K
D1            1.00000000 sec
TD0                    1

======== CHANNEL f1 ========
NUC1                  1H
P1                  8.90 usec
PL1                -4.00 dB
PL1W         24.73352814 W
SFO1         400.1324710 MHz

F2 - Processing parameters
SI                 32768
SF           400.1300102 MHz
WDW                   EM
SSB                    0
LB                 -0.20 Hz
GB                     0
PC                  1.00
```

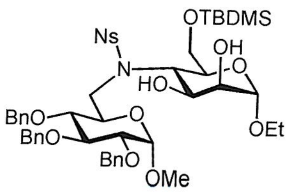

16

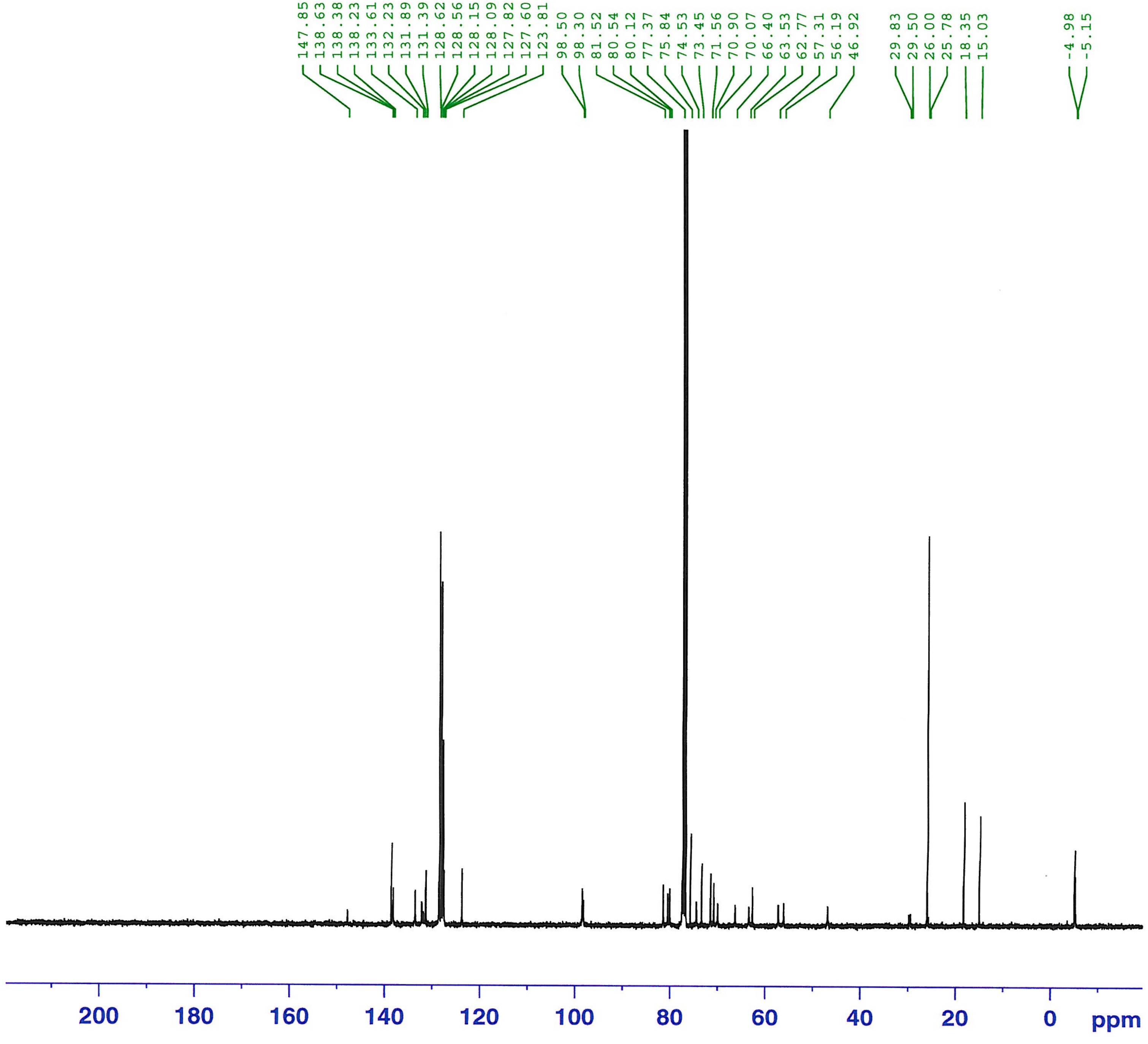

BRUKER

```
Current Data Parameters
NAME      JF-32-22-october2010
EXPNO                   20
PROCNO                   1

F2 - Acquisition Parameters
Date_             20101005
Time                 15.13
INSTRUM              spect
PROBHD    5 mm PABBO BB-
PULPROG            zgpg30
TD                   65536
SOLVENT              CDCl3
NS                    3072
DS                       4
SWH              24038.461 Hz
FIDRES            0.366798 Hz
AQ               1.3631988 sec
RG                    2050
DW                  20.800 usec
DE                   51.93 usec
TE                   298.0 K
D1              2.00000000 sec
D11             0.03000000 sec
TD0                      1

======== CHANNEL f1 ========
NUC1                   13C
P1                    6.43 usec
PL1                  -3.00 dB
PL1W           69.66502380 W
SFO1           100.6228298 MHz

======== CHANNEL f2 ========
CPDPRG2            waltz16
NUC2                    1H
PCPD2                80.00 usec
PL2                  16.00 dB
PL12                 15.00 dB
PL13                 15.00 dB
PL2W            0.24733528 W
PL12W           0.31137666 W
PL13W           0.31137666 W
SFO2           400.1316005 MHz

F2 - Processing parameters
SI                   32768
SF             100.6127572 MHz
WDW                     EM
SSB                      0
LB                    1.00 Hz
GB                       0
PC                    1.40
```

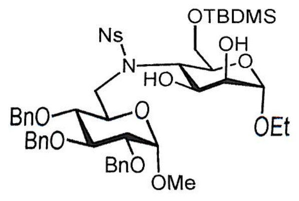

16

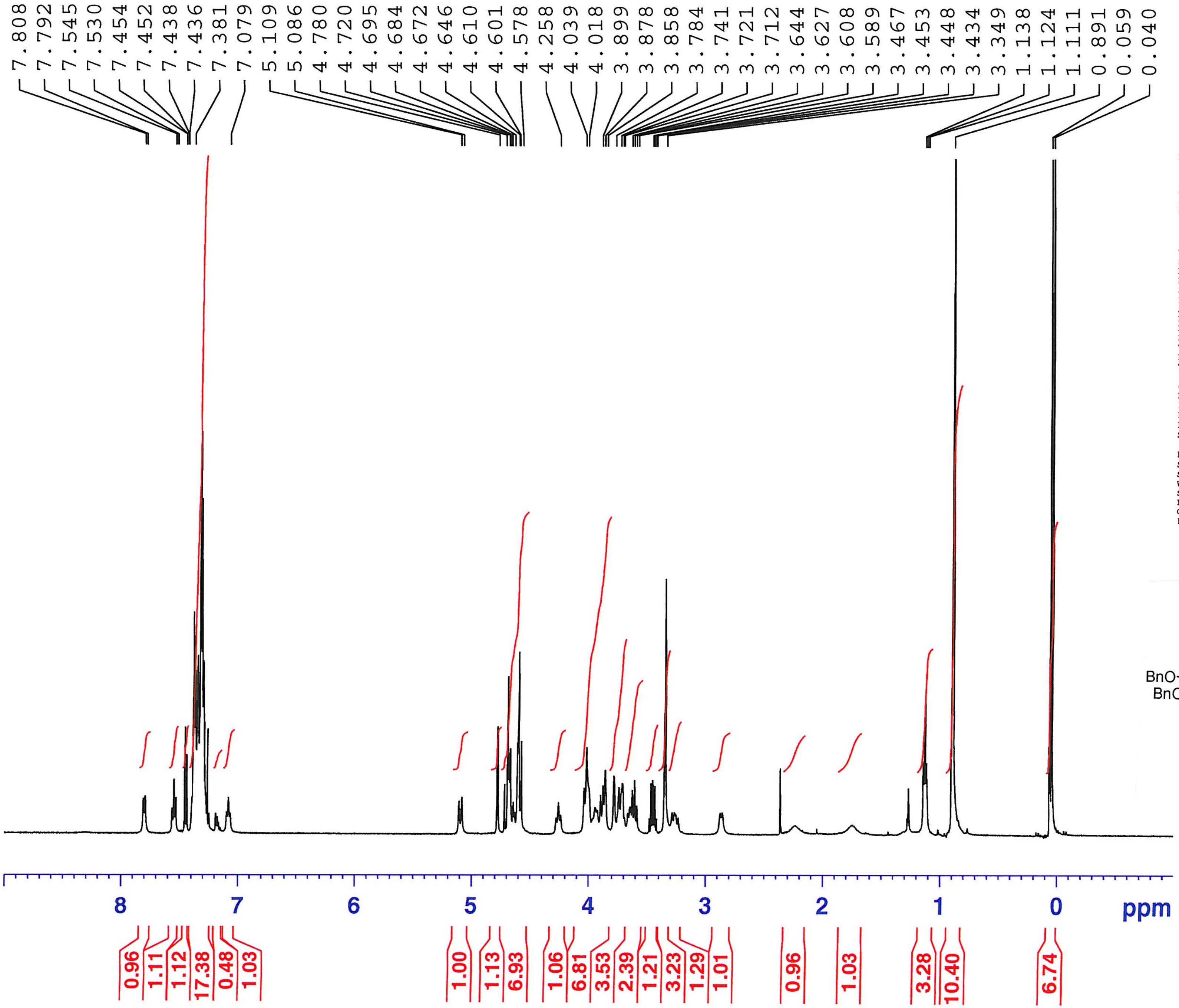

BRUKER

```
Current Data Parameters
NAME        JF-38-06-fr-18-26-dihydrox
EXPNO                         10
PROCNO                        10

F2 - Acquisition Parameters
Date_                   20100519
Time                        5.18
INSTRUM                    spect
PROBHD      5 mm PABBO BB-
PULPROG                     zg30
TD                         65536
SOLVENT                    CDCl3
NS                            16
DS                             0
SWH                     8012.820 Hz
FIDRES                  0.122266 Hz
AQ                     4.0894966 sec
RG                          90.5
DW                        62.400 usec
DE                          6.00 usec
TE                         298.0 K
D1                    2.00000000 sec
TD0                            1

======== CHANNEL f1 ========
NUC1                          1H
P1                         11.80 usec
PL1                         2.00 dB
PL1W                 15.76968765 W
SFO1                 500.1325007 MHz

F2 - Processing parameters
SI                         32768
SF                   500.1300131 MHz
WDW                           EM
SSB                            0
LB                         -0.10 Hz
GB                             0
PC                          1.00
```

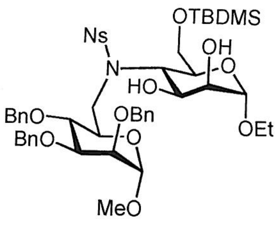

17

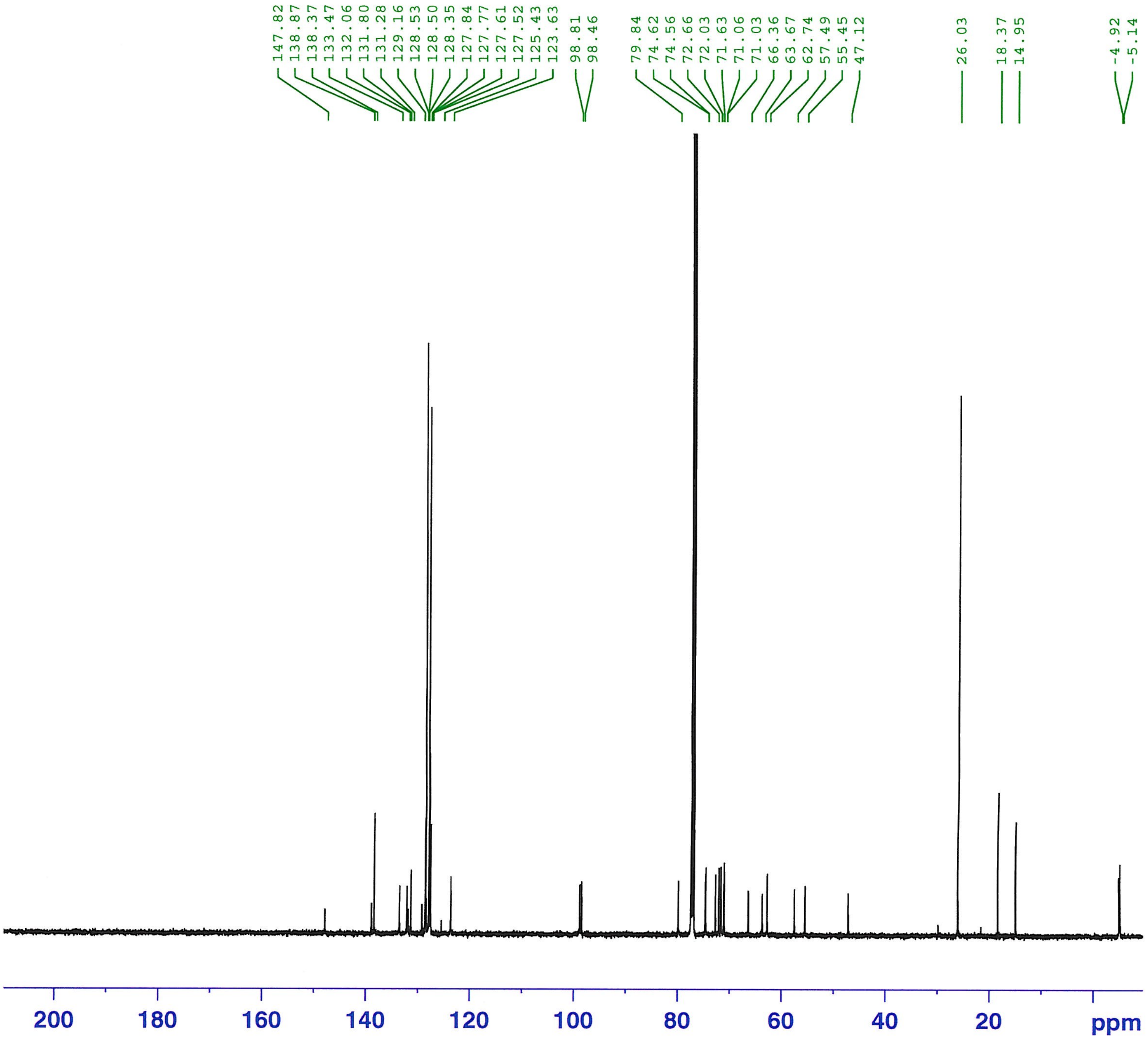

BRUKER

```
Current Data Parameters
NAME      JF-38-06-fr-18-26-dihydrox
EXPNO                 30
PROCNO                10

F2 - Acquisition Parameters
Date_            20100519
Time                 5.41
INSTRUM             spect
PROBHD   5 mm PABBO BB-
PULPROG           zgpg30
TD                 32768
SOLVENT            CDCl3
NS                  2048
DS                     0
SWH            27573.529 Hz
FIDRES          0.841477 Hz
AQ             0.5942430 sec
RG                  2050
DW                18.133 usec
DE                 12.00 usec
TE                 298.0 K
D1            2.00000000 sec
D11           0.03000000 sec
TD0                    1

======== CHANNEL f1 ========
NUC1                 13C
P1                  7.50 usec
PL1                 0.00 dB
PL1W         83.89700317 W
SFO1         125.7703648 MHz

======== CHANNEL f2 ========
CPDPRG2          waltz16
NUC2                  1H
PCPD2              80.00 usec
PL2                 2.00 dB
PL12               18.62 dB
PL13               21.00 dB
PL2W         15.76968765 W
PL12W         0.34341794 W
PL13W         0.19852860 W
SFO2         500.1321306 MHz

F2 - Processing parameters
SI                 32768
SF           125.7577754 MHz
WDW                   EM
SSB                    0
LB                  1.00 Hz
GB                     0
PC                  1.40
```

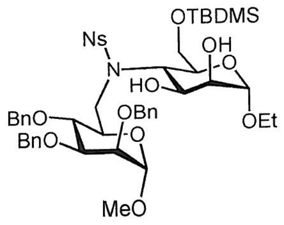

17
