# Supplementary material for: Amine-linked diglycosides: Synthesis facilitated by the enhanced reactivity of allylic electrophiles, and glycosidase inhibition assays
Source: Beilstein J Org Chem. 2011 Aug 16;7:1115–23. doi: 10.3762/bjoc.7.128 (PMC3169339; doi:10.3762/bjoc.7.128)
Supplement: File 3 — 1H and 13C NMR spectra of compounds 18–27. [file Beilstein_J_Org_Chem-07-1115-s003.pdf]

Supporting Information

for

Amine-linked diglycosides: Synthesis facilitated by the enhanced reactivity of allylic electrophiles, and glycosidase inhibition assays

Ian Cumpstey[1,2,*], Jens Frigell[1], Elias Pershagen[1], Tashfeen Akhtar[1], Elena Moreno-Clavijo[3], Inmaculada Robina[3], Dominic S. Alonzi[4] and Terry D. Butters[4]

Address: [1]Department of Organic Chemistry, The Arrhenius Laboratory, Stockholm University, 106 91 Stockholm, Sweden, [2]Institut de Chimie des Substances Naturelles, Centre National de la Recherche Scientifique, 91198 Gif-sur-Yvette CEDEX, France, [3]Department of Organic Chemistry, Faculty of Chemistry, University of Seville, Prof. García González, 1, 41012 Seville, Spain and [4]Glycobiology Institute, Department of Biochemistry, Oxford University, South Parks Road, Oxford, OX1 3QU, England

Email: Ian Cumpstey - ian.cumpstey@sjc.oxon.org

* Corresponding author

^{1}H and ^{13}C NMR spectra of compounds 18–27

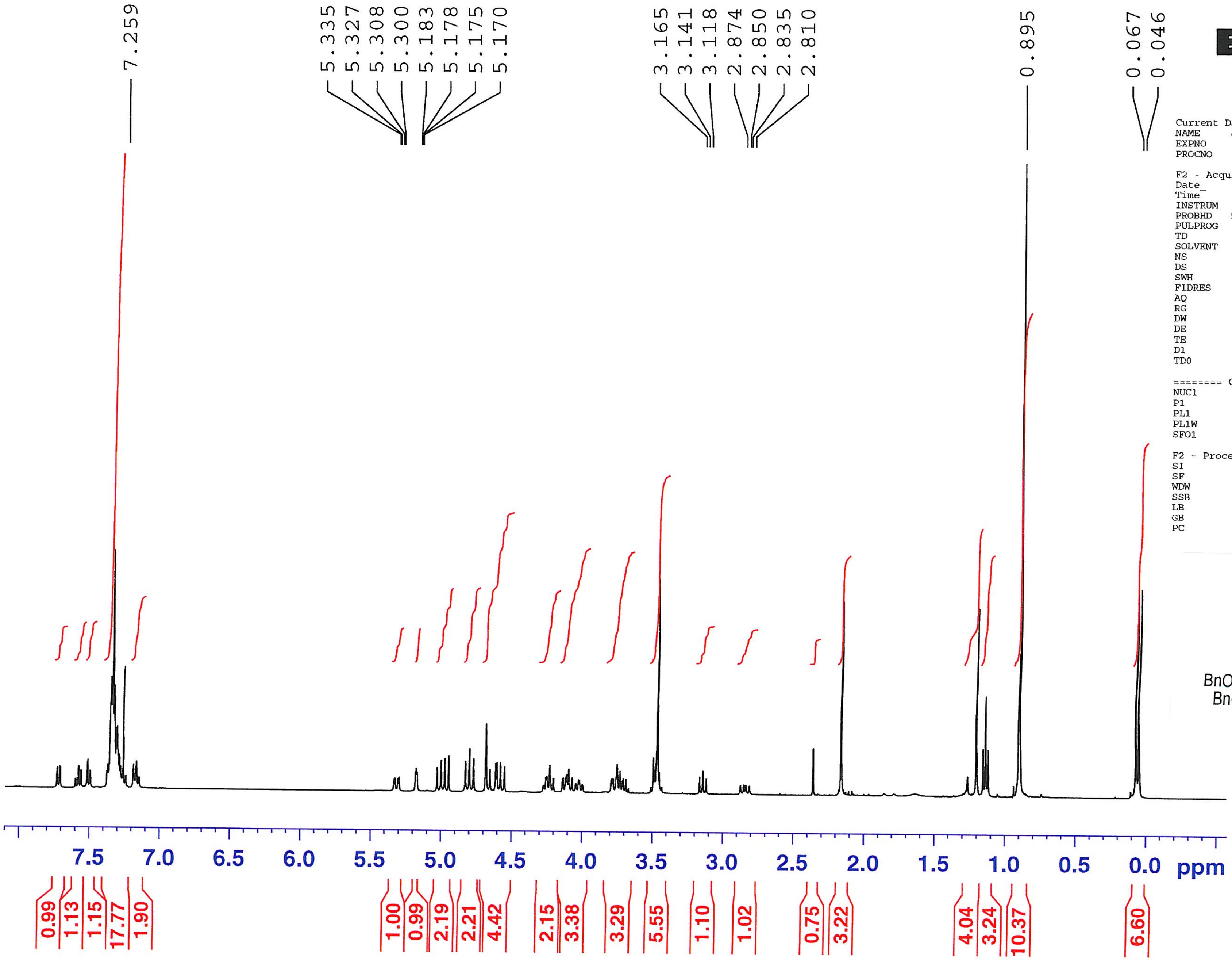


```
Current Data Parameters
NAME      JF-32-20-fr-5-10-diAc
EXPNO                  10
PROCNO                  1

F2 - Acquisition Parameters
Date_            20090930
Time                 5.43
INSTRUM             spect
PROBHD   5 mm PABBO BB-
PULPROG              zg30
TD                  65536
SOLVENT             CDCl3
NS                     16
DS                      2
SWH             8223.685 Hz
FIDRES          0.125483 Hz
AQ             3.9846387 sec
RG                    161
DW                 60.800 usec
DE                   6.50 usec
TE                  297.3 K
D1             1.00000000 sec
TD0                     1

======== CHANNEL f1 ========
NUC1                   1H
P1                   8.90 usec
PL1                 -4.00 dB
PL1W          24.73352814 W
SFO1          400.1324710 MHz

F2 - Processing parameters
SI                  32768
SF            400.1300097 MHz
WDW                    EM
SSB                     0
LB                   0.30 Hz
GB                      0
PC                   1.00
```

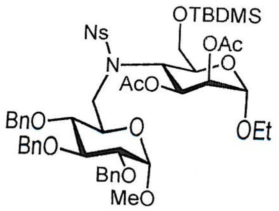

18

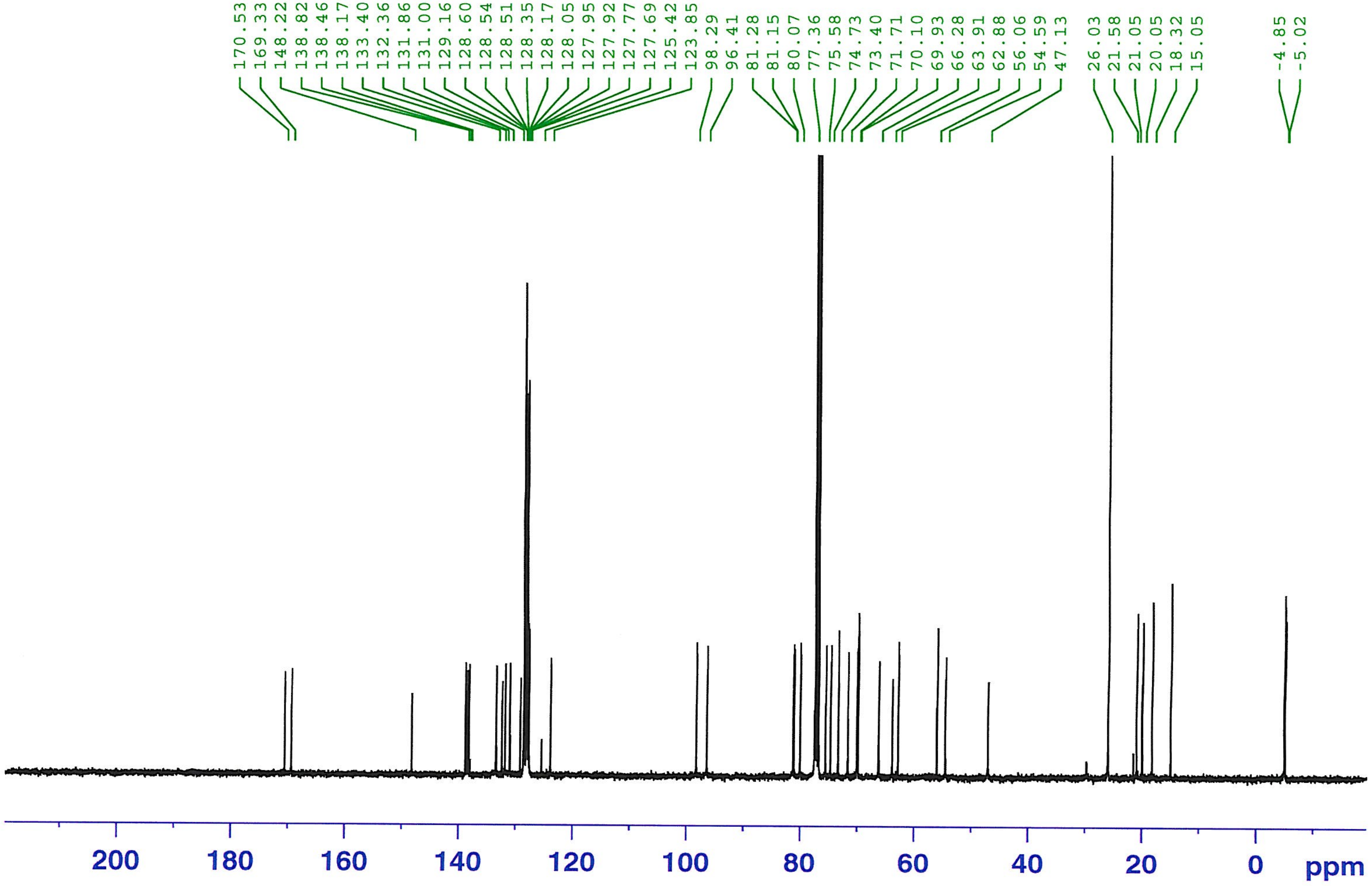


```
Current Data Parameters
NAME      JF-32-20-fr-5-10-diAc
EXPNO                  30
PROCNO                  1

F2 - Acquisition Parameters
Date_            20090930
Time                14.05
INSTRUM             spect
PROBHD   5 mm PABBO BB-
PULPROG           zgpg30
TD                  65536
SOLVENT             CDCl3
NS                   3072
DS                      4
SWH             24038.461 Hz
FIDRES           0.366798 Hz
AQ              1.3631988 sec
RG                   2050
DW                 20.800 usec
DE                  51.93 usec
TE                  298.4 K
D1             2.00000000 sec
D11            0.03000000 sec
TD0                     1

======== CHANNEL f1 ========
NUC1                  13C
P1                   6.43 usec
PL1                 -3.00 dB
PL1W          69.66502380 W
SFO1         100.6228298 MHz

======== CHANNEL f2 ========
CPDPRG2           waltz16
NUC2                   1H
PCPD2               80.00 usec
PL2                 16.00 dB
PL12                15.00 dB
PL13                15.00 dB
PL2W           0.24733528 W
PL12W          0.31137666 W
PL13W          0.31137666 W
SFO2         400.1316005 MHz

F2 - Processing parameters
SI                  32768
SF           100.6127580 MHz
WDW                    EM
SSB                     0
LB                   1.00 Hz
GB                      0
PC                   1.40
```

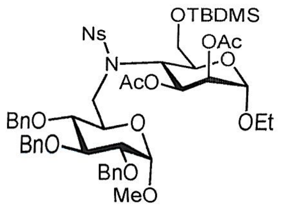

18

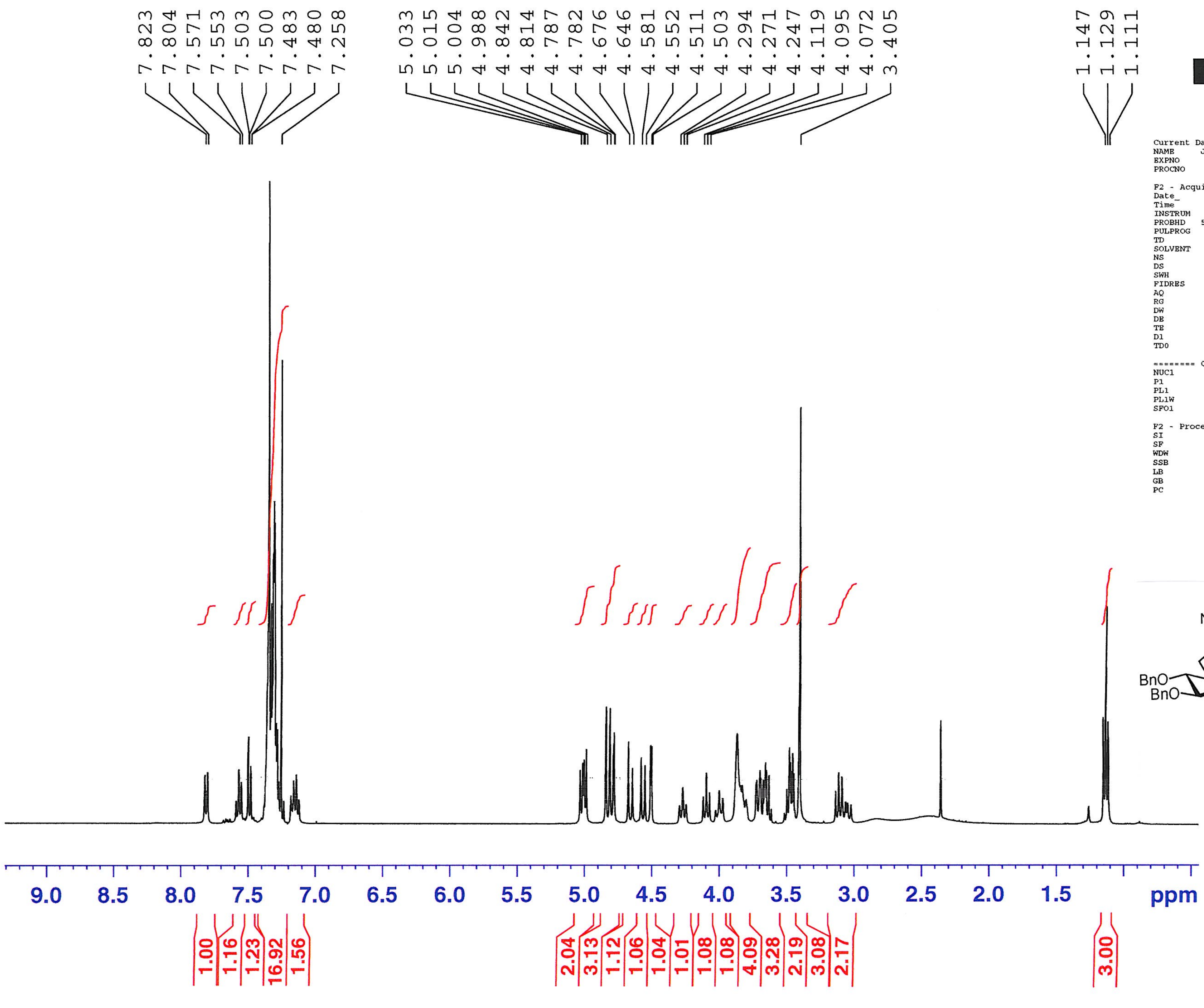

BRUKER

```
Current Data Parameters
NAME        JF-32-26-fr-15-48-triol-oso4
EXPNO                 10
PROCNO                 1

F2 - Acquisition Parameters
Date_           20091021
Time                1.34
INSTRUM            spect
PROBHD   5 mm PABBO BB-
PULPROG             zg30
TD                 65536
SOLVENT            CDCl3
NS                    16
DS                     2
SWH             8223.685 Hz
FIDRES          0.125483 Hz
AQ             3.9846387 sec
RG                   256
DW                60.800 usec
DE                  6.50 usec
TE                 297.2 K
D1            1.00000000 sec
TD0                    1

======== CHANNEL f1 ========
NUC1                  1H
P1                  8.90 usec
PL1                -4.00 dB
PL1W          24.7335281 W
SFO1         400.1324710 MHz

F2 - Processing parameters
SI                 32768
SF           400.1300104 MHz
WDW                   EM
SSB                    0
LB                  0.30 Hz
GB                     0
PC                  1.00
```

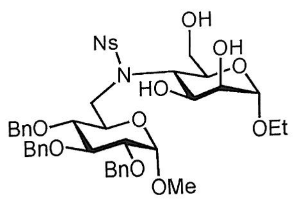

19

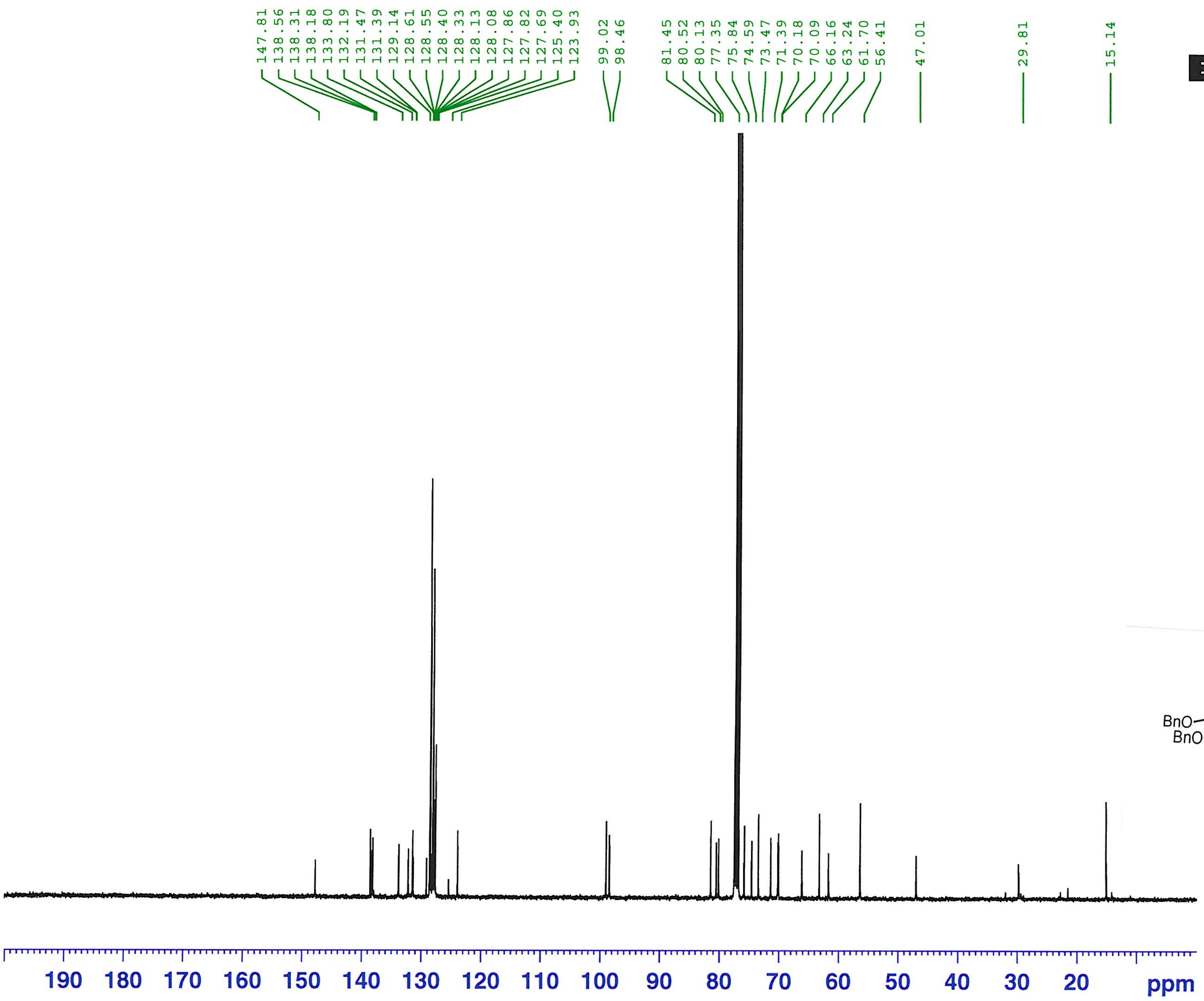

BRUKER

```
Current Data Parameters
NAME       JP-38-30-fr-7-30-desilylation
EXPNO                    20
PROCNO                    1

F2 - Acquisition Parameters
Date_              20101006
Time                  14.02
INSTRUM               spect
PROBHD   5 mm PABBO BB-
PULPROG              zgpg30
TD                    65536
SOLVENT               CDCl3
NS                     3072
DS                        4
SWH               24038.461 Hz
FIDRES             0.366798 Hz
AQ                1.3631988 sec
RG                     2050
DW                   20.800 usec
DE                    51.93 usec
TE                    298.2 K
D1               2.00000000 sec
D11              0.03000000 sec
TD0                       1

======== CHANNEL f1 ========
NUC1                    13C
P1                     6.43 usec
PL1                   -3.00 dB
PL1W            69.66502380 W
SFO1            100.6228298 MHz

======== CHANNEL f2 ========
CPDPRG2             waltz16
NUC2                     1H
PCPD2                 80.00 usec
PL2                   16.00 dB
PL12                  15.00 dB
PL13                  15.00 dB
PL2W             0.24733528 W
PL12W            0.31137666 W
PL13W            0.31137666 W
SFO2            400.1316005 MHz

F2 - Processing parameters
SI                    32768
SF              100.6127595 MHz
WDW                      EM
SSB                       0
LB                     1.00 Hz
GB                        0
PC                     1.40
```

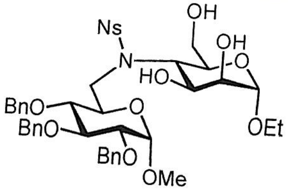

19

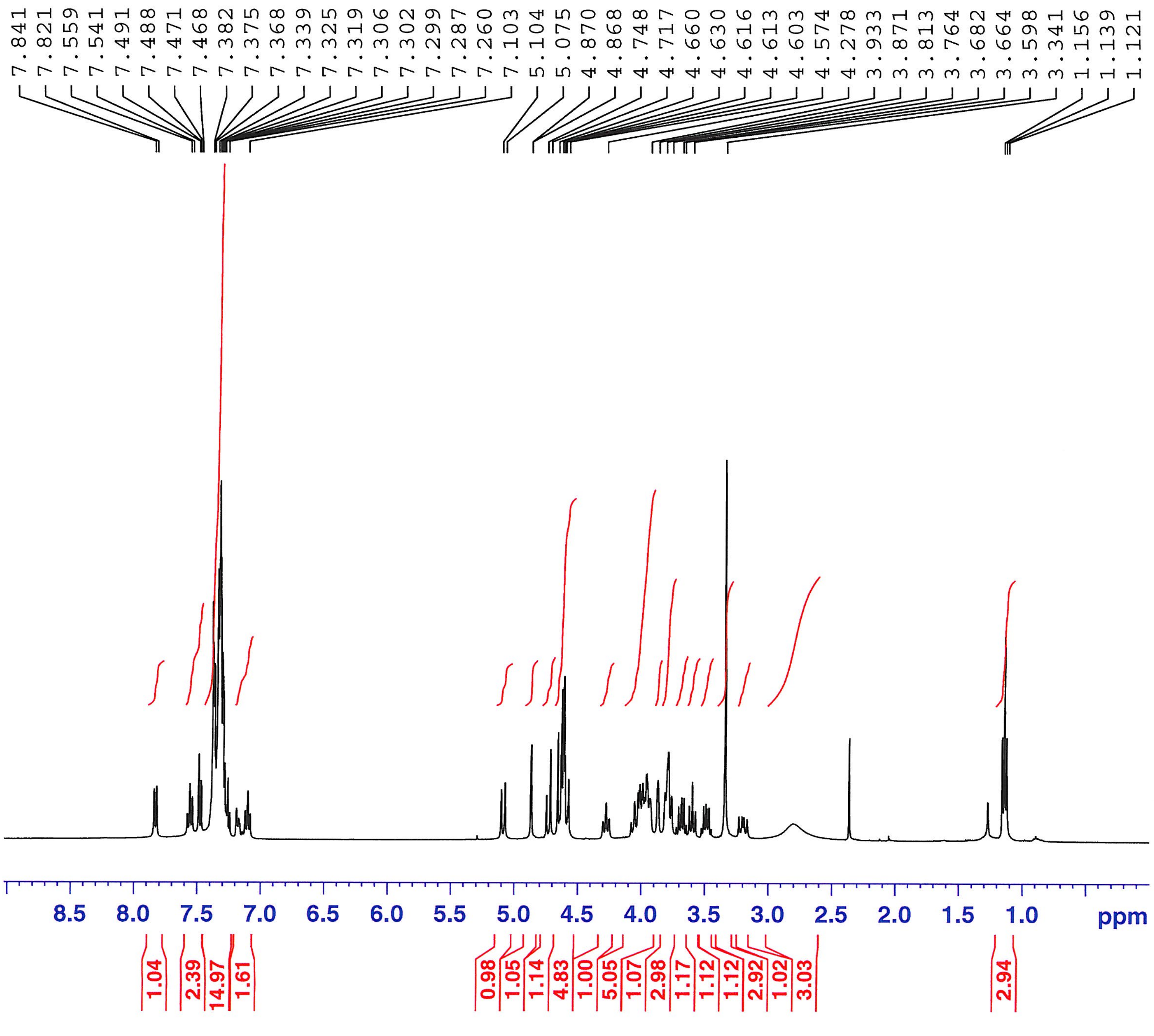

BRUKER

```
Current Data Parameters
NAME      JF-38-08-fr-8-20-desilylation
EXPNO                    10
PROCNO                    1

F2 - Acquisition Parameters
Date_              20100520
Time                  10.23
INSTRUM               spect
PROBHD   5 mm PABBO BB-
PULPROG                zg30
TD                    65536
SOLVENT               CDCl3
NS                       16
DS                        2
SWH                8223.685 Hz
FIDRES             0.125483 Hz
AQ                3.9846387 sec
RG                      161
DW                   60.800 usec
DE                     6.50 usec
TE                    298.0 K
D1               1.00000000 sec
TD0                       1

======== CHANNEL f1 ========
NUC1                     1H
P1                     8.90 usec
PL1                   -4.00 dB
PL1W            24.73352814 W
SFO1            400.1324710 MHz

F2 - Processing parameters
SI                    32768
SF              400.1300095 MHz
WDW                      EM
SSB                       0
LB                     0.30 Hz
GB                        0
PC                     1.00
```

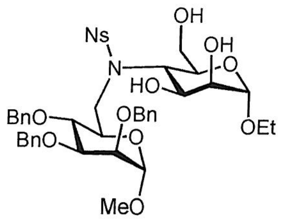

20

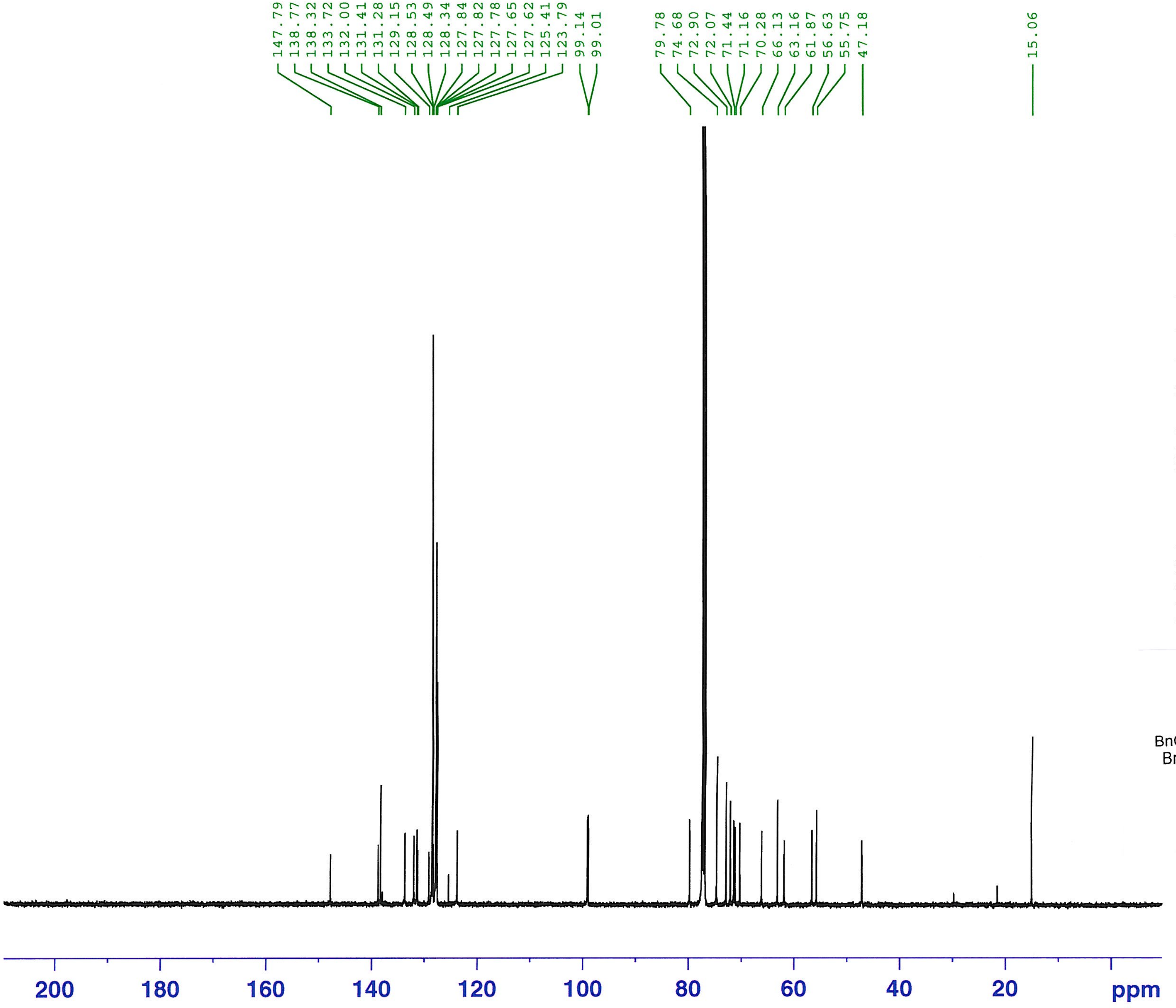


```
Current Data Parameters
NAME      JF-38-08-main-desilylation
EXPNO                 30
PROCNO                10

F2 - Acquisition Parameters
Date_           20100521
Time                6.37
INSTRUM            spect
PROBHD   5 mm PABBO BB-
PULPROG          zgpg30
TD                32768
SOLVENT           CDCl3
NS                 2048
DS                    0
SWH           27573.529 Hz
FIDRES         0.841477 Hz
AQ            0.5942430 sec
RG                 2050
DW               18.133 usec
DE                12.00 usec
TE                298.0 K
D1           2.00000000 sec
D11          0.03000000 sec
TD0                   1

======== CHANNEL f1 ========
NUC1                13C
P1                 7.50 usec
PL1                0.00 dB
PL1W        83.89700317 W
SFO1        125.7703648 MHz

======== CHANNEL f2 ========
CPDPRG2         waltz16
NUC2                 1H
PCPD2             80.00 usec
PL2                2.00 dB
PL12              18.62 dB
PL13              21.00 dB
PL2W        15.76968765 W
PL12W        0.34341794 W
PL13W        0.19852860 W
SFO2        500.1321306 MHz

F2 - Processing parameters
SI                32768
SF          125.7577778 MHz
WDW                  EM
SSB                   0
LB                 1.00 Hz
GB                    0
PC                 1.40
```

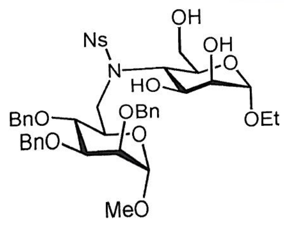

20

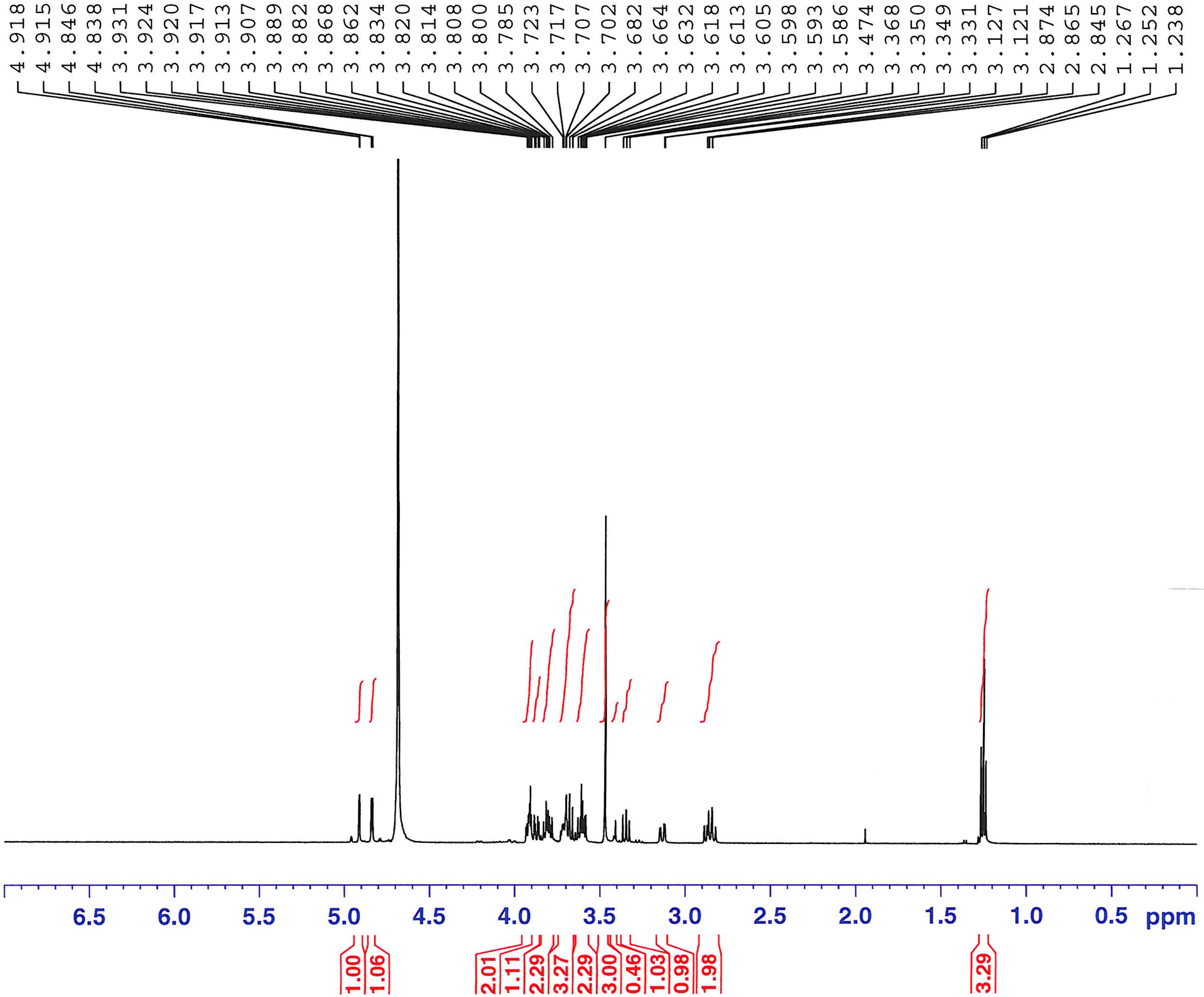

Current Data Parameters

NAME	JF-32-44-FL2-fr-28-33
EXPNO	10
PROCNO	10

F2 - Acquisition Parameters

Date_	20100906
Time	2.40
INSTRUM	spect
PROBHD	5 mm PABBO BB-
PULPROG	zg30
TD	65536
SOLVENT	D2O
NS	16
DS	0
SWH	8012.820 Hz
FIDRES	0.122266 Hz
AQ	4.0894966 sec
RG	203
DW	62.400 usec
DE	6.00 usec
TE	308.2 K
D1	2.00000000 sec
TD0	1

======== CHANNEL f1 ========

NUC1	1H
P1	11.80 usec
PL1	2.00 dB
PL1W	15.76968765 W
SFO1	500.1325007 MHz

F2 - Processing parameters

SI	32768
SF	500.1300065 MHz
WDW	EM
SSB	0
LB	-0.10 Hz
GB	0
PC	1.00

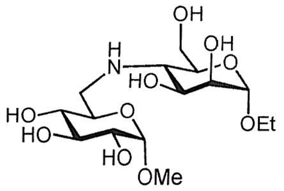

21

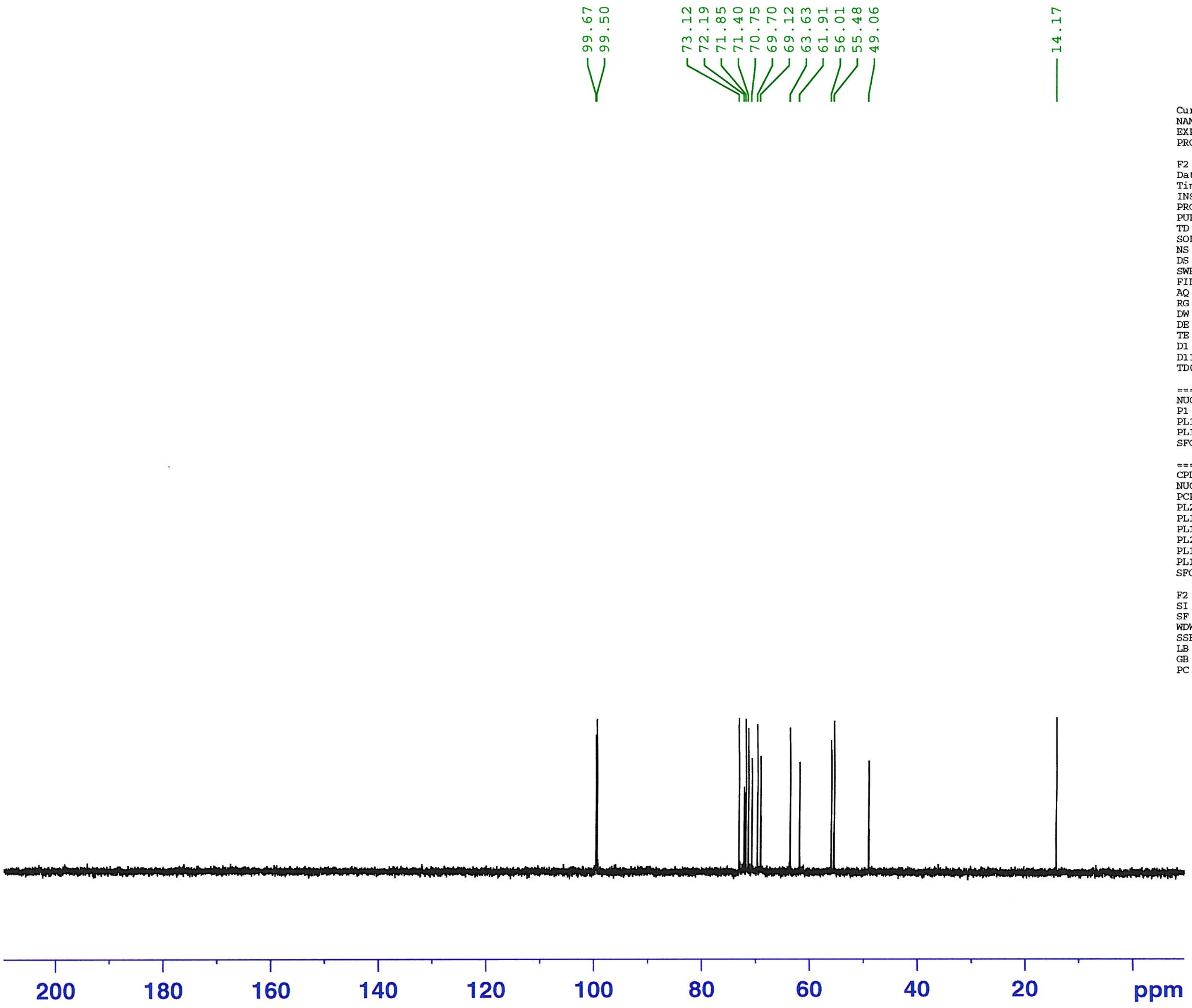


```
Current Data Parameters
NAME      JF-32-44-FL2-fr-28-33
EXPNO                  40
PROCNO                 10

F2 - Acquisition Parameters
Date_            20100906
Time                 4.01
INSTRUM             spect
PROBHD   5 mm PABBO BB-
PULPROG            zgpg30
TD                  32768
SOLVENT               D2O
NS                   1536
DS                      0
SWH             27573.529 Hz
FIDRES           0.841477 Hz
AQ              0.5942430 sec
RG                   2050
DW                 18.133 usec
DE                  12.00 usec
TE                  308.2 K
D1             2.00000000 sec
D11            0.03000000 sec
TD0                     6

======== CHANNEL f1 ========
NUC1                  13C
P1                   7.50 usec
PL1                  0.00 dB
PL1W          83.89700317 W
SFO1          125.7703648 MHz

======== CHANNEL f2 ========
CPDPRG2           waltz16
NUC2                   1H
PCPD2               80.00 usec
PL2                  2.00 dB
PL12                18.62 dB
PL13                21.00 dB
PL2W          15.76968765 W
PL12W          0.34341794 W
PL13W          0.19852860 W
SFO2          500.1321306 MHz

F2 - Processing parameters
SI                  32768
SF            125.7577839 MHz
WDW                    EM
SSB                     0
LB                   1.00 Hz
GB                      0
PC                   1.40
```

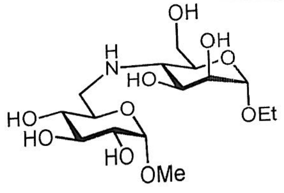

21

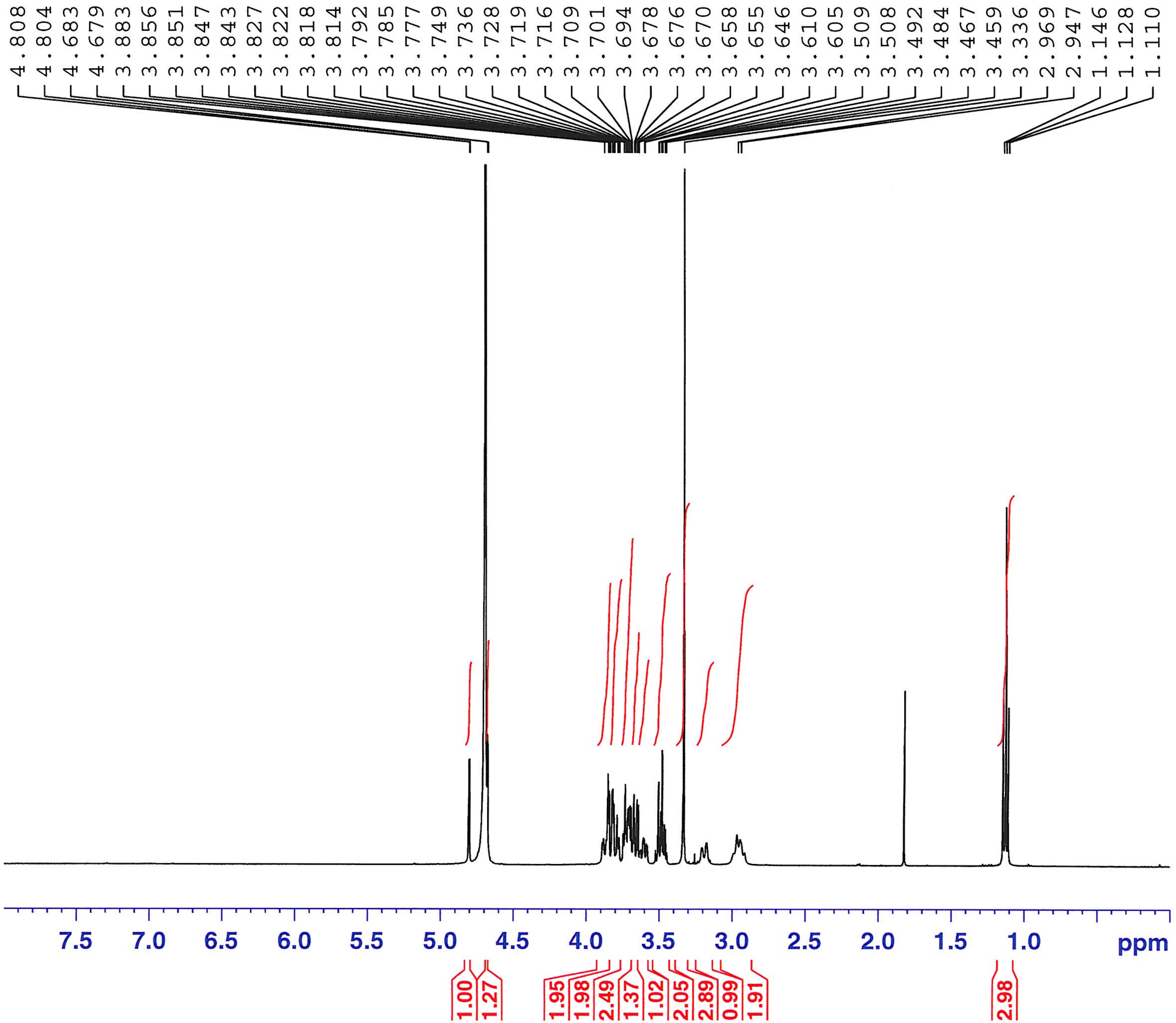

Current Data Parameters	
NAME	JF-38-18-FL3-fr-8-12
EXPNO	10
PROCNO	1
F2 - Acquisition Parameters	
Date_	20100906
Time	15.48
INSTRUM	spect
PROBHD	5 mm PABBO BB-
PULPROG	zg30
TD	65788
SOLVENT	D2O
NS	64
DS	0
SWH	8223.685 Hz
FIDRES	0.125003 Hz
AQ	3.9999604 sec
RG	287
DW	60.800 usec
DE	6.00 usec
TE	297.0 K
D1	2.00000000 sec
TD0	1
======== CHANNEL f1 ========	
NUC1	1H
P1	8.90 usec
PL1	-4.00 dB
PL1W	24.73352814 W
SFO1	400.1320007 MHz
F2 - Processing parameters	
SI	32768
SF	400.1300000 MHz
WDW	EM
SSB	0
LB	0.30 Hz
GB	0
PC	1.00

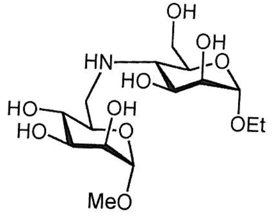

22

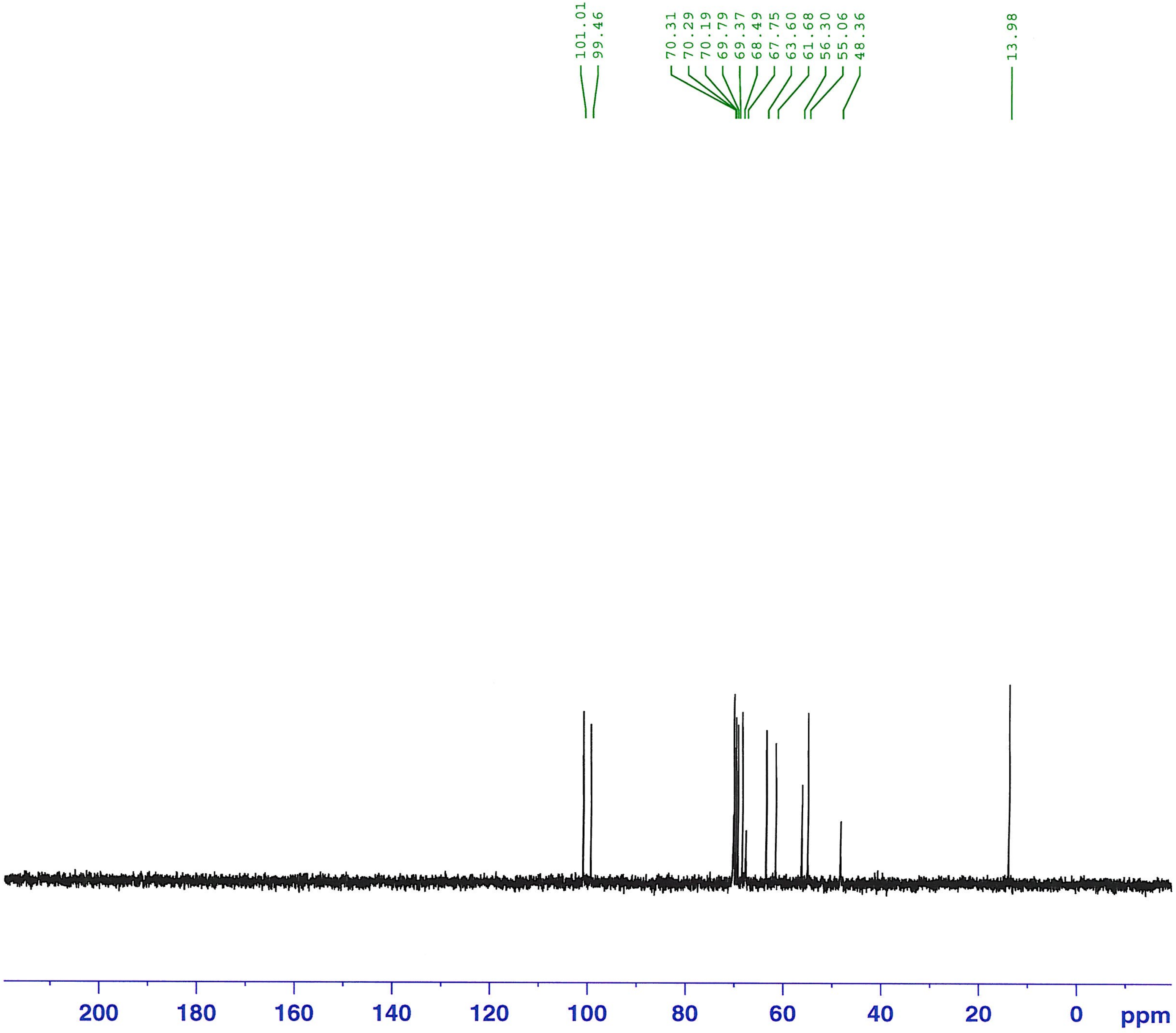

BRUKER

```
Current Data Parameters
NAME     JF-38-18-FL3-fr-8-12
EXPNO                  20
PROCNO                  1

F2 - Acquisition Parameters
Date_            20100906
Time                18.47
INSTRUM             spect
PROBHD   5 mm PABBO BB-
PULPROG           zgpg30
TD                 65536
SOLVENT              D2O
NS                  3072
DS                     4
SWH            24038.461 Hz
FIDRES          0.366798 Hz
AQ             1.3631988 sec
RG                  2050
DW                20.800 usec
DE                 51.93 usec
TE                 297.7 K
D1            2.00000000 sec
D11           0.03000000 sec
TD0                    1

======== CHANNEL f1 ========
NUC1                 13C
P1                  6.43 usec
PL1                -3.00 dB
PL1W         69.66502380 W
SFO1         100.6228298 MHz

======== CHANNEL f2 ========
CPDPRG2          waltz16
NUC2                  1H
PCPD2              80.00 usec
PL2                16.00 dB
PL12               15.00 dB
PL13               15.00 dB
PL2W          0.24733528 W
PL12W         0.31137666 W
PL13W         0.31137666 W
SFO2         400.1316005 MHz

F2 - Processing parameters
SI                 32768
SF           100.6127690 MHz
WDW                   EM
SSB                    0
LB                  2.00 Hz
GB                     0
PC                  1.40
```

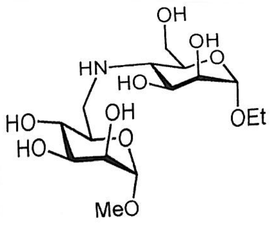

22

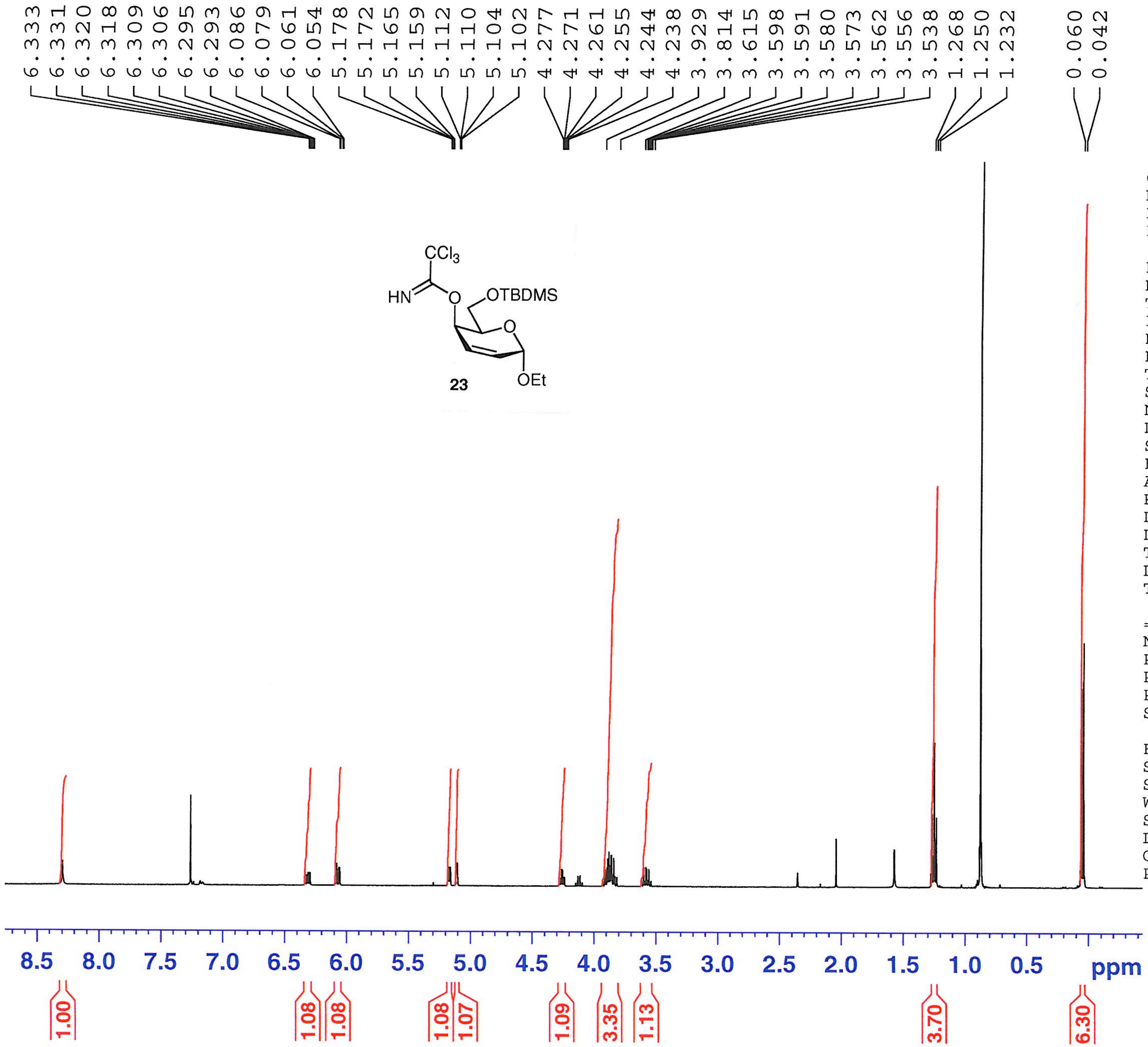

Current Data Parameters
NAME PEP-30-30-t-9-23
EXPNO 10
PROCNO 1

F2 - Acquisition Parameters
Date_ 20090525
Time 8.58
INSTRUM spect
PROBHD 5 mm PABBO BB-
PULPROG zg30
TD 65536
SOLVENT CDCl3
NS 16
DS 2
SWH 8223.685 Hz
FIDRES 0.125483 Hz
AQ 3.9846387 sec
RG 181
DW 60.800 usec
DE 6.50 usec
TE 298.3 K
D1 1.00000000 sec
TD0 1

======== CHANNEL f1 ========
NUC1 1H
P1 8.90 usec
PL1 -4.00 dB
PL1W 24.73352814 W
SFO1 400.1324710 MHz

F2 - Processing parameters
SI 32768
SF 400.1300094 MHz
WDW EM
SSB 0
LB 0.30 Hz
GB 0
PC 1.00

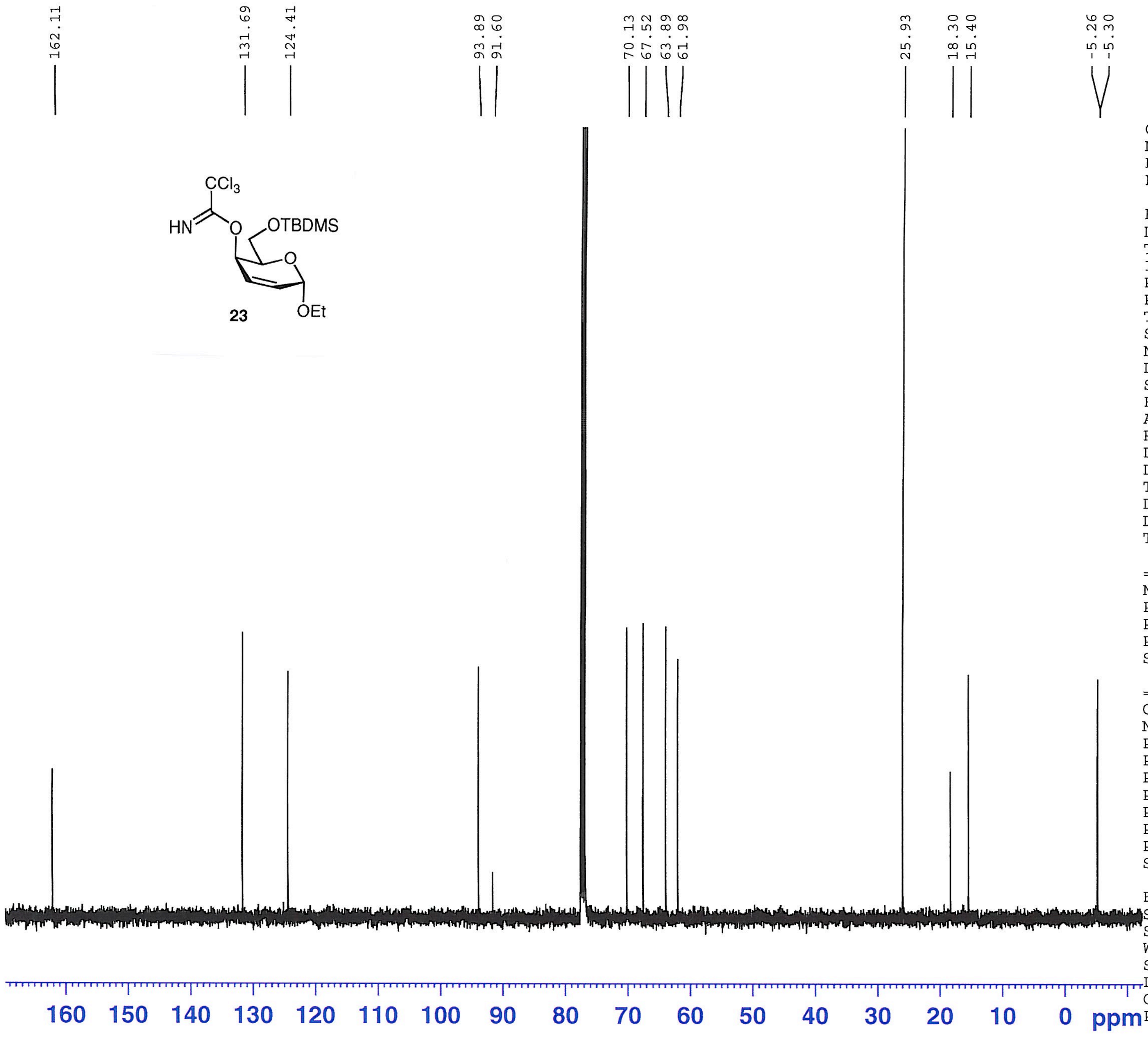

Current Data Parameters
NAME PEP-30-30
EXPNO 10
PROCNO 1

F2 - Acquisition Parameters
Date_ 20090526
Time 20.06
INSTRUM spect
PROBHD 5 mm PABBO BB-
PULPROG zgpg30
TD 65536
SOLVENT CDCl3
NS 1024
DS 4
SWH 24038.461 Hz
FIDRES 0.366798 Hz
AQ 1.3631988 sec
RG 2050
DW 20.800 usec
DE 51.93 usec
TE 299.5 K
D1 2.00000000 sec
D11 0.03000000 sec
TD0 1

======== CHANNEL f1 ========
NUC1 13C
P1 6.43 usec
PL1 -3.00 dB
PL1W 69.66502380 W
SFO1 100.6228298 MHz

======== CHANNEL f2 ========
CPDPRG2 waltz16
NUC2 1H
PCPD2 80.00 usec
PL2 16.00 dB
PL12 15.00 dB
PL13 15.00 dB
PL2W 0.24733528 W
PL12W 0.31137666 W
PL13W 0.31137666 W
SFO2 400.1316005 MHz

F2 - Processing parameters
SI 32768
SF 100.6127534 MHz
WDW EM
SSB 0
LB 1.00 Hz
GB 0
PC 1.40

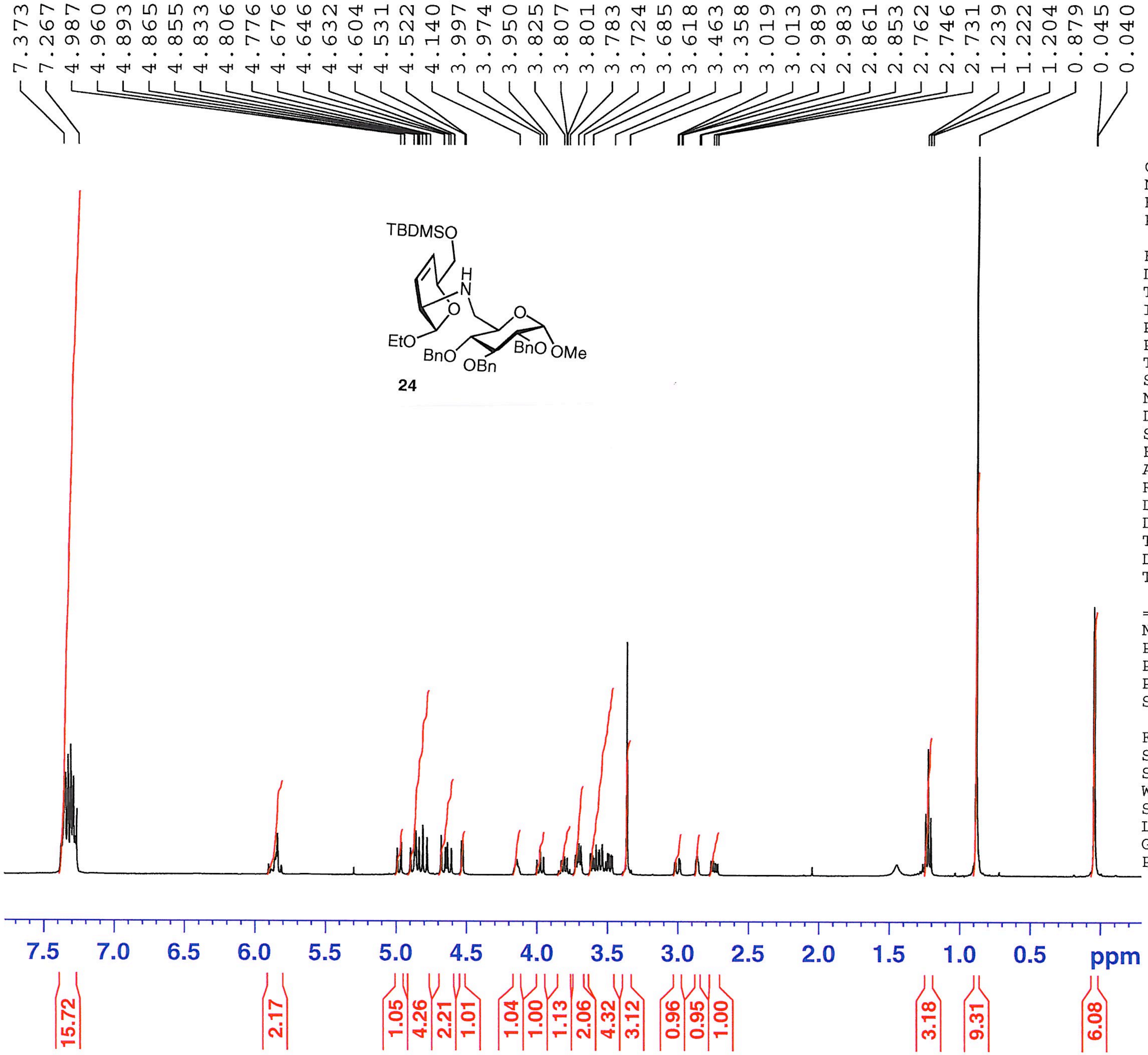


```
Current Data Parameters
NAME          PEP-30-31-t-33-49
EXPNO                   10
PROCNO                   1

F2 - Acquisition Parameters
Date_             20090528
Time                 20.01
INSTRUM              spect
PROBHD    5 mm PABBO BB-
PULPROG               zg30
TD                   65536
SOLVENT              CDCl3
NS                      16
DS                       2
SWH               8223.685 Hz
FIDRES            0.125483 Hz
AQ               3.9846387 sec
RG                     114
DW                  60.800 usec
DE                    6.50 usec
TE                   297.3 K
D1              1.00000000 sec
TD0                      1

======== CHANNEL f1 ========
NUC1                    1H
P1                    8.90 usec
PL1                  -4.00 dB
PL1W           24.73352814 W
SFO1           400.1324710 MHz

F2 - Processing parameters
SI                   32768
SF             400.1300094 MHz
WDW                     EM
SSB                      0
LB                    0.30 Hz
GB                       0
PC                    1.00
```

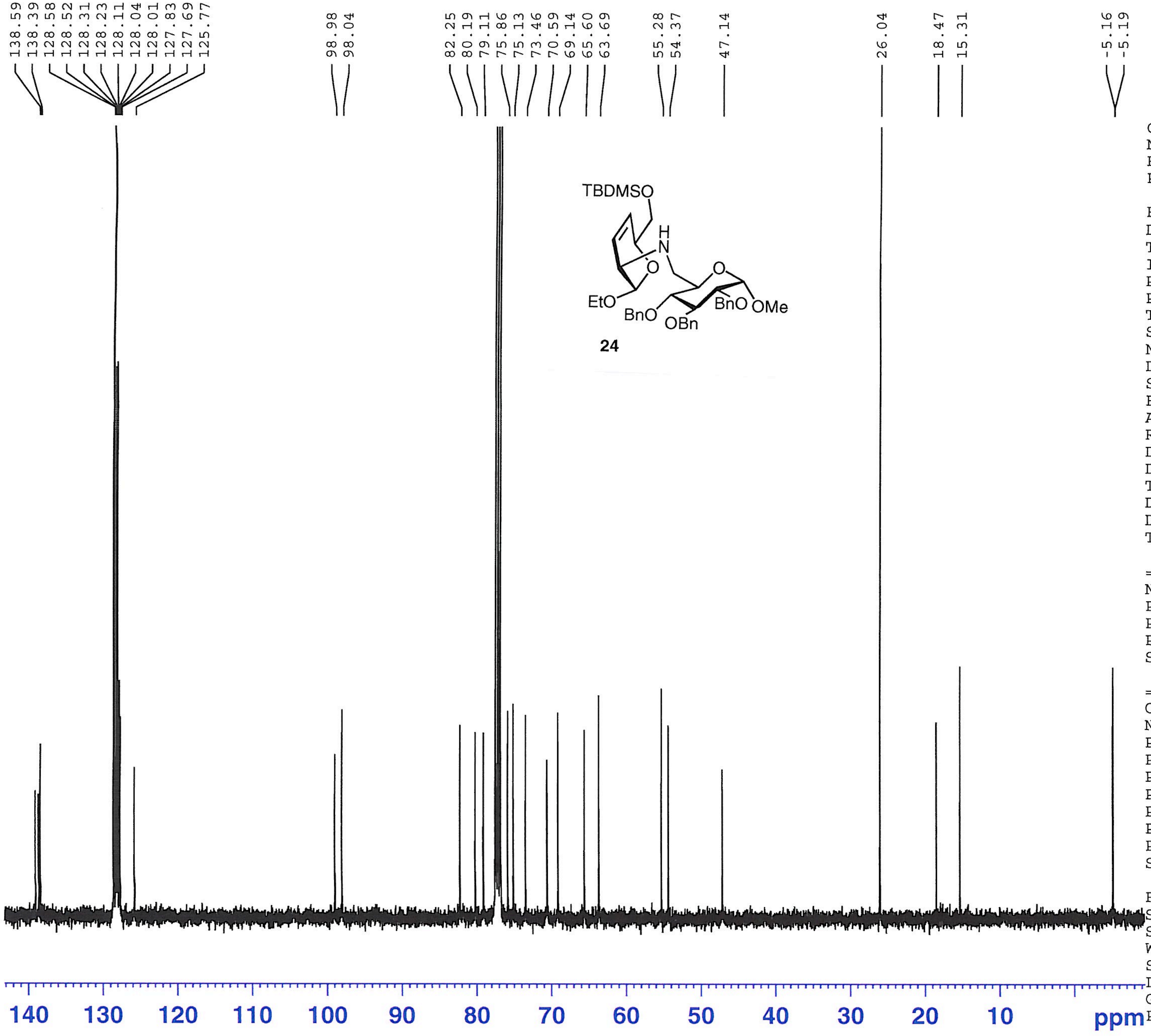

24

```
Current Data Parameters
NAME          PEP-30-31-t-33-49
EXPNO                        11
PROCNO                        1

F2 - Acquisition Parameters
Date_                  20090528
Time                      21.01
INSTRUM                   spect
PROBHD       5 mm PABBO BB-
PULPROG                 zgpg30
TD                        65536
SOLVENT                   CDCl3
NS                         1024
DS                            4
SWH                   24038.461 Hz
FIDRES                 0.366798 Hz
AQ                    1.3631988 sec
RG                         2050
DW                       20.800 usec
DE                        51.93 usec
TE                        299.3 K
D1                   2.00000000 sec
D11                  0.03000000 sec
TD0                           1

======== CHANNEL f1 ========
NUC1                        13C
P1                         6.43 usec
PL1                       -3.00 dB
PL1W                69.66502380 W
SFO1                100.6228298 MHz

======== CHANNEL f2 ========
CPDPRG2                 waltz16
NUC2                         1H
PCPD2                     80.00 usec
PL2                       16.00 dB
PL12                      15.00 dB
PL13                      15.00 dB
PL2W                 0.24733528 W
PL12W                0.31137666 W
PL13W                0.31137666 W
SFO2                400.1316005 MHz

F2 - Processing parameters
SI                        32768
SF                  100.6127553 MHz
WDW                          EM
SSB                           0
LB                         1.00 Hz
GB                            0
PC                         1.40
```

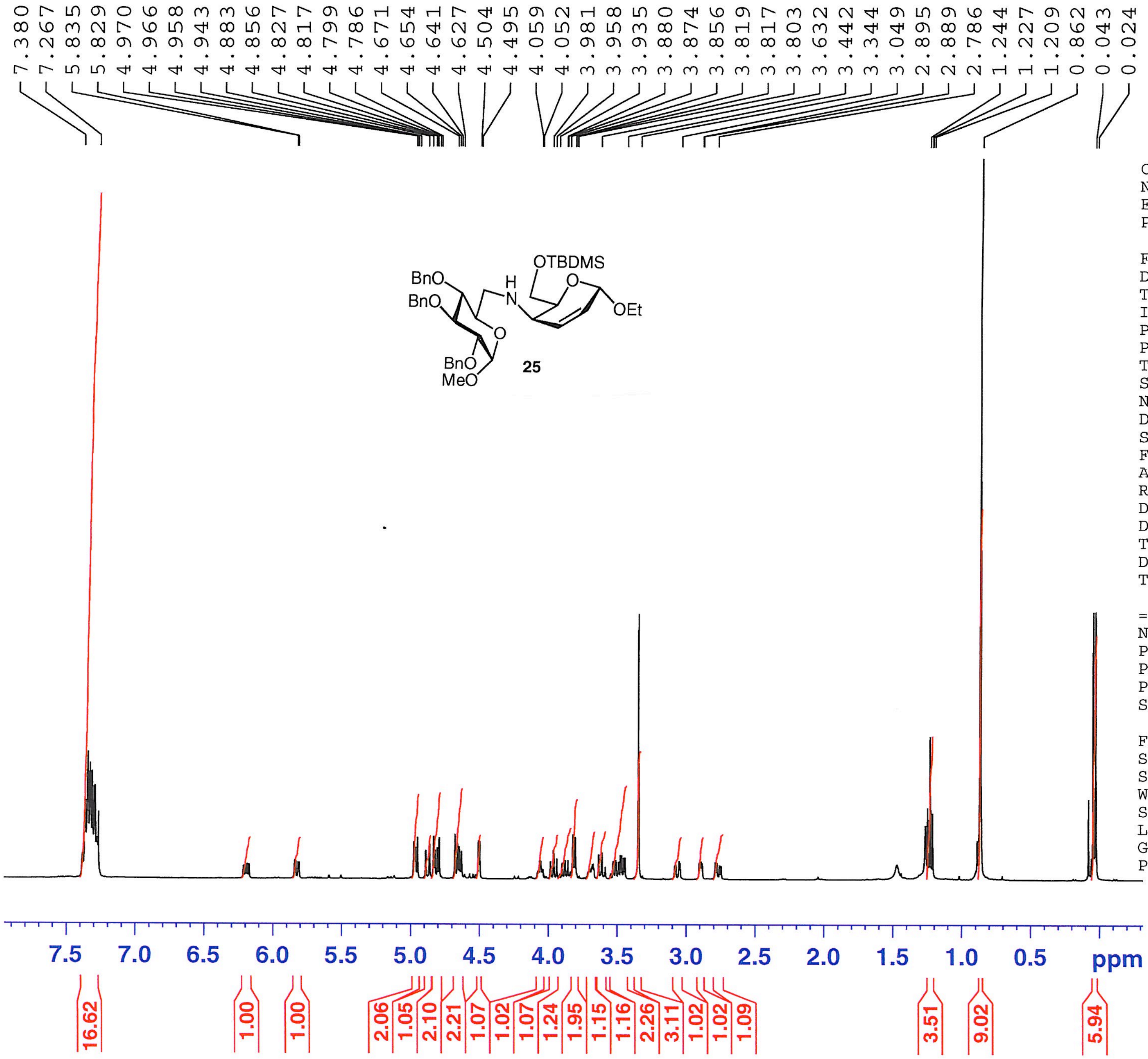

Current Data Parameters		
NAME	PEP-30-33-k2-27-51	
EXPNO	10	
PROCNO	1	
F2 - Acquisition Parameters		
Date_	20090605	
Time	8.57	
INSTRUM	spect	
PROBHD	5 mm PABBO BB-	
PULPROG	zg30	
TD	65536	
SOLVENT	CDCl3	
NS	16	
DS	2	
SWH	8223.685	Hz
FIDRES	0.125483	Hz
AQ	3.9846387	sec
RG	114	
DW	60.800	usec
DE	6.50	usec
TE	297.0	K
D1	1.00000000	sec
TD0	1	
======== CHANNEL f1 ========		
NUC1	1H	
P1	8.90	usec
PL1	-4.00	dB
PL1W	24.73352814	W
SFO1	400.1324710	MHz
F2 - Processing parameters		
SI	32768	
SF	400.1300094	MHz
WDW	EM	
SSB	0	
LB	0.30	Hz
GB	0	
PC	1.00	

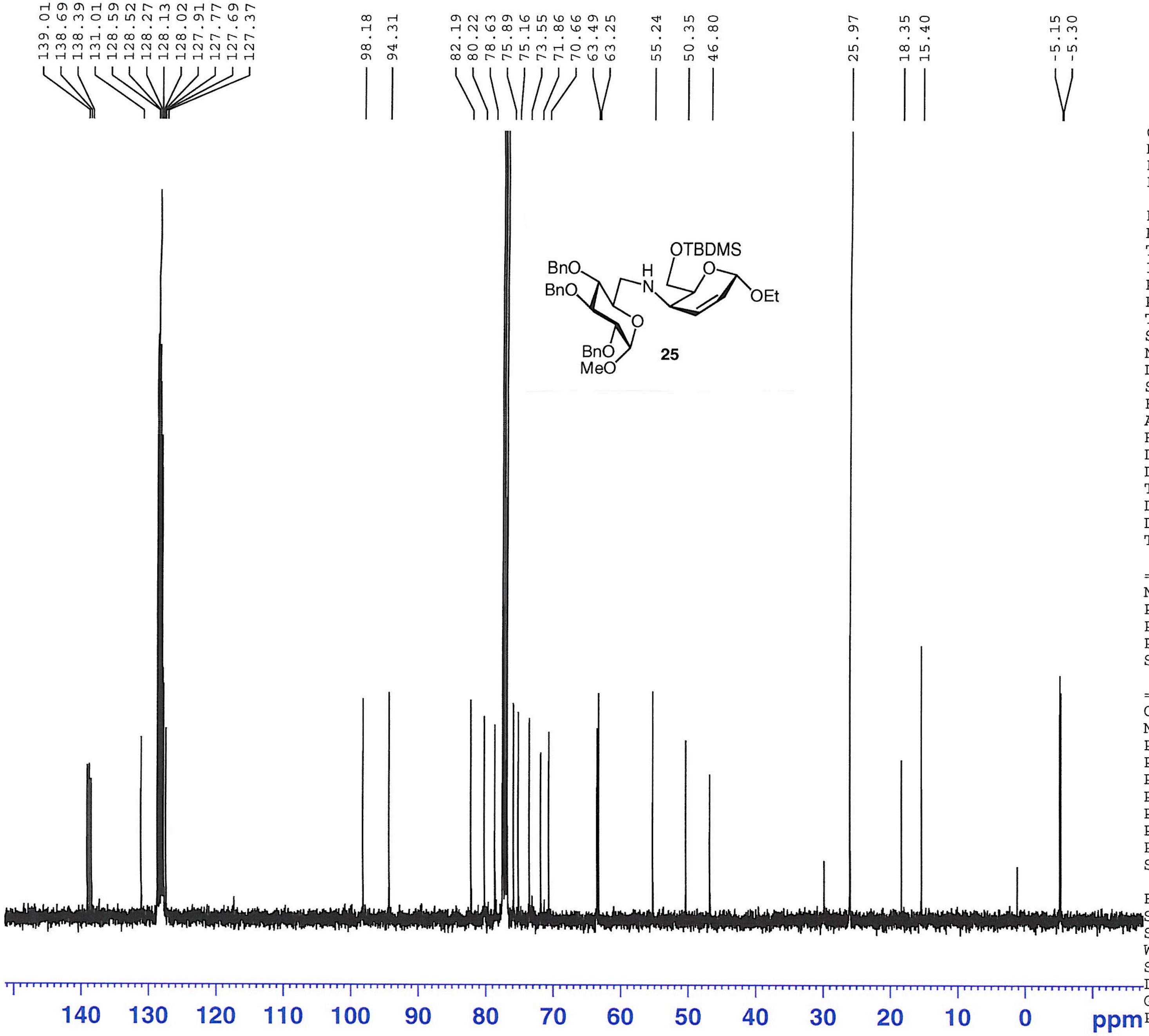

Current Data Parameters

NAME	PEP-30-33-k2-27-51
EXPNO	12
PROCNO	1

F2 - Acquisition Parameters

Date_	20090605
Time	21.22
INSTRUM	spect
PROBHD	5 mm PABBO BB-
PULPROG	zgpg30
TD	65536
SOLVENT	CDCl3
NS	1024
DS	4
SWH	24038.461 Hz
FIDRES	0.366798 Hz
AQ	1.3631988 sec
RG	2050
DW	20.800 usec
DE	51.93 usec
TE	299.2 K
D1	2.00000000 sec
D11	0.03000000 sec
TD0	1

======== CHANNEL f1 ========

NUC1	13C
P1	6.43 usec
PL1	-3.00 dB
PL1W	69.66502380 W
SFO1	100.6228298 MHz

======== CHANNEL f2 ========

CPDPRG2	waltz16
NUC2	1H
PCPD2	80.00 usec
PL2	16.00 dB
PL12	15.00 dB
PL13	15.00 dB
PL2W	0.24733528 W
PL12W	0.31137666 W
PL13W	0.31137666 W
SFO2	400.1316005 MHz

F2 - Processing parameters

SI	32768
SF	100.6127559 MHz
WDW	EM
SSB	0
LB	1.00 Hz
GB	0
PC	1.40

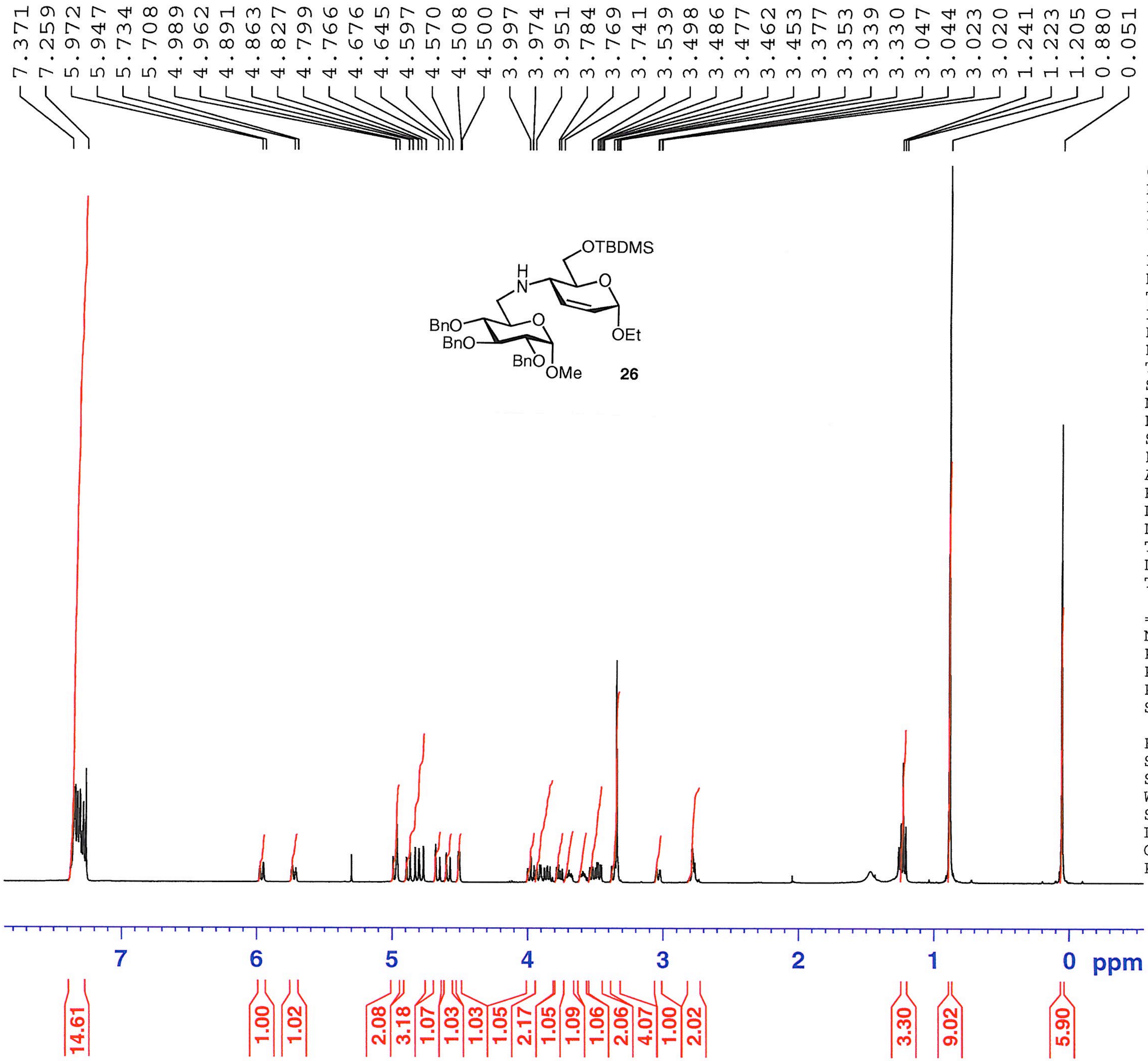

Current Data Parameters
NAME PEP-30-33-k2-16-25
EXPNO 10
PROCNO 1

F2 - Acquisition Parameters
Date_ 20090604
Time 6.18
INSTRUM spect
PROBHD 5 mm PABBO BB-
PULPROG zg30
TD 65536
SOLVENT CDCl3
NS 16
DS 2
SWH 8223.685 Hz
FIDRES 0.125483 Hz
AQ 3.9846387 sec
RG 181
DW 60.800 usec
DE 6.50 usec
TE 296.9 K
D1 1.00000000 sec
TD0 1

======== CHANNEL f1 ========
NUC1 1H
P1 8.90 usec
PL1 -4.00 dB
PL1W 24.73352814 W
SFO1 400.1324710 MHz

F2 - Processing parameters
SI 32768
SF 400.1300100 MHz
WDW EM
SSB 0
LB 0.30 Hz
GB 0
PC 1.00

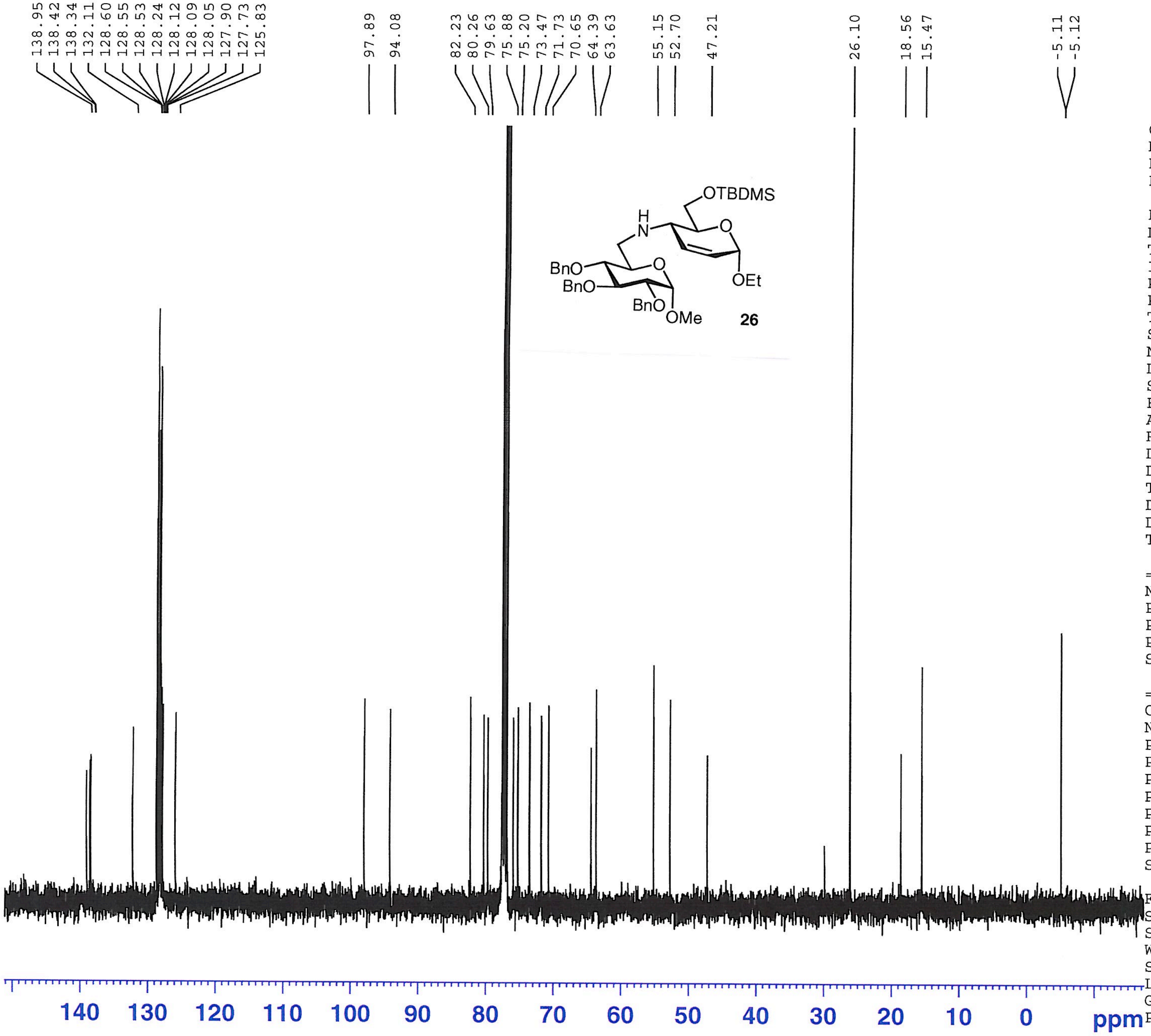

26

```
Current Data Parameters
NAME        PEP-30-33-k2-16-25
EXPNO                       12
PROCNO                       1

F2 - Acquisition Parameters
Date_               20090604
Time                   12.03
INSTRUM                spect
PROBHD     5 mm PABBO BB-
PULPROG              zgpg30
TD                     65536
SOLVENT                CDCl3
NS                      1024
DS                         4
SWH               24038.461 Hz
FIDRES             0.366798 Hz
AQ                1.3631988 sec
RG                      2050
DW                    20.800 usec
DE                     51.93 usec
TE                     299.1 K
D1                2.00000000 sec
D11               0.03000000 sec
TD0                        1

======== CHANNEL f1 ========
NUC1                     13C
P1                      6.43 usec
PL1                    -3.00 dB
PL1W             69.66502380 W
SFO1             100.6228298 MHz

======== CHANNEL f2 ========
CPDPRG2              waltz16
NUC2                      1H
PCPD2                  80.00 usec
PL2                    16.00 dB
PL12                   15.00 dB
PL13                   15.00 dB
PL2W              0.24733528 W
PL12W             0.31137666 W
PL13W             0.31137666 W
SFO2             400.1316005 MHz

F2 - Processing parameters
SI                     32768
SF               100.6127541 MHz
WDW                       EM
SSB                        0
LB                      1.00 Hz
GB                         0
PC                      1.40
```

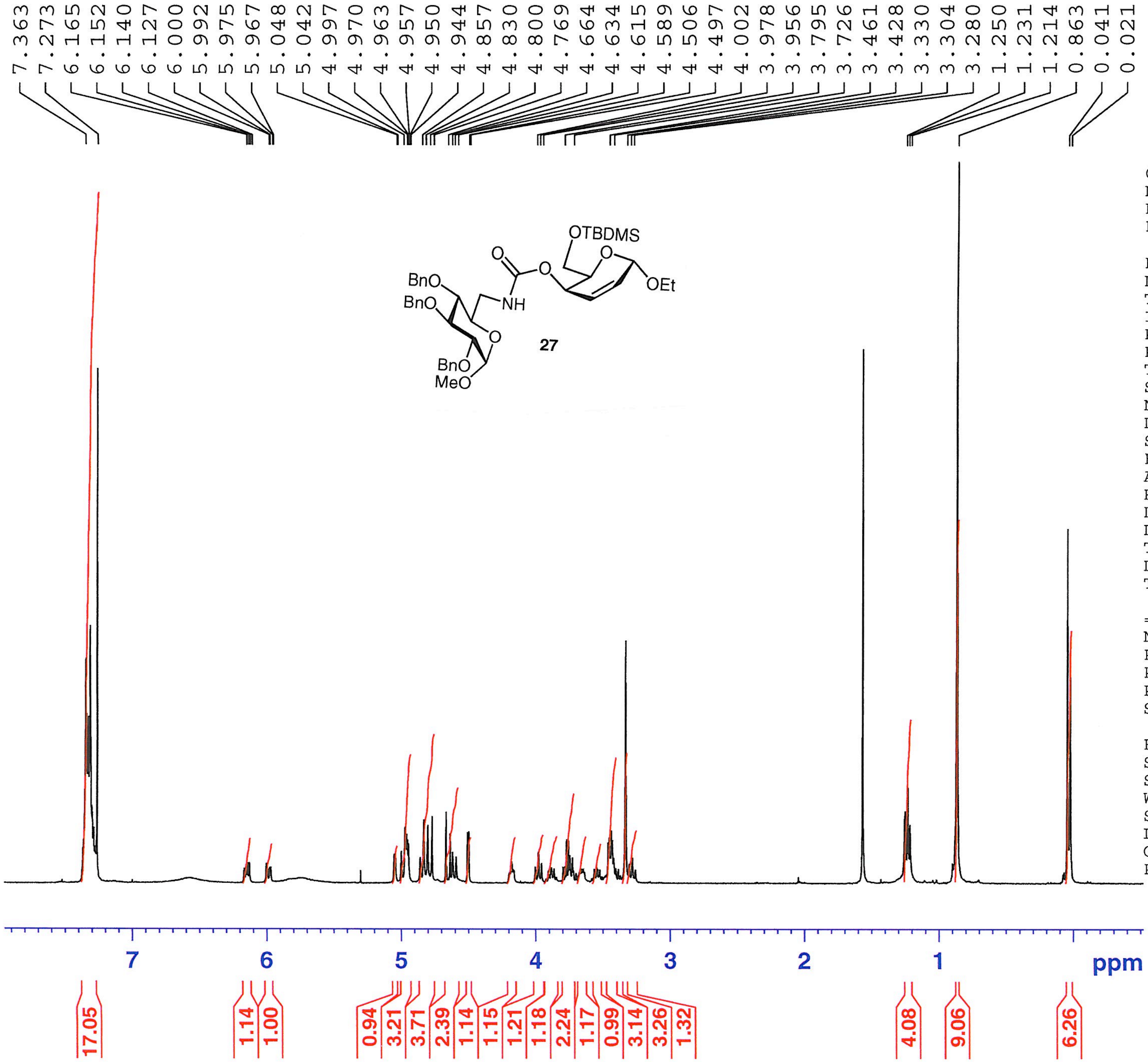

Current Data Parameters
NAME PEP-30-33-k2-7-10
EXPNO 10
PROCNO 1

F2 - Acquisition Parameters
Date_ 20090604
Time 4.57
INSTRUM spect
PROBHD 5 mm PABBO BB-
PULPROG zg30
TD 65536
SOLVENT CDCl3
NS 16
DS 2
SWH 8223.685 Hz
FIDRES 0.125483 Hz
AQ 3.9846387 sec
RG 287
DW 60.800 usec
DE 6.50 usec
TE 297.0 K
D1 1.00000000 sec
TD0 1

======== CHANNEL f1 ========
NUC1 1H
P1 8.90 usec
PL1 -4.00 dB
PL1W 24.73352814 W
SFO1 400.1324710 MHz

F2 - Processing parameters
SI 32768
SF 400.1300094 MHz
WDW EM
SSB 0
LB 0.30 Hz
GB 0
PC 1.00

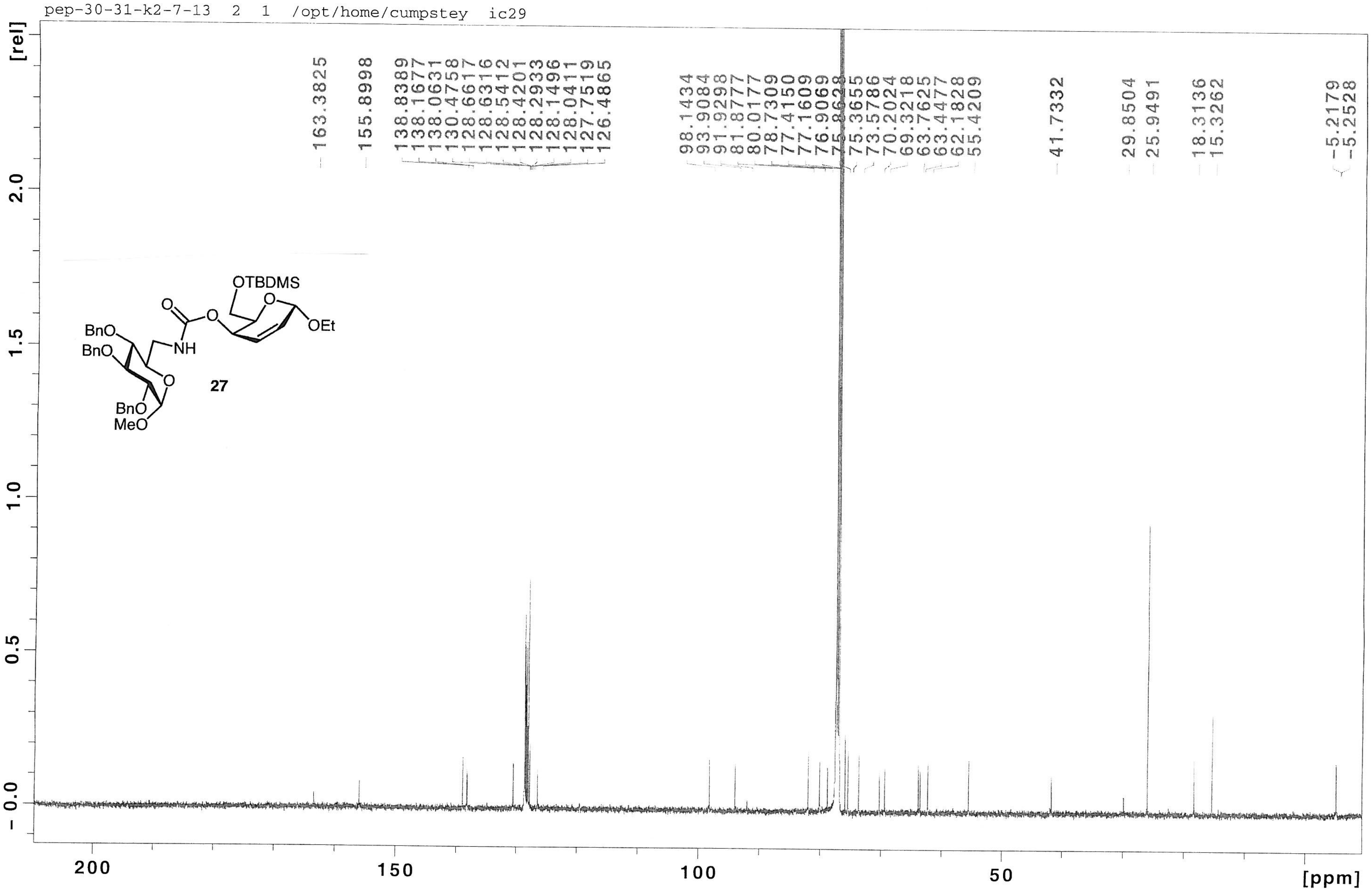
pep-30-31-k2-7-13 2 1 /opt/home/cumpstey ic29
[rel]
2.0
1.5
1.0
0.5
0.0
200
150
100
50
[ppm]
163.3825
155.8998
138.8389
138.1677
138.0631
130.4758
128.6617
128.6316
128.5412
128.4201
128.2933
128.1496
128.0411
127.7519
126.4865
98.1434
93.9084
91.9298
81.8777
80.0177
78.7309
77.4150
77.1609
76.9069
75.8628
75.3655
73.5786
70.2024
69.3218
63.7625
63.4477
62.1828
55.4209
41.7332
29.8504
25.9491
18.3136
15.3262
-5.2179
-5.2528
OTBDMS
OEt
NH
BnO
BnO
BnO
MeO
27
